# High-throughput quantification of population dynamics using luminescence

**DOI:** 10.7554/eLife.109213

**Published:** 2026-08-05

**Authors:** Malte Muetter, Daniel C Angst, Roland Regoes, Sebastian Bonhoeffer

**Affiliations:** 1 Department of Environmental Systems Science, ETH Zürich Zürich Switzerland; University College London United Kingdom; CNRS France

**Keywords:** bioluminescence, colony-forming units, pharmacodynamics, antibiotics, time-kill curves, *E. coli*

## Abstract

Bacterial population decline at antibiotic concentrations above the minimum inhibitory concentration (MIC) remains poorly characterized. This is because colony-forming units (CFU), the standard method to quantify inhibition, are slow, labor-intensive, and costly. Luminescence assays are widely used to quantify population dynamics at subinhibitory concentrations, yet their limitations and reliability at high concentrations remain underexplored. Here, we compared luminescence- and CFU-based rates in *Escherichia coli* across 20 antimicrobials. In our experiments, luminescence- and CFU-based rates did not differ significantly for half of them. For the other half, CFU-based decline rates were consistently higher. The estimates differed for two main reasons: First, because light intensity tracks biomass more closely than population size, luminescence declined more slowly than the population when bacteria filamented. Second, CFU-based estimates indicated a steeper decline when treatment reduced the number of colonies formed per plated bacterium. This can result from changes in clustering behavior, physiological changes that impair culturability, or antimicrobial carryover. Thus, the suitability of luminescence to quantify bacterial decline depends on the physiological effects of the antimicrobial and whether the quantity of interest is cell number or biomass. Within these limitations, luminescence can serve as an efficient, high-throughput alternative for quantifying bacterial dynamics at super-MIC concentrations.

## Introduction

Accurate characterization of changes in population size under treatment is essential for understanding the evolution of antibiotic resistance. Commonly, the effect of treatment on bacterial populations is quantified by pharmacodynamic (PD) curves. PD curves quantify the relationship between drug concentration and the rate of population change (net growth) (Regoes et al., 2004). These range from no antibiotic, through sub-MIC concentrations that only reduce population growth, to super-MIC concentrations that kill bacteria and lead to a population decline.

The growth parameter most often used in PD curves is the exponential rate of change in living bacteria, $\psi_{B}$, reflecting both division and death. However, other population properties, such as changes in the number of culturable bacteria or total biomass, may also be relevant, depending on the specific biological question.

In practice, PD curves are fitted to the rate of change of a measured proxy signal such as optical density (OD), colony-forming units (CFU), or bioluminescent light intensity.

OD is a cost-effective approach for real-time, high-throughput monitoring of culture turbidity without sacrificing the population. OD is positively correlated (within a certain range) to cell density. However, since OD cannot distinguish between living and dead cells, this estimate of cell density is only reliable for increasing or stable population sizes, making it unsuitable for quantifying negative rates (kill rates). Similarly, fluorescence is unsuitable for measuring population decline, as cell death does not inactivate the fluorescent proteins.

CFU assays estimate bacterial density by counting the colonies that grow on permissive agar media from plated samples. They remain the gold standard for measuring population size under both sub-MIC and super-MIC conditions and are widely used to quantify PD curves (e.g. Regoes et al., 2004; Foerster et al., 2016).

Luminescence assays measure the light emitted by bioluminescent bacterial cultures and can be used as a proxy to estimate changes in population size. Two main approaches for biological assays are: eukaryotic *luc* systems and the prokaryotic *lux* systems. The *luc* system, derived from eukaryotes such as fireflies, uses an ATP-dependent luciferase that oxidizes exogenous luciferin to emit light (Vellend et al., 1977). It was adapted for bacterial reporters by chromosomal integration in *Mycobacterium tuberculosis* (Jacobs et al., 1993) and later tested for quantifying antibiotic killing in *Streptococcus gordonii* (Loeliger et al., 2003). However, *luc*-based assays are limited by sensitivity to intracellular ATP, the need for addition of a costly substrate, and luciferin degradation, making continuous measurement in the same culture impractical.

By contrast, the *lux* operon of prokaryotes such as *Photorhabdus luminescens* encodes all components required to sustain the bioluminescence reaction (Engebrecht et al., 1985; Meighen, 1991). No external substrate is needed, so light production can be recorded continuously in the same culture, making the *lux* system better suited for high-throughput applications than the luc system. Accordingly, it has been widely used to record growth curves and quantify sub-MIC treatment effects (Kishony and Leibler, 2003; Yeh et al., 2006; Chait et al., 2007; Larsson et al., 2014; Kavčič et al., 2020; Angermayr et al., 2022).

While high-throughput OD and luminescence measurements at sub-MIC concentrations provide valuable insights into drug effects on growth rates, the super-MIC range is clinically more relevant. A comprehensive investigation of super-MIC population dynamics (e.g. PD of drug combinations or resistance mutations) using CFU remains impractical, as it is labor-intensive and inherently low-throughput.

Whether lux luminescence can be extended to super-MIC ranges remains unclear, as direct comparisons between CFU- and luminescence-based measurements are scarce and so far limited to only a few drugs (Salisbury et al., 1999; Beard et al., 2002; Alloush et al., 2003). Here, we evaluate the potential and limitations of bioluminescent bacteria as a high-throughput model system to quantify population-level net growth rates at the clinically most relevant concentrations (super-MIC). For this, we compare changes in light intensity with changes in CFU counts across 20 antimicrobials spanning 11 classes, including penicillins, cephalosporins, carbapenems, polymyxins, quinolones, rifamycins, tetracyclines, amphenicols, folate antagonists, fosfomycin, and antimicrobial peptides (AMPs). Luminescence- and CFU-based rates aligned for some antimicrobials (e.g. colistin, amoxicillin) but diverged for others (e.g. ciprofloxacin). In this work, we identify the conditions under which they align with the rate of change of population size and discuss the implications for studying antimicrobial effectiveness across sub-MIC and super-MIC ranges.

## Results

To investigate the validity of luminescence assays as a high-throughput measure for bacterial population size and its change across the entire range of antimicrobial concentrations, we compared this measure to the number of CFU. Specifically, we tested whether the rates of change in light intensity, $\psi_{I}$, and CFU, $\psi_{\mathrm{CFU}}$, agree for various drugs, and if not, we explored the reasons for any discrepancies.

We used a modified luminescence operon *luxCDABE* from *P. luminescens*. To minimize plasmid copy-number effects on the light emitted by a single cell (cell-specific luminosity), we excised the operon from the pCS-λ plasmid (Kishony and Leibler, 2003) and inserted it into the *Escherichia coli* chromosome.

### Light intensity is proportional to bacterial density

We first evaluated how the observed bioluminescent light intensity, I, which represents a fraction κ of the total light emitted by the culture, correlates with bacterial density. To this end, we prepared three replicate overnight cultures of bioluminescent *E. coli*, serially diluted them tenfold, and measured the light intensity for each dilution. We found that light intensity increased linearly with bacterial density for signals above approximately $20\;\mathrm{rlu}$ (Appendix 1—figure 1; $R^{2}=0.987$, n=20, $\rm p < 10^{-14}$), with a proportionality constant $m=0.006\frac{\mathrm{rlu}\,\mathrm{mL}}{\mathrm{CFU}}$. From this observation, we conclude that κ remains independent of bacterial density, indicating that, up to one-tenth of the stationary-phase density, high cell densities do not attenuate emitted light. Thus, provided we maintain the same luminescence plate-reader setup, we can assume κ to be constant for all subsequent analyses.

### Luminescence-based rates agree with CFU-based kill rates in 11 out of 22 antimicrobial assays

**Figure 1.**
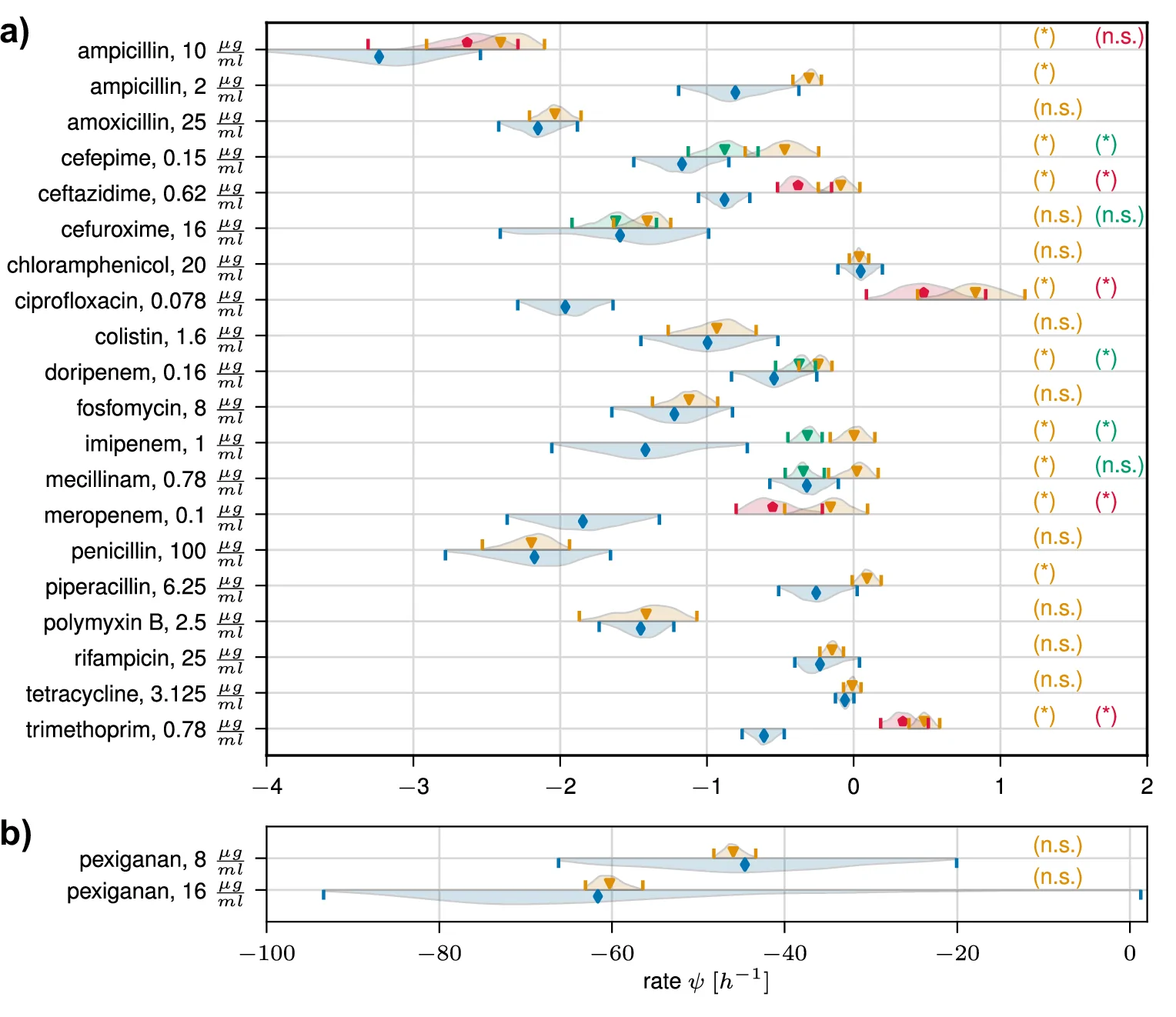
Comparison of colony-forming unit (CFU)-based and luminescence-based rates of change. For each drug, we generated 2000 bootstrapped datasets by resampling time-series CFU and light-intensity data with replacement and fitted an exponential function to each bootstrap replicate to obtain distributions of rates. Panel (**a**) shows these distributions for 20 drug-concentration assays across 19 antibiotics; panel (**b**) shows the antimicrobial peptide pexiganan at 8 μg/mL and 16 μg/mL. The distribution of $\psi_{\mathrm{CFU}}$ is shown in blue (lower half of each violin, diamond). The distribution of $\psi_{I}$ is shown in orange (upper half, triangle). Green distributions ($\psi_{I}^{*}$; triangle) represent luminescence-based rates calculated from data starting at the first peak onward. Red distributions show volume-adjusted luminescence rates ($\psi_{J}$; pentagon). Vertical lines mark the 95% confidence intervals. Asterisk (*) or letters (n.s.) indicate whether CFU-based rates differ significantly from the corresponding luminescence-based rate or not (see Materials and methods), with color coding matching the respective luminescence-based distribution. Wide confidence intervals for pexiganan reflect biphasic killing, steep curves, and noisy CFU data.

Given the linearity between light intensity and bacterial density shown above, we tested whether the rate of change of light intensity $\psi_{I}$ aligns with the rate of change of bacterial population size ($\psi_{B}$) under super-MIC antimicrobial concentrations. Since we cannot measure $\psi_{B}$ directly, we first compared $\psi_{I}$ to the CFU-based rate, $\psi_{\mathrm{CFU}}$, and then discussed their relation to $\psi_{B}$. We measured CFU and light intensity over time for 20 drugs (Appendix 1—table 1) using an automated liquid handler (Materials and methods) and estimated the distributions of the rates of change of CFU ($\psi_{\mathrm{CFU}}$) and light intensity ($\psi_{I}$) by bootstrap (Figure 1, Appendix 1—table 2). We classified the luminescence and CFU-based rate distributions as ‘not significantly different’ (n.s.) if each mean fell within the other’s 95% percentile and otherwise significantly different (*). All time-series data are presented as figure supplements to Figure 1. For amoxicillin, cefuroxime, chloramphenicol, colistin, fosfomycin, penicillin, pexiganan, polymyxin B, rifampicin, and tetracycline, $\psi_{I}$, and $\psi_{\rm CFU}$ did not differ significantly. However, we observed significant discrepancies for ampicillin, cefepime, ceftazidime, ciprofloxacin, doripenem, imipenem, mecillinam, meropenem, piperacillin, and trimethoprim.

**Figure 1—figure supplement 1.**
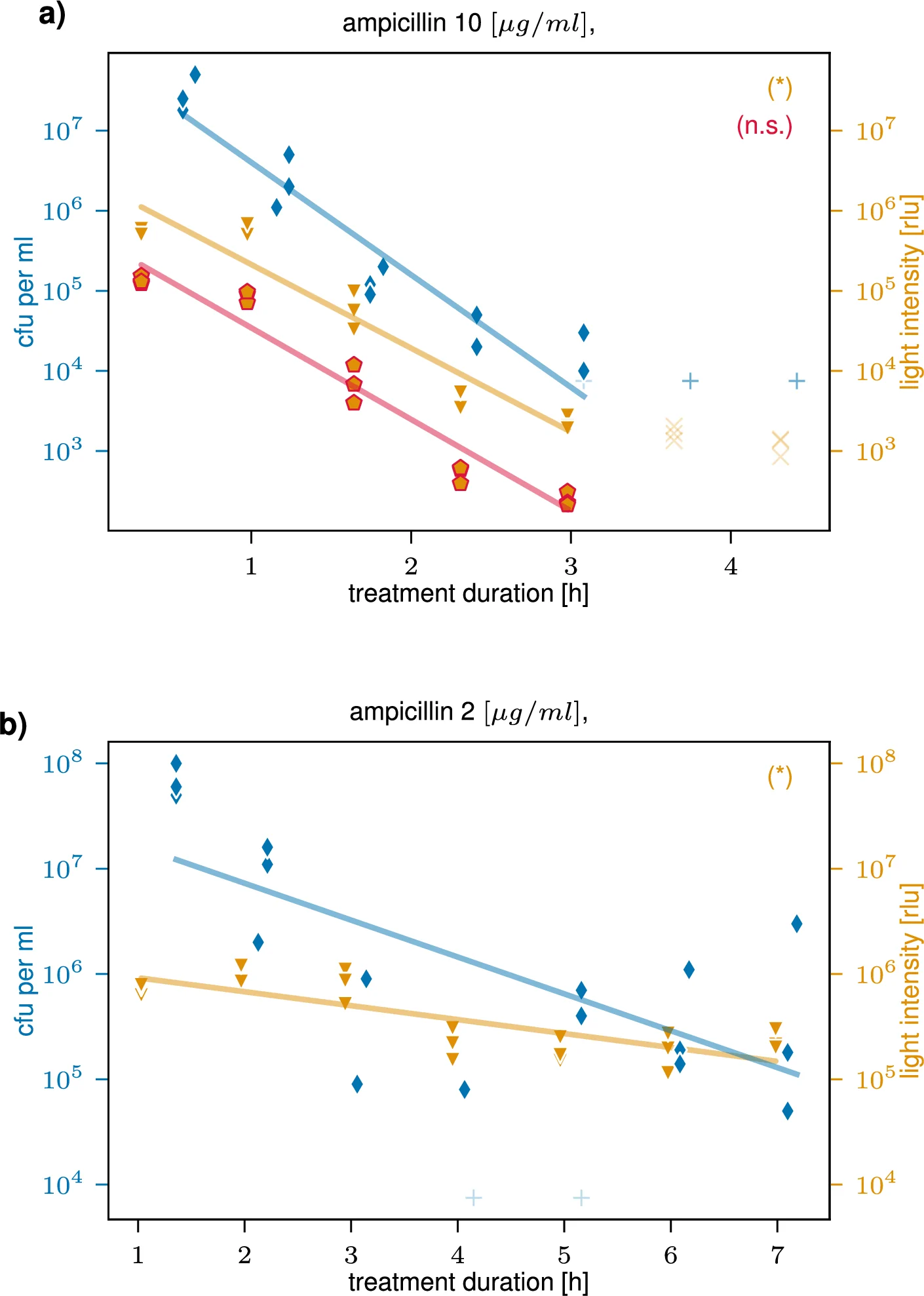
Ampicillin. The colony-forming unit (CFU) signal is shown as blue diamonds and the light intensity as orange triangles for data points above their respective detection limits (${10}^{4}\,\mathrm{C}\mathrm{F}\mathrm{U}\mathrm{/}\mathrm{m}\mathrm{L}$ for CFU and 20 rlu for luminescence). Lines represent log-linear fits to the corresponding signals. The morphology-corrected luminescence signal is shown as orange pentagons with red frames, and the corresponding rate fit indicated by a red line. ‘+’ indicates data points below detection limit or otherwise excluded (for CFU, symbolically plotted at ${10}^{4}\,\mathrm{C}\mathrm{F}\mathrm{U}\mathrm{/}\mathrm{m}\mathrm{L}$ to visualize missing data), and if a signal ends early (due to dropping below its detection limit), the corresponding data point of the other signal was cut to the same endpoint for a consistent comparison. These excluded data points are marked as ‘X’.

**Figure 1—figure supplement 2.**
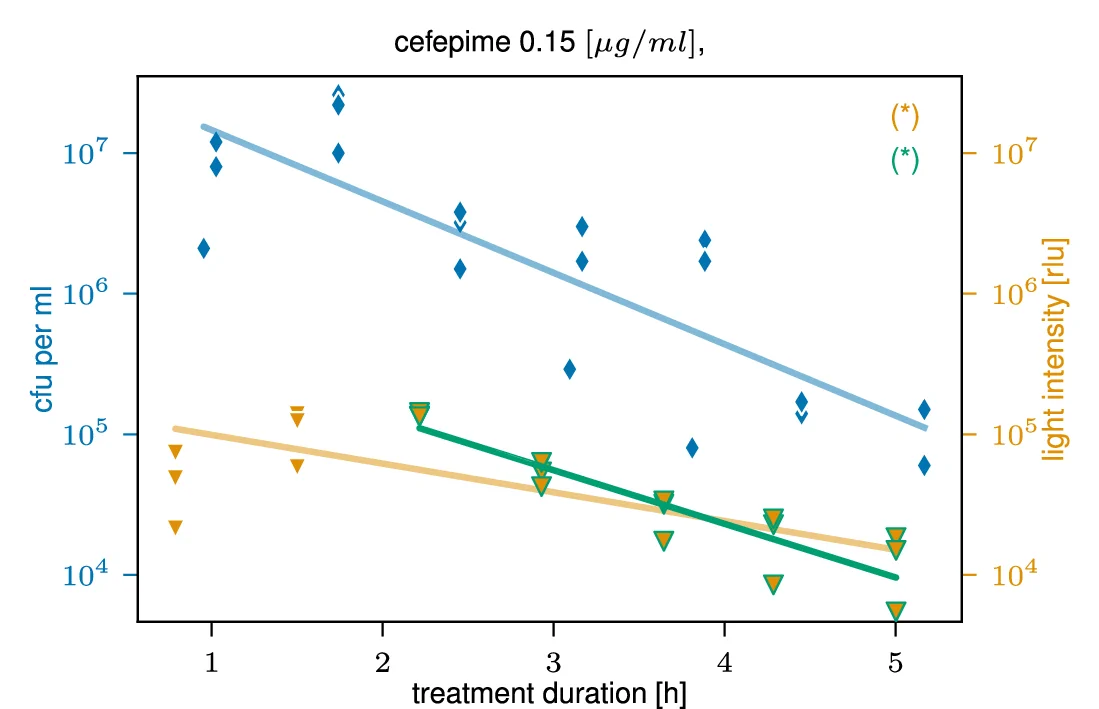
Cefepime. The colony-forming unit (CFU) signal is shown as blue diamonds and the light intensity as orange triangles for data points above their respective detection limits (${10}^{4}\,\mathrm{C}\mathrm{F}\mathrm{U}\mathrm{/}\mathrm{m}\mathrm{L}$ for CFU and 20 rlu for luminescence). Lines represent log-linear fits to the corresponding signals. Green-framed data points and corresponding green fit lines indicate analyses excluding early data points until the first peak.

**Figure 1—figure supplement 3.**
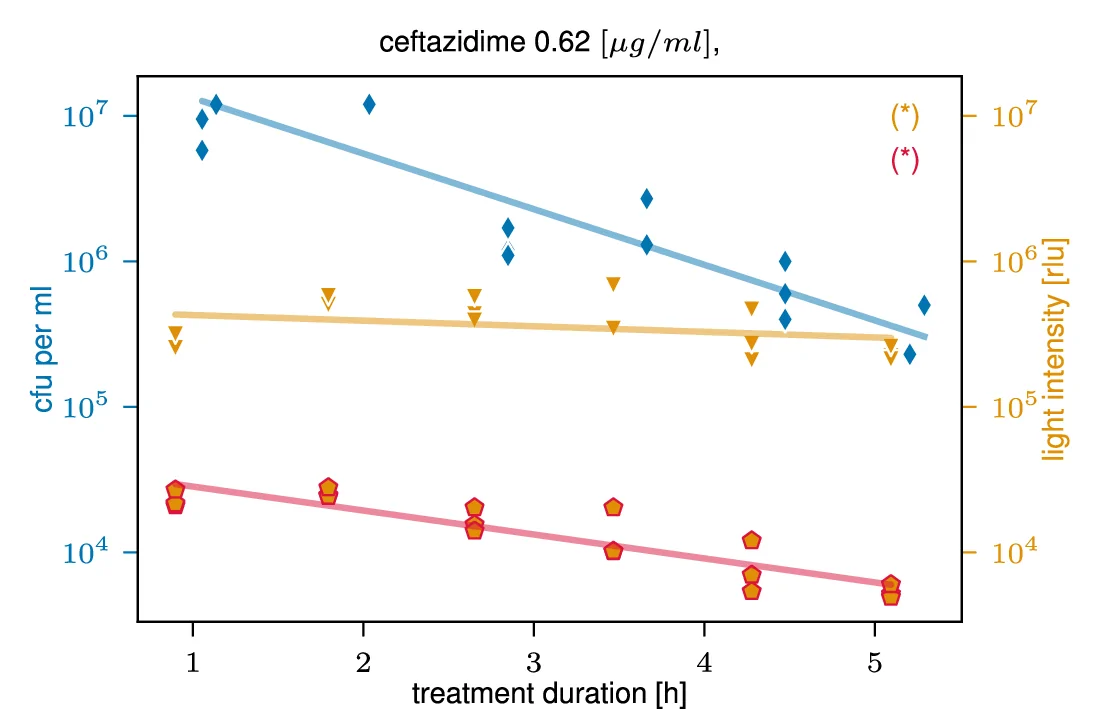
Ceftazidime. The colony-forming unit (CFU) signal is shown as blue diamonds and the light intensity as orange triangles for data points above their respective detection limits (${10}^{4}\,\mathrm{C}\mathrm{F}\mathrm{U}\mathrm{/}\mathrm{m}\mathrm{L}$ for CFU and 20 rlu for luminescence). Lines represent log-linear fits to the corresponding signals. The morphology-corrected luminescence signal is shown as orange pentagons with red frames, and the corresponding rate fit indicated by a red line.

**Figure 1—figure supplement 4.**
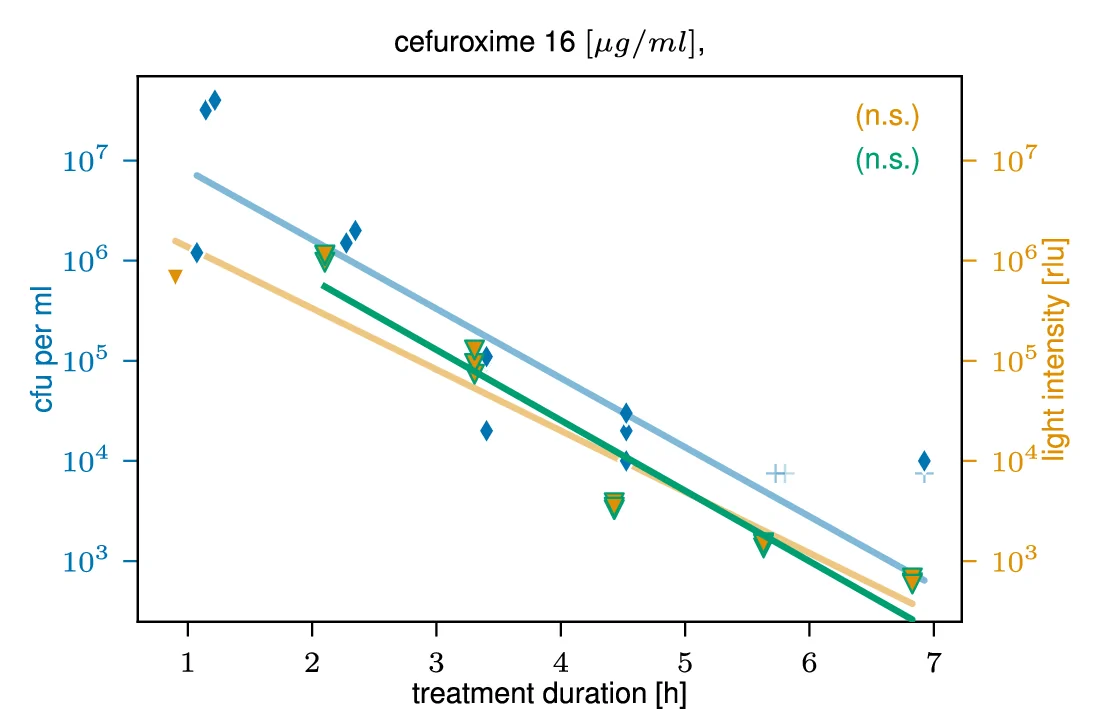
Cefuroxime. The colony-forming unit (CFU) signal is shown as blue diamonds and the light intensity as orange triangles for data points above their respective detection limits (${10}^{4}\,\mathrm{C}\mathrm{F}\mathrm{U}\mathrm{/}\mathrm{m}\mathrm{L}$ for CFU and 20 rlu for luminescence). Lines represent log-linear fits to the corresponding signals. ‘+’ indicates data points below detection limit or otherwise excluded (for CFU, symbolically plotted at ${10}^{4}\,\mathrm{C}\mathrm{F}\mathrm{U}\mathrm{/}\mathrm{m}\mathrm{L}$ to visualize missing data).

**Figure 1—figure supplement 5.**
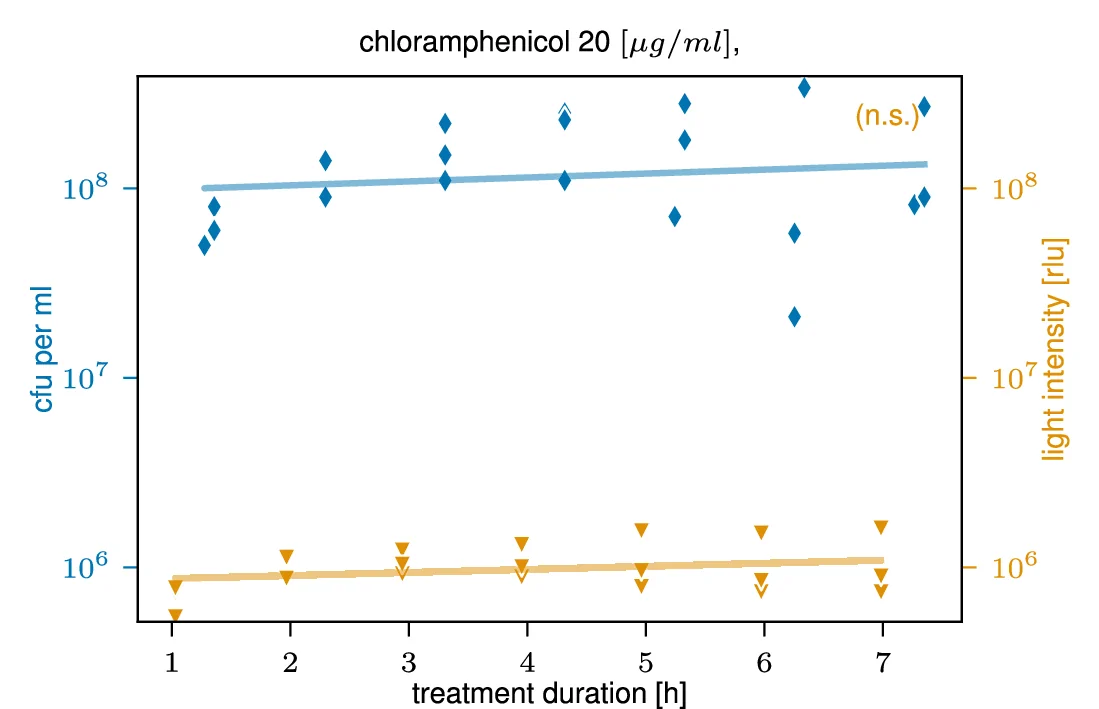
Chloramphenicol. The colony-forming unit (CFU) signal is shown as blue diamonds and the light intensity as orange triangles for data points above their respective detection limits (${10}^{4}\,\mathrm{C}\mathrm{F}\mathrm{U}\mathrm{/}\mathrm{m}\mathrm{L}$ for CFU and 20 rlu for luminescence). Lines represent log-linear fits to the corresponding signals.

**Figure 1—figure supplement 6.**
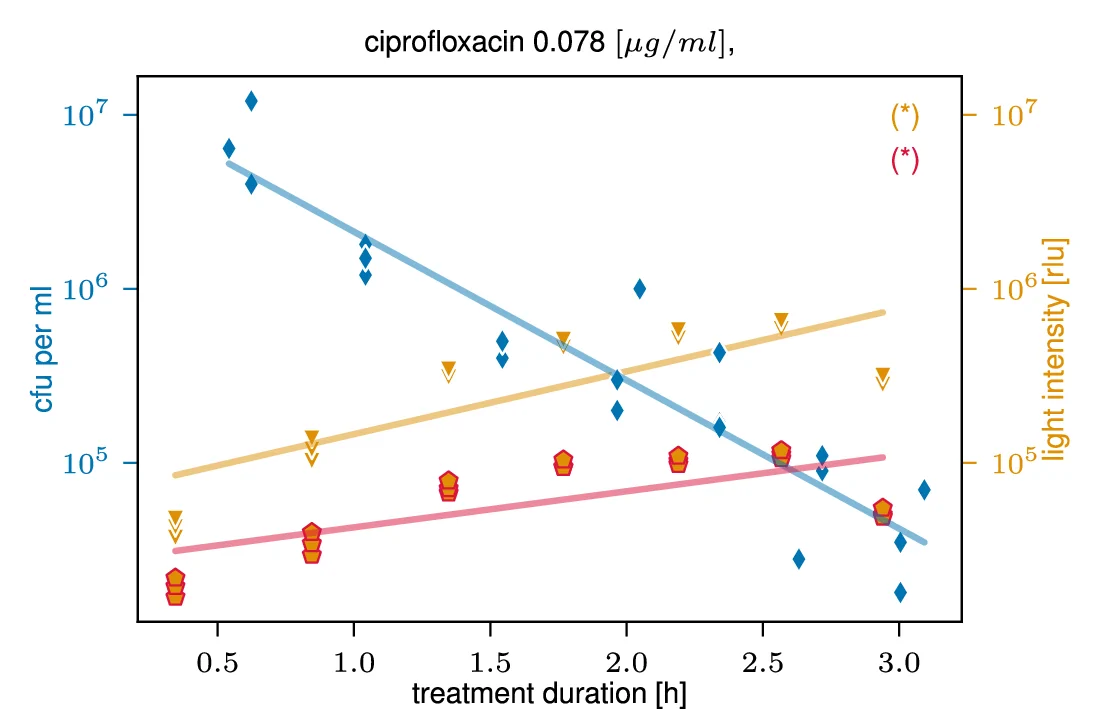
Ciprofloxacin. The colony-forming unit (CFU) signal is shown as blue diamonds and the light intensity as orange triangles for data points above their respective detection limits (${10}^{4}\,\mathrm{C}\mathrm{F}\mathrm{U}\mathrm{/}\mathrm{m}\mathrm{L}$ for CFU and 20 rlu for luminescence). Lines represent log-linear fits to the corresponding signals. The morphology-corrected luminescence signal is shown as orange pentagons with red frames, and the corresponding rate fit indicated by a red line.

**Figure 1—figure supplement 7.**
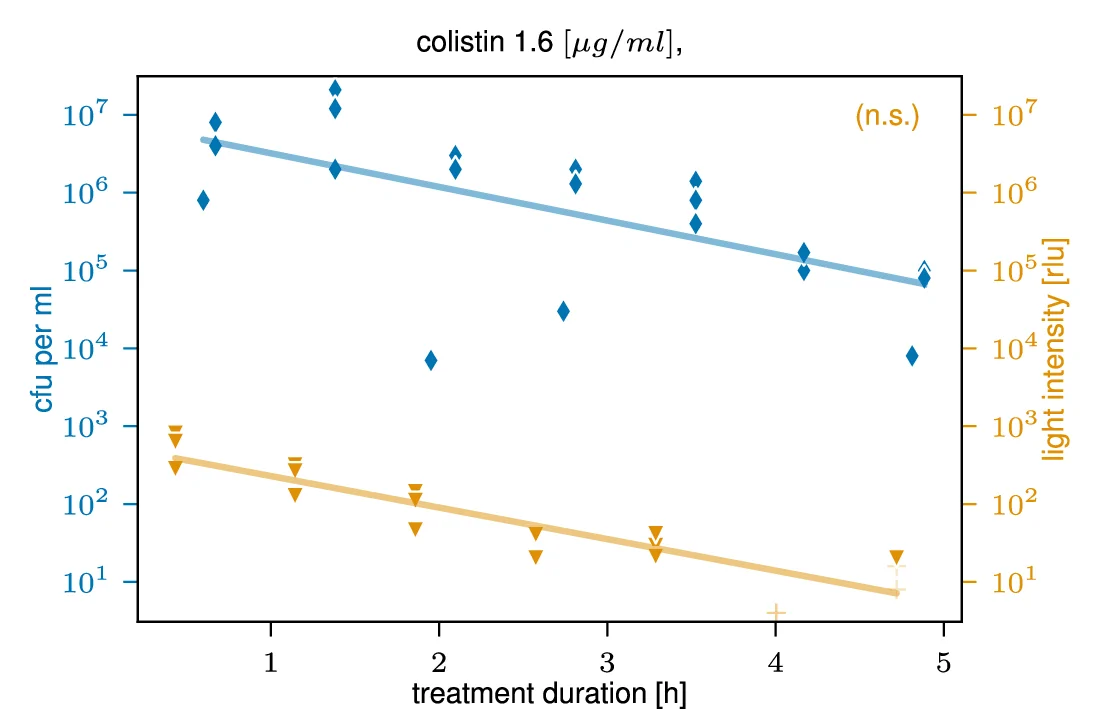
Colistin. The colony-forming unit (CFU) signal is shown as blue diamonds and the light intensity as orange triangles for data points above their respective detection limits (${10}^{4}\,\mathrm{C}\mathrm{F}\mathrm{U}\mathrm{/}\mathrm{m}\mathrm{L}$ for CFU and 20 rlu for luminescence). Lines represent log-linear fits to the corresponding signals. ‘+’ indicates data points below detection limit or otherwise excluded (for CFU, symbolically plotted at ${10}^{4}\,\mathrm{C}\mathrm{F}\mathrm{U}\mathrm{/}\mathrm{m}\mathrm{L}$ to visualize missing data).

**Figure 1—figure supplement 8.**
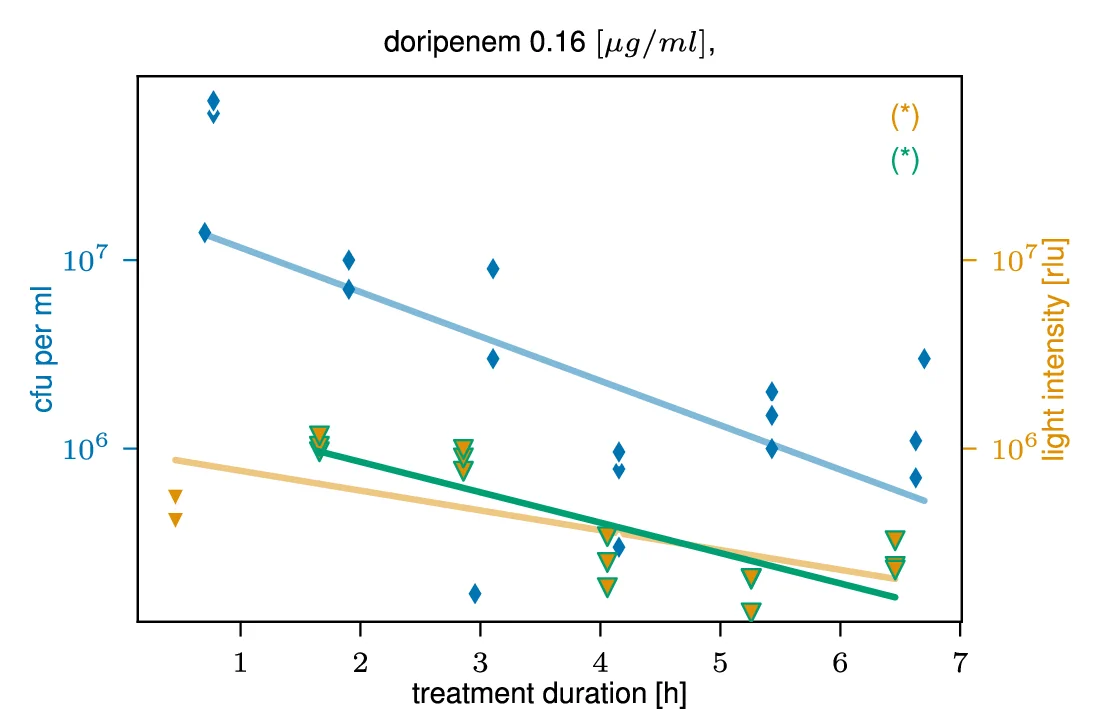
Doripenem. The colony-forming unit (CFU) signal is shown as blue diamonds and the light intensity as orange triangles for data points above their respective detection limits (${10}^{4}\,\mathrm{C}\mathrm{F}\mathrm{U}\mathrm{/}\mathrm{m}\mathrm{L}$ for CFU and 20 rlu for luminescence). Lines represent log-linear fits to the corresponding signals. Green-framed data points and corresponding green fit lines indicate analyses excluding early data points until the first peak.

**Figure 1—figure supplement 9.**
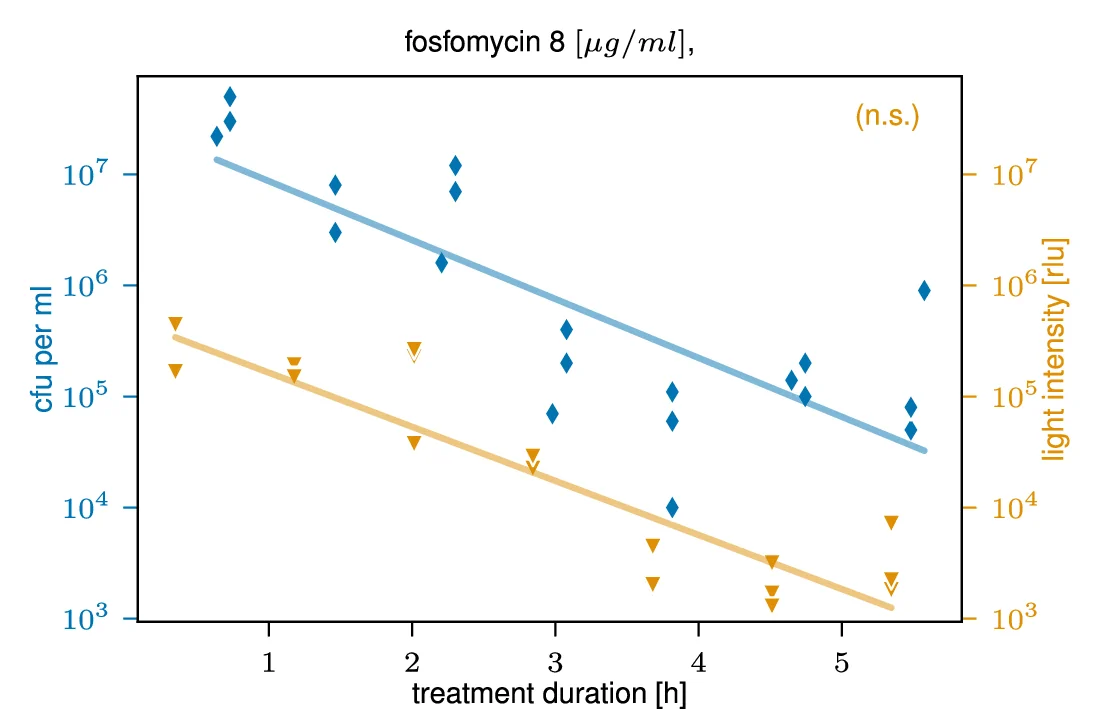
Fosfomycin. The colony-forming unit (CFU) signal is shown as blue diamonds and the light intensity as orange triangles for data points above their respective detection limits (${10}^{4}\,\mathrm{C}\mathrm{F}\mathrm{U}\mathrm{/}\mathrm{m}\mathrm{L}$ for CFU and 20 rlu for luminescence). Lines represent log-linear fits to the corresponding signals.

**Figure 1—figure supplement 10.**
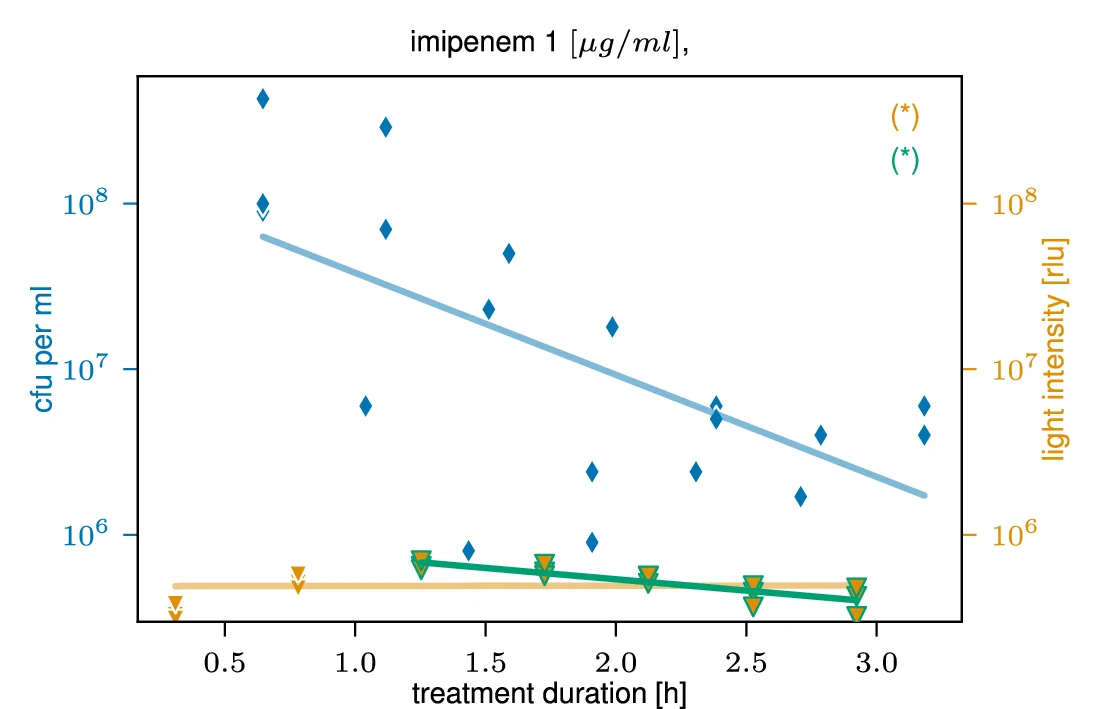
Imipenem. The colony-forming unit (CFU) signal is shown as blue diamonds and the light intensity as orange triangles for data points above their respective detection limits (${10}^{4}\,\mathrm{C}\mathrm{F}\mathrm{U}\mathrm{/}\mathrm{m}\mathrm{L}$ for CFU and 20 rlu for luminescence). Lines represent log-linear fits to the corresponding signals. ‘+’ indicates data points below detection limit or otherwise excluded (for CFU, symbolically plotted at ${10}^{4}\,\mathrm{C}\mathrm{F}\mathrm{U}\mathrm{/}\mathrm{m}\mathrm{L}$ to visualize missing data).

**Figure 1—figure supplement 11.**
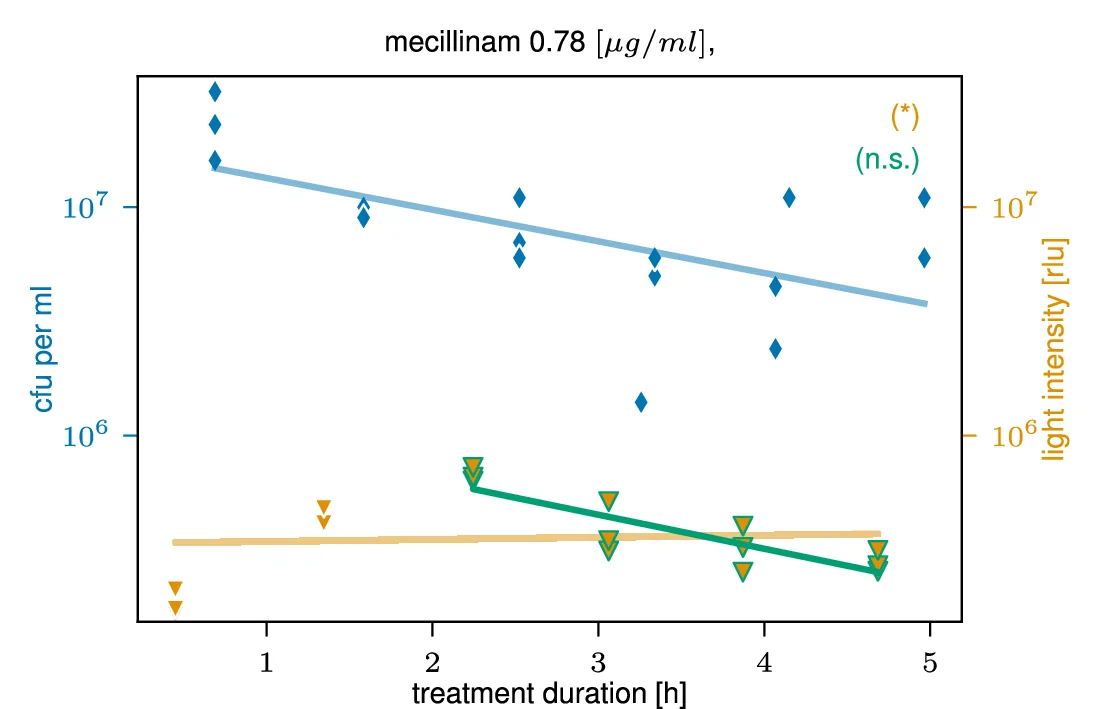
Mecillinam. The colony-forming unit (CFU) signal is shown as blue diamonds and the light intensity as orange triangles for data points above their respective detection limits (${10}^{4}\,\mathrm{C}\mathrm{F}\mathrm{U}\mathrm{/}\mathrm{m}\mathrm{L}$ for CFU and 20 rlu for luminescence). Lines represent log-linear fits to the corresponding signals.

**Figure 1—figure supplement 12.**
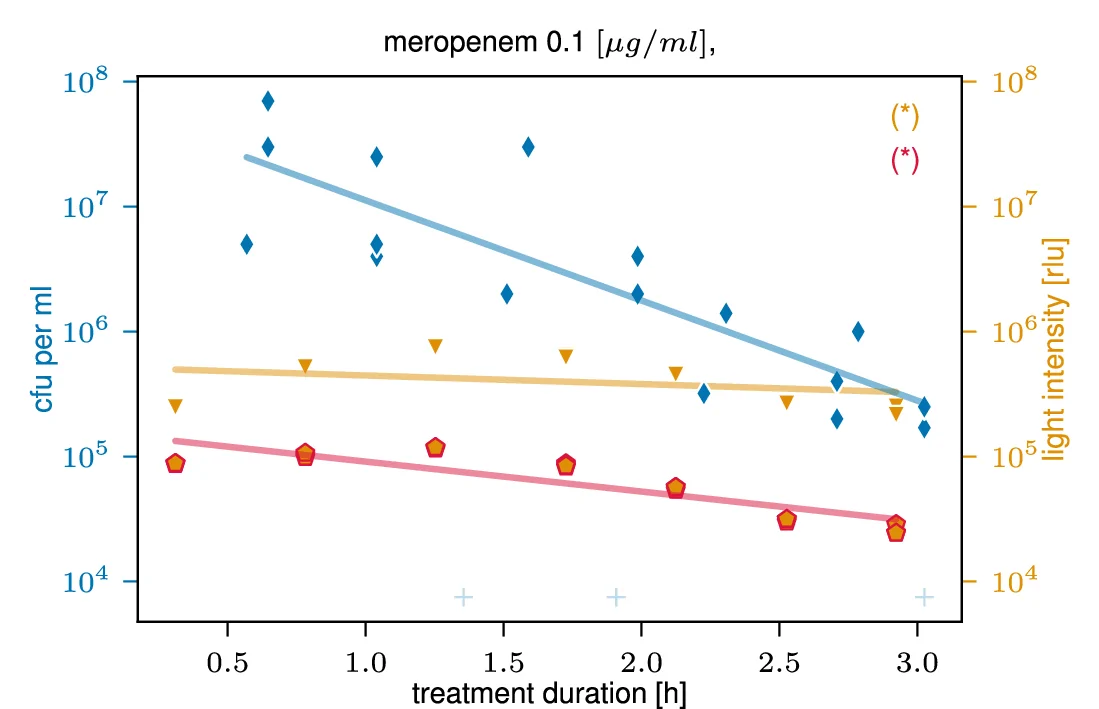
Meropenem. The colony-forming unit (CFU) signal is shown as blue diamonds and the light intensity as orange triangles for data points above their respective detection limits (${10}^{4}\,\mathrm{C}\mathrm{F}\mathrm{U}\mathrm{/}\mathrm{m}\mathrm{L}$ for CFU and 20 rlu for luminescence). Lines represent log-linear fits to the corresponding signals. The morphology-corrected luminescence signal is shown as orange pentagons with red frames, and the corresponding rate fit indicated by a red line. ‘+’ indicates data points below detection limit or otherwise excluded (for CFU, symbolically plotted at ${10}^{4}\,\mathrm{C}\mathrm{F}\mathrm{U}\mathrm{/}\mathrm{m}\mathrm{L}$ to visualize missing data).

**Figure 1—figure supplement 13.**
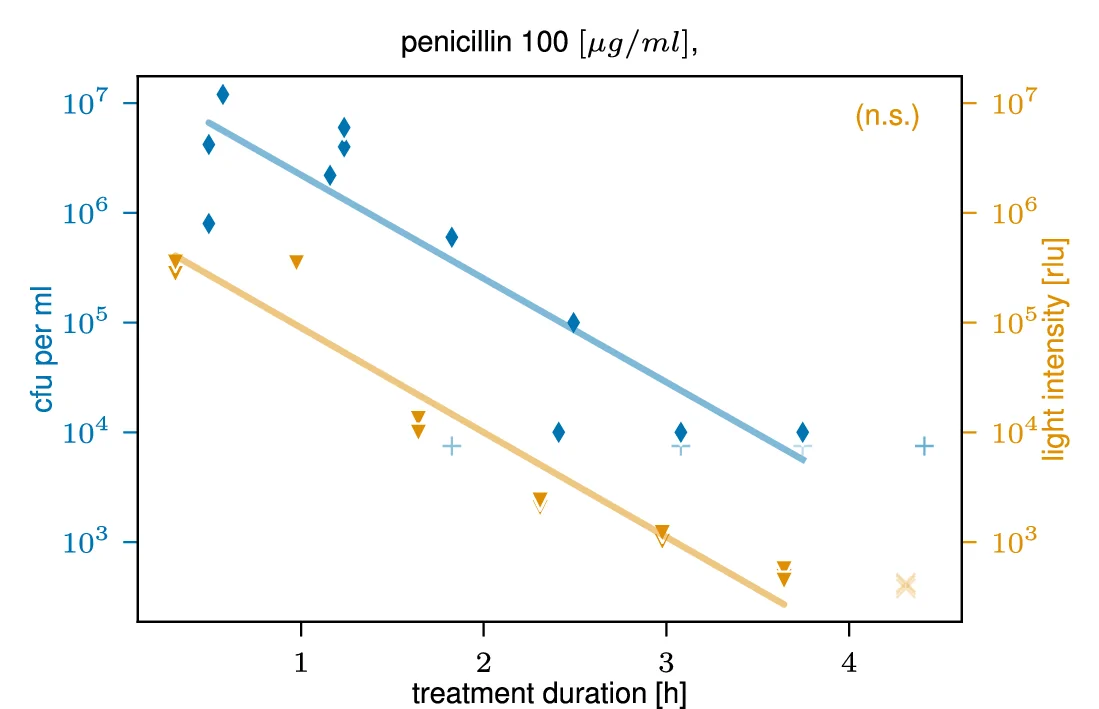
Penicillin. The colony-forming unit (CFU) signal is shown as blue diamonds and the light intensity as orange triangles for data points above their respective detection limits (${10}^{4}\,\mathrm{C}\mathrm{F}\mathrm{U}\mathrm{/}\mathrm{m}\mathrm{L}$ for CFU and 20 rlu for luminescence). Lines represent log-linear fits to the corresponding signals. ‘+’ indicates data points below detection limit or otherwise excluded (for CFU, symbolically plotted at ${10}^{4}\,\mathrm{C}\mathrm{F}\mathrm{U}\mathrm{/}\mathrm{m}\mathrm{L}$ to visualize missing data), and if a signal ends early (due to dropping below its detection limit), the corresponding data point of the other signal was cut to the same endpoint for a consistent comparison. These excluded data points are marked as ‘X’.

**Figure 1—figure supplement 14.**
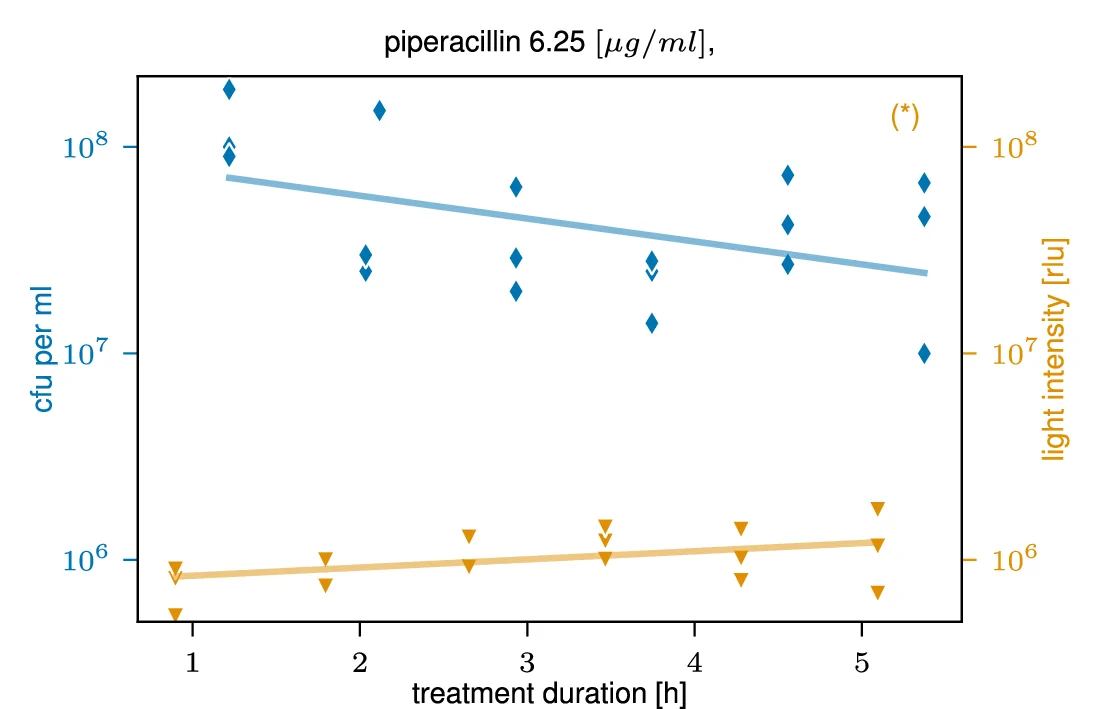
Piperacillin. The colony-forming unit (CFU) signal is shown as blue diamonds and the light intensity as orange triangles for data points above their respective detection limits (${10}^{4}\,\mathrm{C}\mathrm{F}\mathrm{U}\mathrm{/}\mathrm{m}\mathrm{L}$ for CFU and 20 rlu for luminescence). Lines represent log-linear fits to the corresponding signals.

**Figure 1—figure supplement 15.**
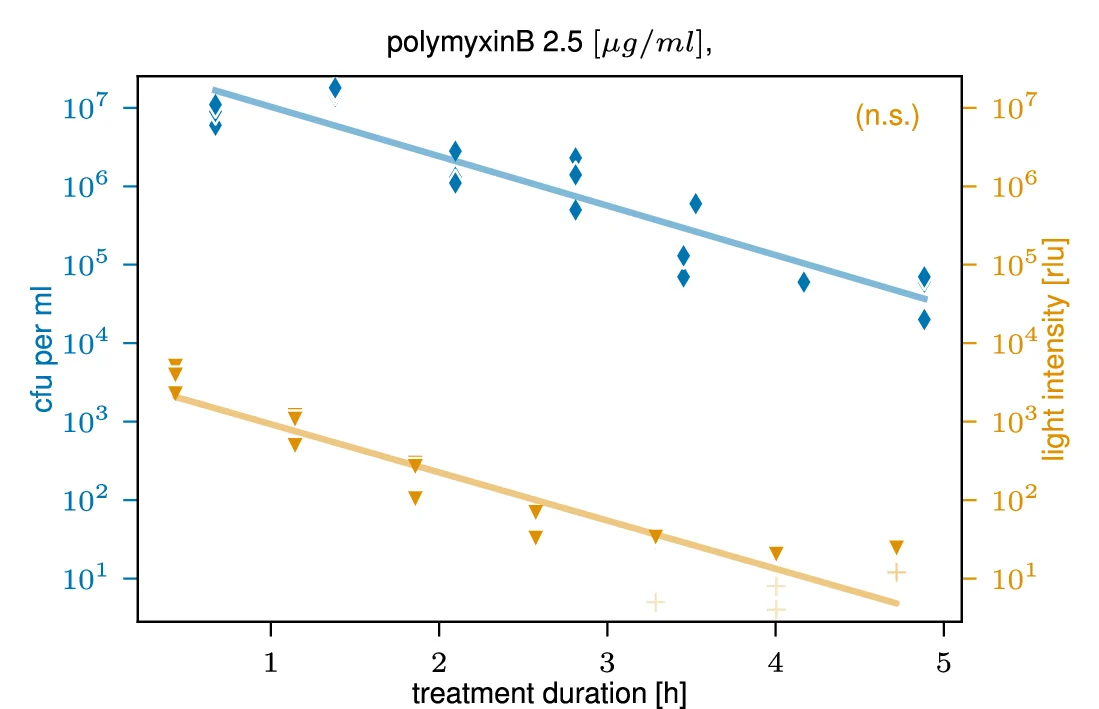
Polymyxin B. The colony-forming unit (CFU) signal is shown as blue diamonds and the light intensity as orange triangles for data points above their respective detection limits (${10}^{4}\,\mathrm{C}\mathrm{F}\mathrm{U}\mathrm{/}\mathrm{m}\mathrm{L}$ for CFU and 20 rlu for luminescence). Lines represent log-linear fits to the corresponding signals. ‘+’ indicates data points below detection limit or otherwise excluded (for CFU, symbolically plotted at ${10}^{4}\,\mathrm{C}\mathrm{F}\mathrm{U}\mathrm{/}\mathrm{m}\mathrm{L}$ to visualize missing data).

**Figure 1—figure supplement 16.**
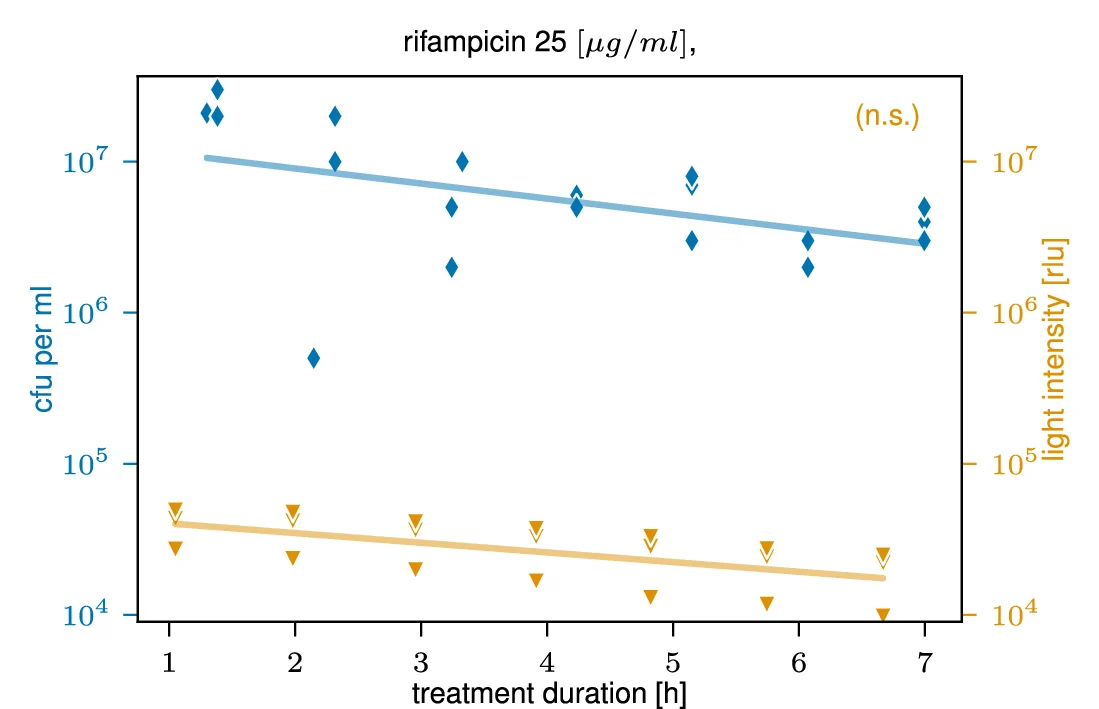
Rifampicin (25μg/mL). The colony-forming unit (CFU) signal is shown as blue diamonds and the light intensity as orange triangles for data points above their respective detection limits (${10}^{4}\,\mathrm{C}\mathrm{F}\mathrm{U}\mathrm{/}\mathrm{m}\mathrm{L}$ for CFU and 20 rlu for luminescence). Lines represent log-linear fits to the corresponding signals.

**Figure 1—figure supplement 17.**
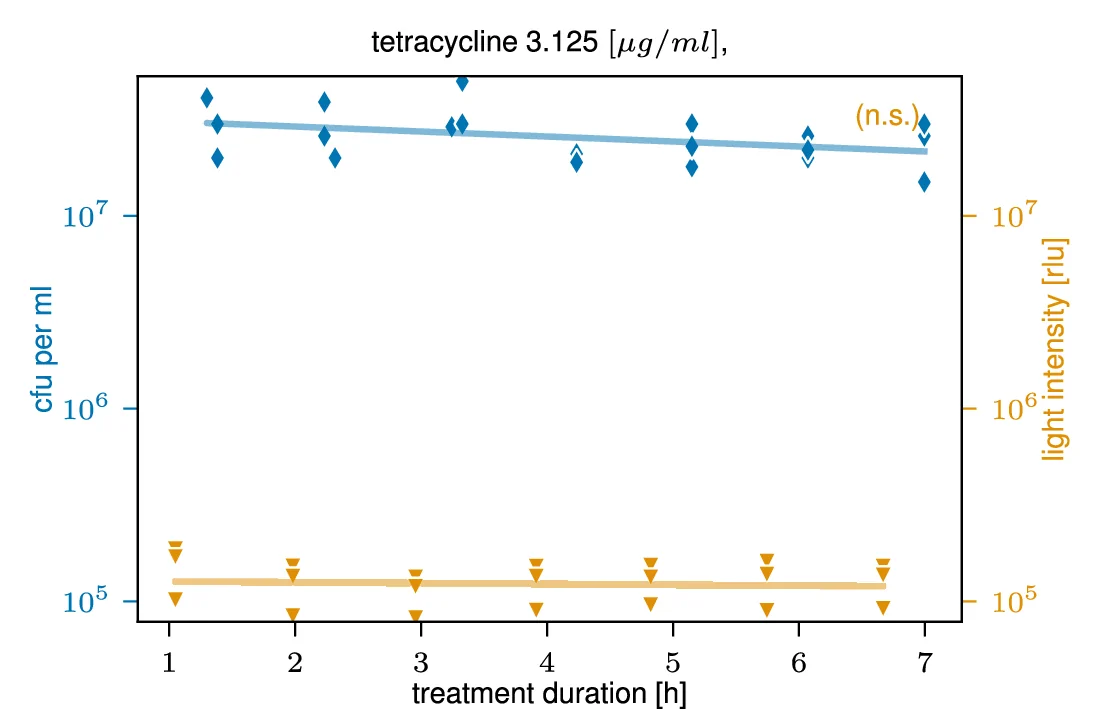
Tetracycline. The colony-forming unit (CFU) signal is shown as blue diamonds and the light intensity as orange triangles for data points above their respective detection limits (${10}^{4}\,\mathrm{C}\mathrm{F}\mathrm{U}\mathrm{/}\mathrm{m}\mathrm{L}$ for CFU and 20 rlu for luminescence). Lines represent log-linear fits to the corresponding signals.

**Figure 1—figure supplement 18.**
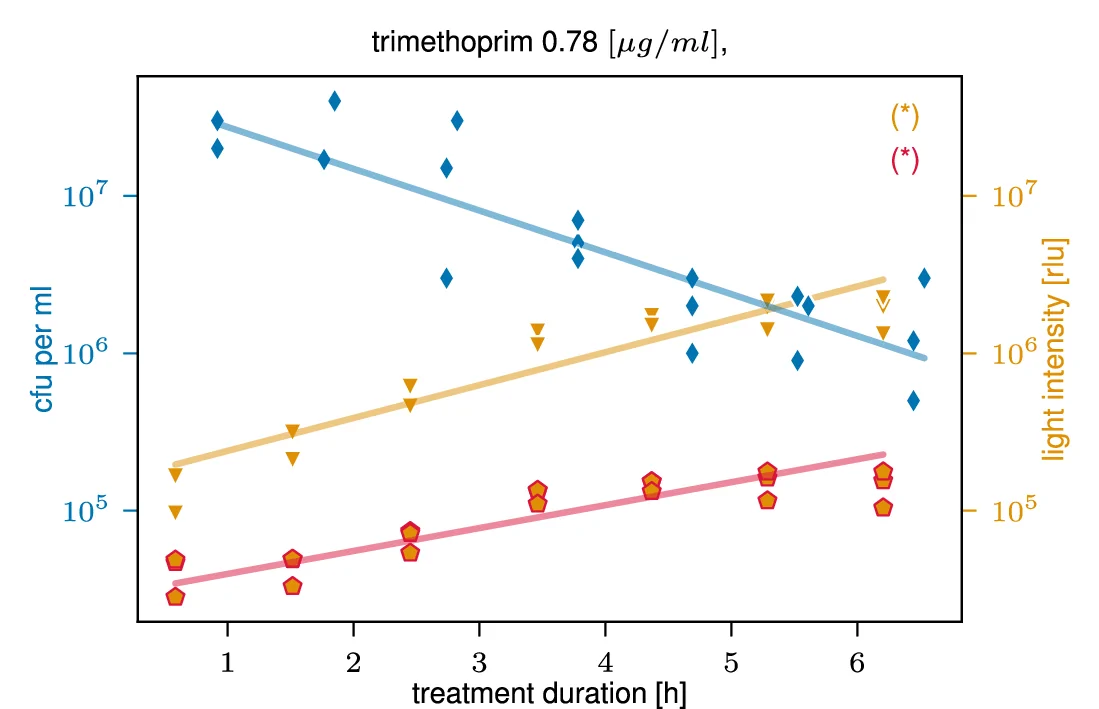
Trimethoprim. The colony-forming unit (CFU) signal is shown as blue diamonds and the light intensity as orange triangles for data points above their respective detection limits (${10}^{4}\,\mathrm{C}\mathrm{F}\mathrm{U}\mathrm{/}\mathrm{m}\mathrm{L}$ for CFU and 20 rlu for luminescence). Lines represent log-linear fits to the corresponding signals. The morphology-corrected luminescence signal is shown as orange pentagons with red frames, and the corresponding rate fit indicated by a red line.

**Figure 1—figure supplement 19.**
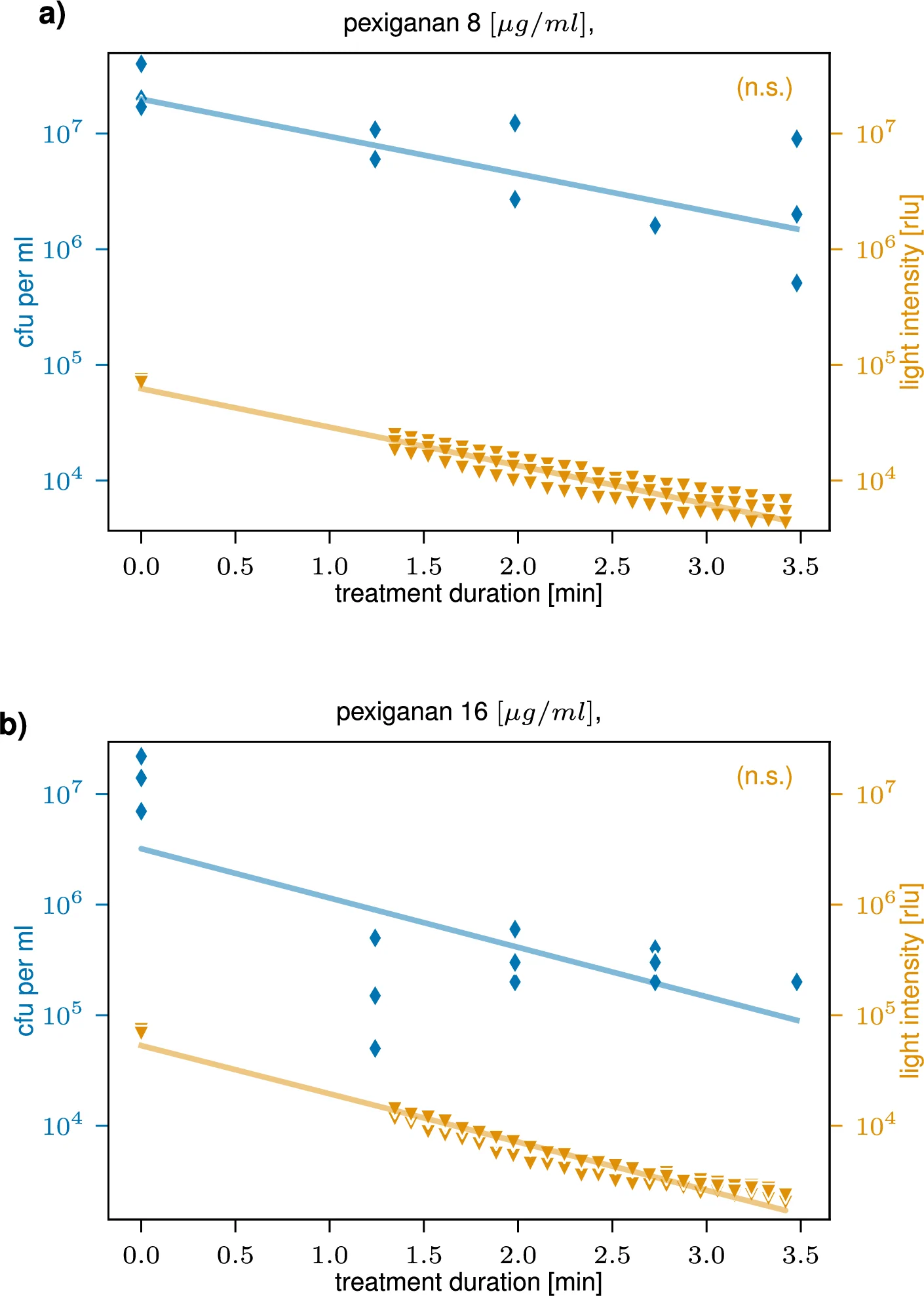
Comparison of colony-forming unit (CFU) and luminescence kill curve signals for pexiganan at (**a**) 8 μg/mL and (**b**) 16 μg/mL. The first data points at $t_{0}$ represent the pretreatment CFU and light intensity values.

For all cases where significant discrepancies were observed, the light intensity declined more slowly than the CFU signal. In the following, we investigate potential reasons why the light signal may decline more slowly and the CFU signal more rapidly than the ‘true’ $\psi_{B}$.

### No support for SOS-driven increase in luminescence promoter activity

To explain the observed discrepancies between CFU- and luminescence-based rates, we tested whether the SOS response might upregulate lux expression, increasing the cell-specific luminosity. To this end, we exposed the strains to UV light to induce the SOS response. Specifically, we alternated between measuring light intensity and optical density, and exposing cultures to UV (Appendix 1).

UV treatment impaired bacterial growth significantly, yet the OD-normalized light intensity (∝ cell-specific luminosity) of UV-treated cells was lower than that of untreated controls (Appendix 1—figure 2d; t-test, $\rm p=3\cdot 10^{-5}$ for the last time point). This implies that SOS induction did not upregulate lux expression, as cells under SOS had lower light output per cell than controls. While we cannot exclude the possibility that a non-UV-induced SOS response enhances cell-specific luminosity, our findings suggest that promoter upregulation is unlikely to explain why luminescence-based rates exceed CFU-based rates.

### Filamentation aligns with divergence between CFU- and luminescence-based rates

**Figure 2.**
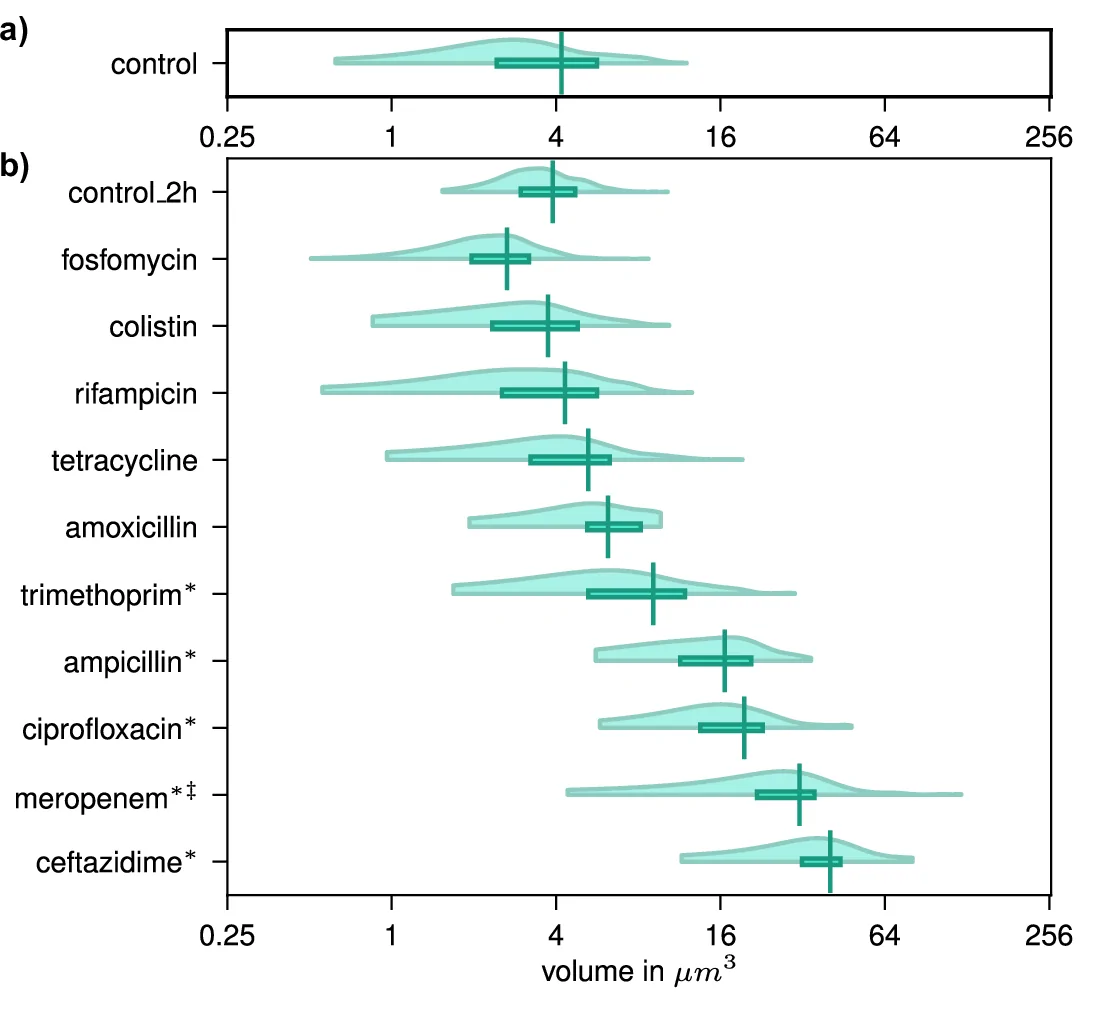
Density distributions (2.5–97.5% percentile range) of pooled cell volumes acquired by microscopy imaging, shown (**a**) before and (**b**) after 2 hr of antibiotic treatment. Boxes indicate the 25–75% interquartile range, and vertical bars mark the mean. Significance (*) was assessed by bootstrapping cell volumes 200 times with replacement for each replicate (image) and treatment, pooling the bootstrapped volumes by treatment, and comparing the resulting 95% confidence interval of the mean to that of the untreated control (control_2h). ‡ Carbapenems tend to deform cells into a lemon-like shape (Figure 2—figure supplement 10), resulting in poor fitting quality since our algorithm assumes a cylindrical geometry.

Antibiotic pressure is known to impair septation and induce bacterial filamentation. Our second hypothesis was that larger, filamented cells might emit more light per cell, thereby driving the divergence between CFU- and luminescence-based rates. We explored this for a subset of the antibiotics tested by imaging bacteria before and after 2 hr of antibiotic treatment (microscopy images are shown as supplements to Figure 2; see Materials and methods). From these images, we measured bacterial length and width (Figure 2—figure supplement 1) and calculated the cell volumes (Figure 2, Appendix 1).

**Figure 2—figure supplement 1.**
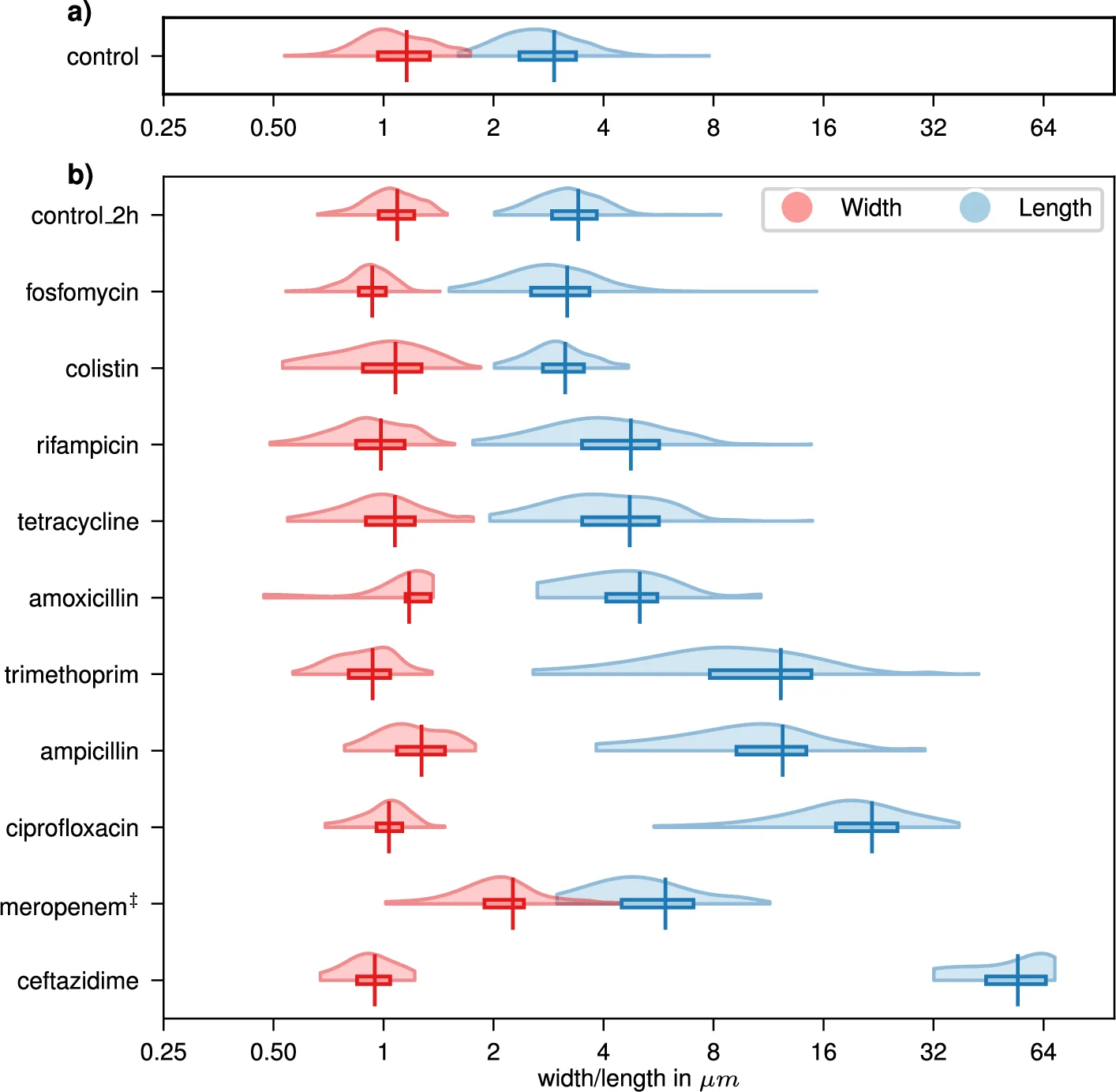
Pooled density distributions (95% prediction intervals) of cell widths (red) and lengths (blue), obtained from microscopy images (**a**) before and (**b**) after 2 hr of antibiotic treatment. Boxes indicate the interquartile range (Q1–Q3), and the mean is marked by (|). ‡ Poor fit quality for meropenem-treated cells, which adopt a lemon-like shape (Figure 2—figure supplement 10), due to our algorithm assuming cylindrical geometry.

**Figure 2—figure supplement 2.**
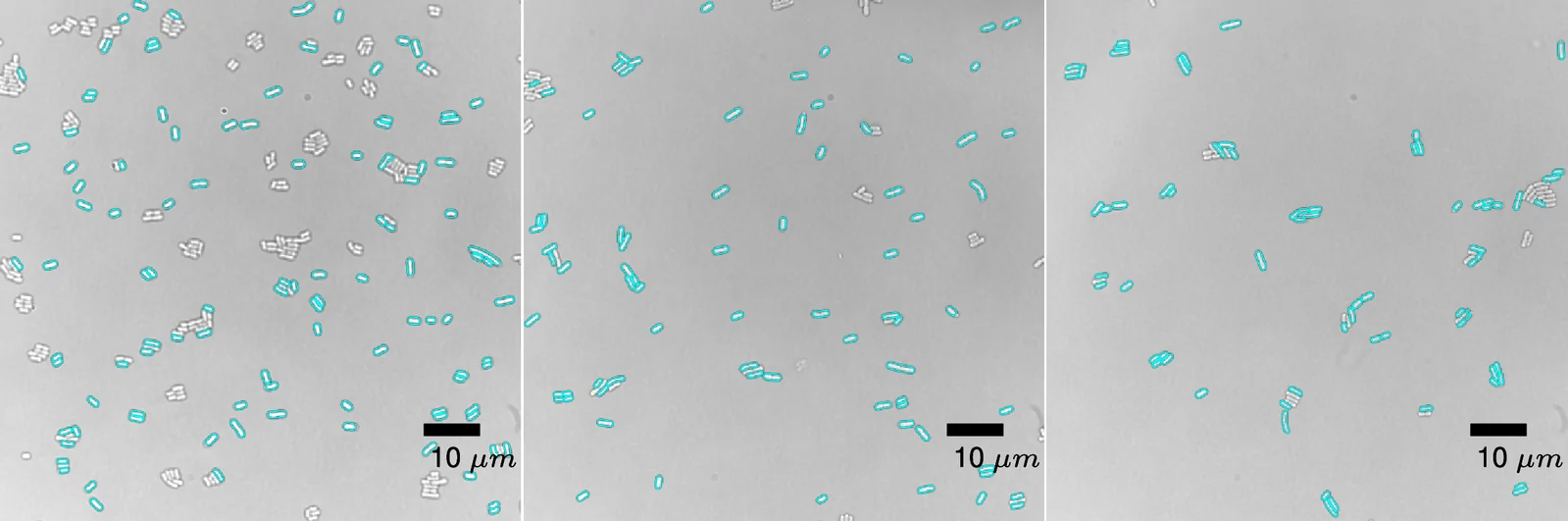
Microscopy images of control (before treatment) samples. Each image represents a different replicate. We plotted the green channel of the recorded images in grayscale. The red channel, capturing propidium iodide activity, is overlaid in red. Bacterial shapes detected by the algorithm are outlined in cyan.

**Figure 2—figure supplement 3.**
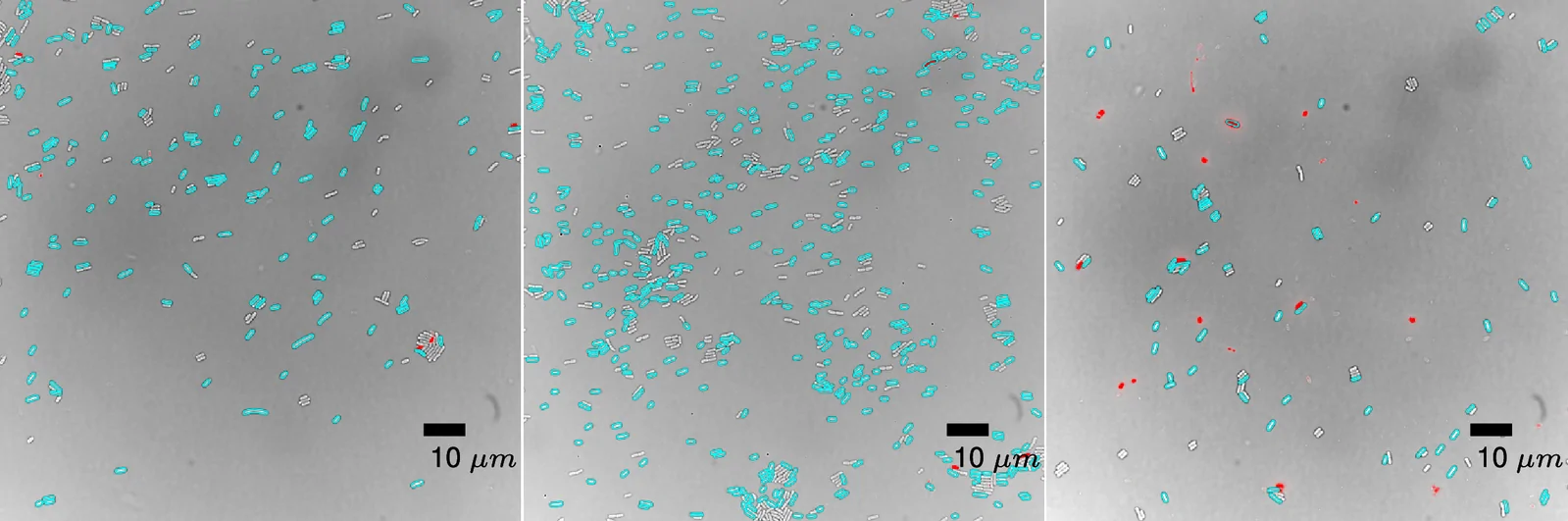
Microscopy images of control (after 2 hr) samples. Each image represents a different replicate. We plotted the green channel of the recorded images in grayscale. The red channel, capturing propidium iodide activity, is overlaid in red. Bacterial shapes detected by the algorithm are outlined in cyan.

**Figure 2—figure supplement 4.**
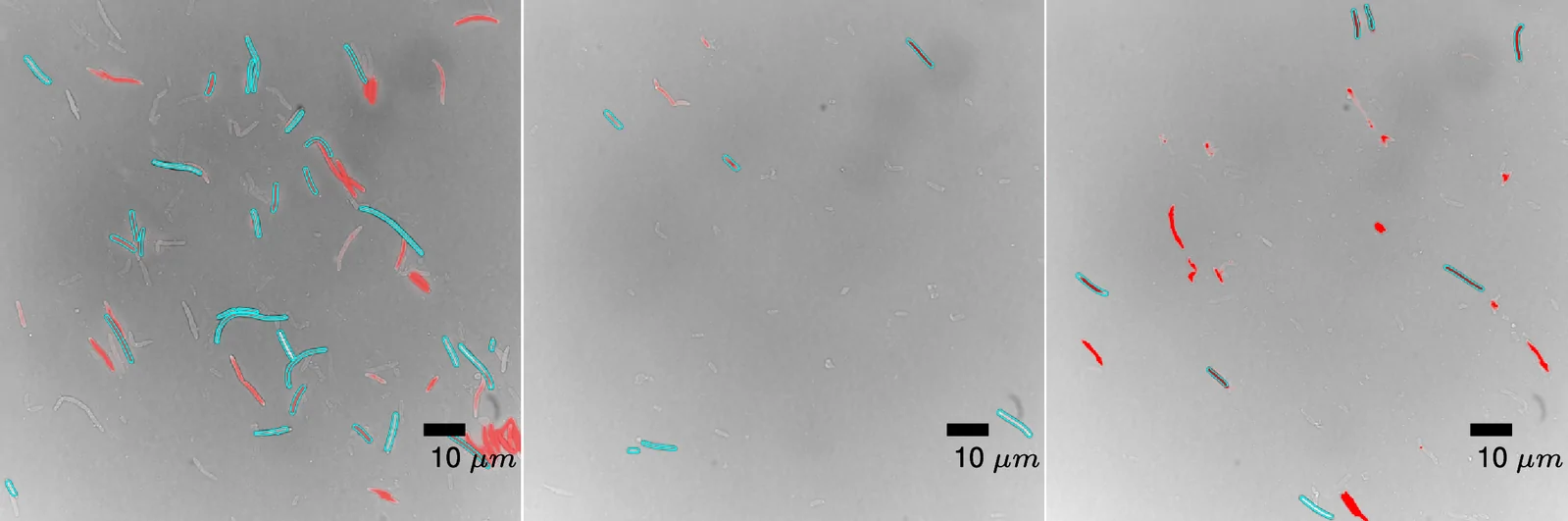
Microscopy images of cells treated with ampicillin. Each image represents a different replicate. We plotted the green channel of the recorded images in grayscale. The red channel, capturing propidium iodide activity, is overlaid in red. Bacterial shapes detected by the algorithm are outlined in cyan.

**Figure 2—figure supplement 5.**
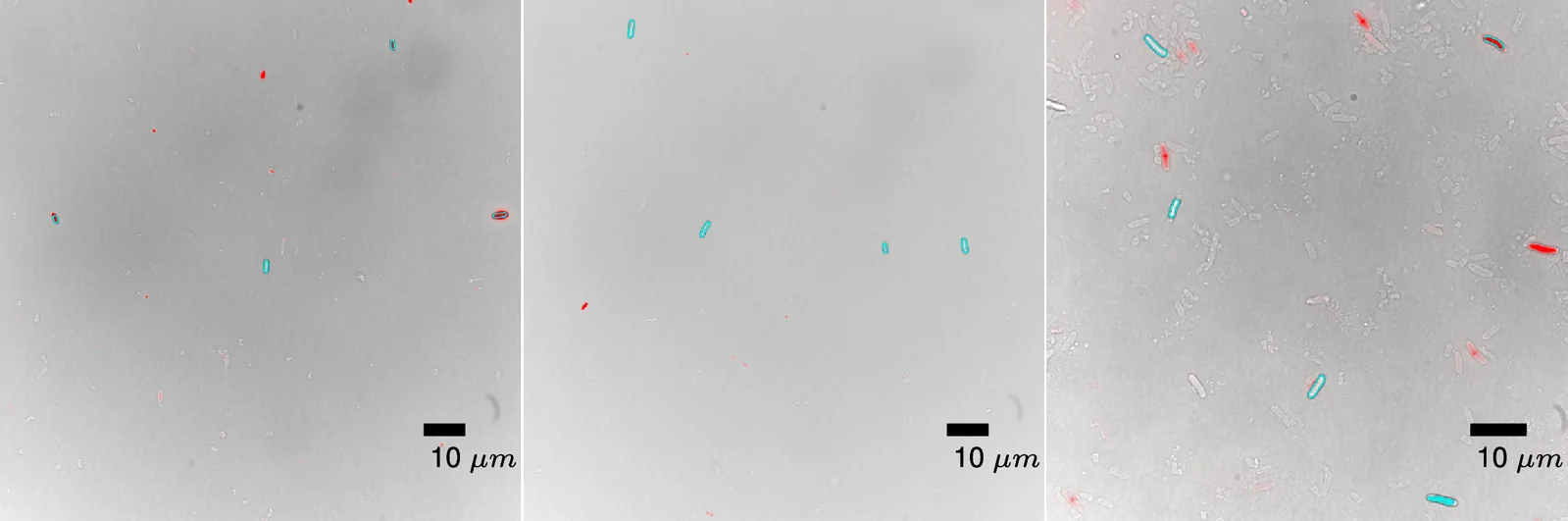
Microscopy images of cells treated with amoxicillin. Each image represents a different replicate. We plotted the green channel of the recorded images in grayscale. The red channel, capturing propidium iodide activity, is overlaid in red. Bacterial shapes detected by the algorithm are outlined in cyan.

**Figure 2—figure supplement 6.**
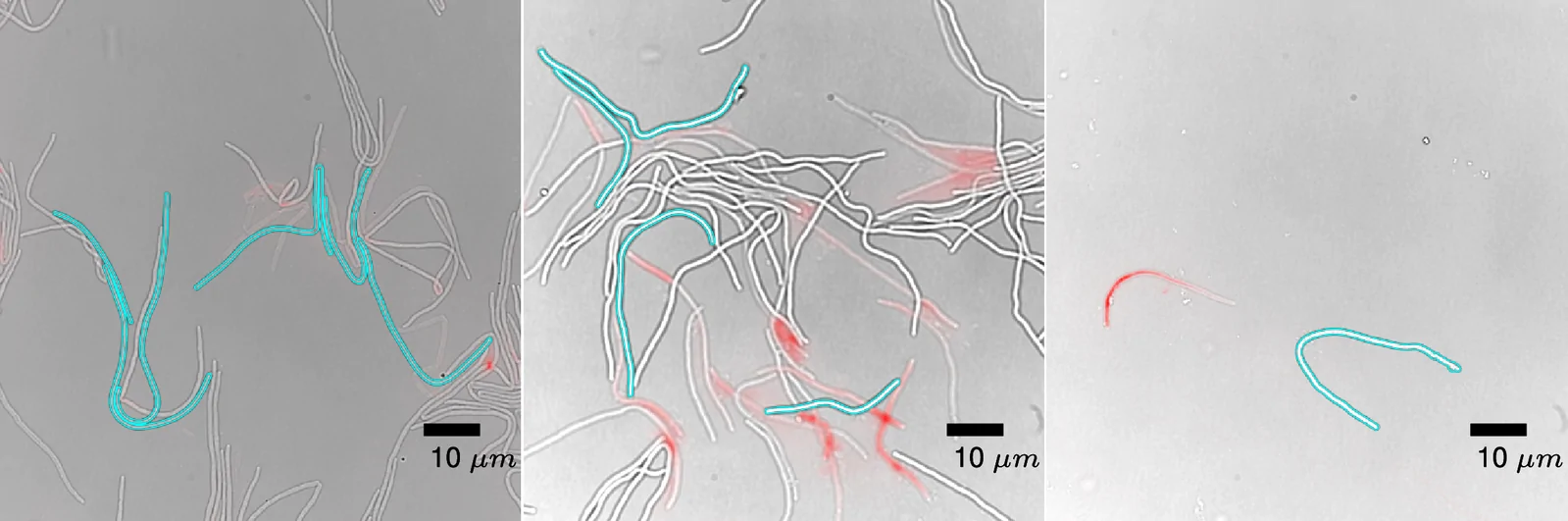
Microscopy images of cells treated with ceftazidime. Each image represents a different replicate. We plotted the green channel of the recorded images in grayscale. The red channel, capturing propidium iodide activity, is overlaid in red. Bacterial shapes detected by the algorithm are outlined in cyan.

**Figure 2—figure supplement 7.**
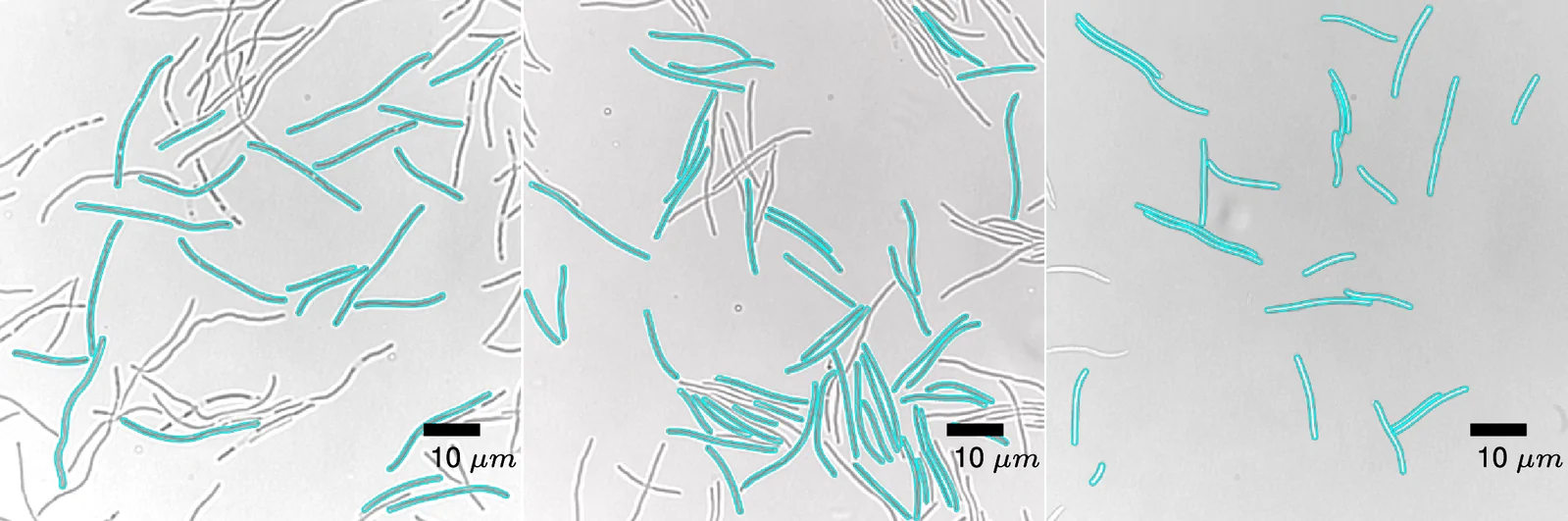
Microscopy images of cells treated with ciprofloxacin. Each image represents a different replicate. The white gaps can indicate the start of cell division (cells are not fixed). We plotted the green channel of the recorded images in grayscale. The red channel, capturing propidium iodide activity, is overlaid in red. Bacterial shapes detected by the algorithm are outlined in cyan.

**Figure 2—figure supplement 8.**
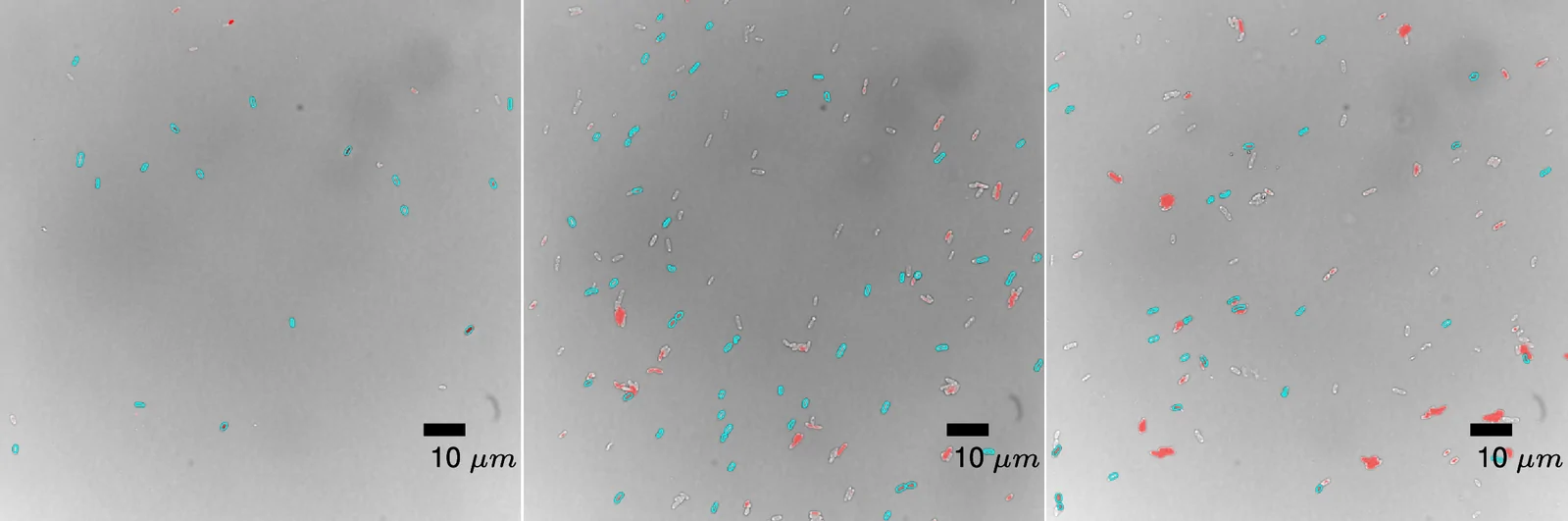
Microscopy images of cells treated with colistin. Each image represents a different replicate. We plotted the green channel of the recorded images in grayscale. The red channel, capturing propidium iodide activity, is overlaid in red. Bacterial shapes detected by the algorithm are outlined in cyan.

**Figure 2—figure supplement 9.**
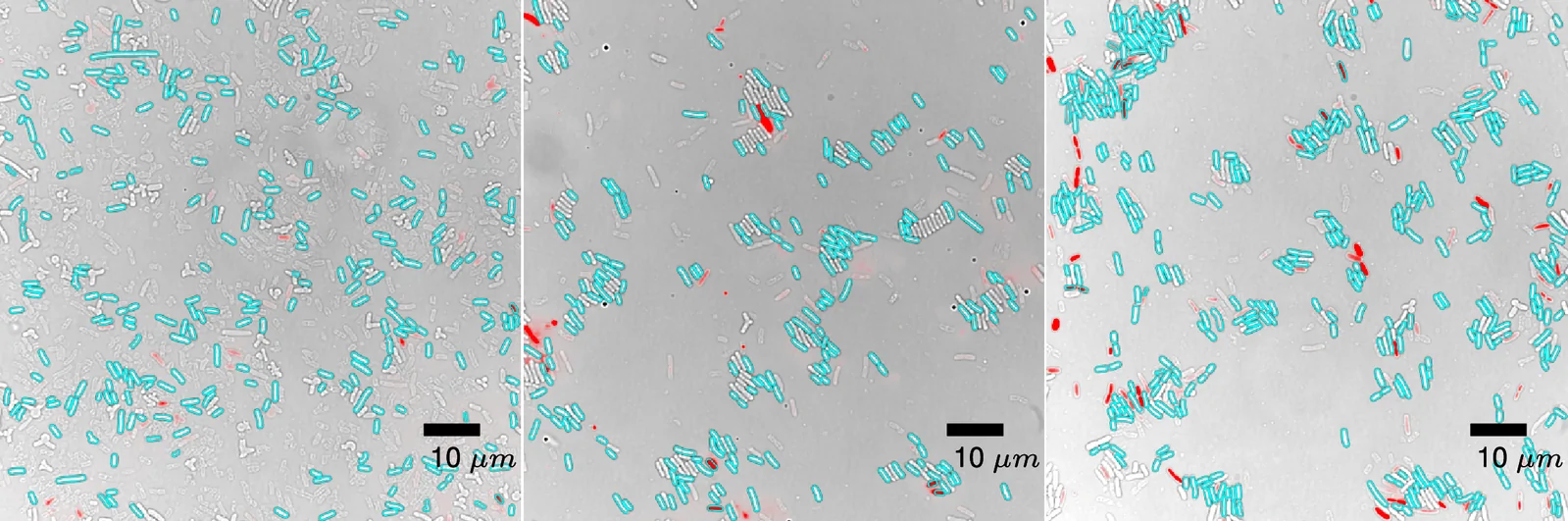
Microscopy images of cells treated with fosfomycin. Each image represents a different replicate. We plotted the green channel of the recorded images in grayscale. The red channel, capturing propidium iodide activity, is overlaid in red. Bacterial shapes detected by the algorithm are outlined in cyan.

**Figure 2—figure supplement 10.**
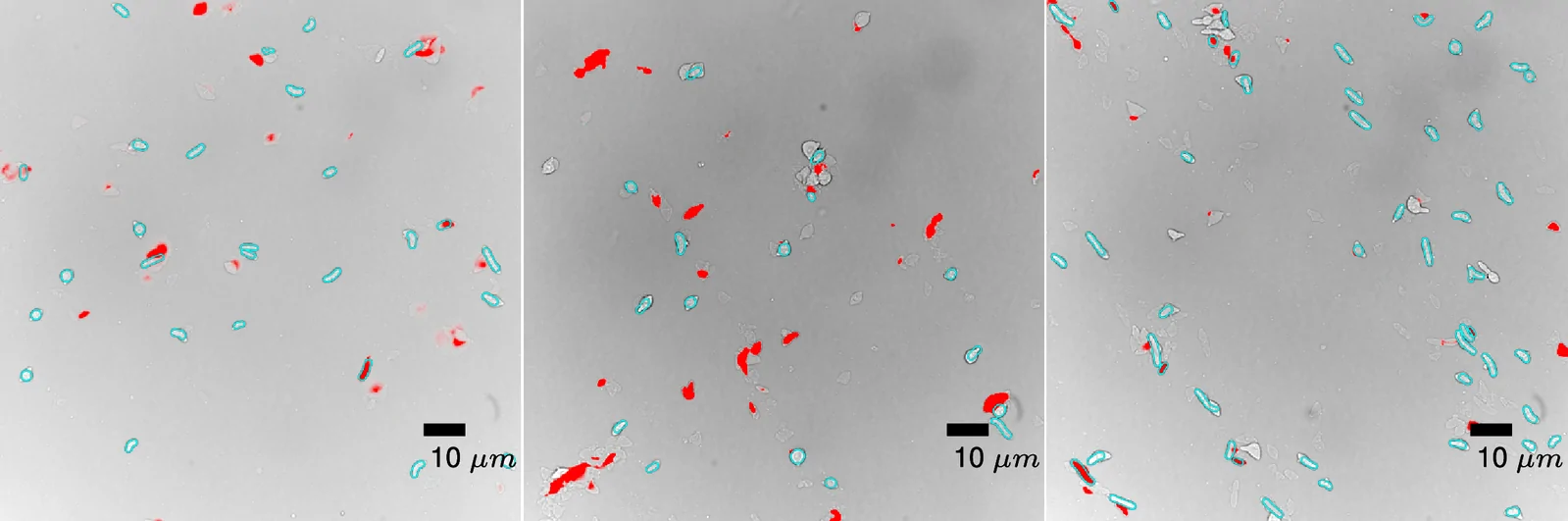
Microscopy images of cells treated with meropenem. Each image represents a different replicate. We plotted the green channel of the recorded images in grayscale. The red channel, capturing propidium iodide activity, is overlaid in red. Bacterial shapes detected by the algorithm are outlined in cyan.

**Figure 2—figure supplement 11.**
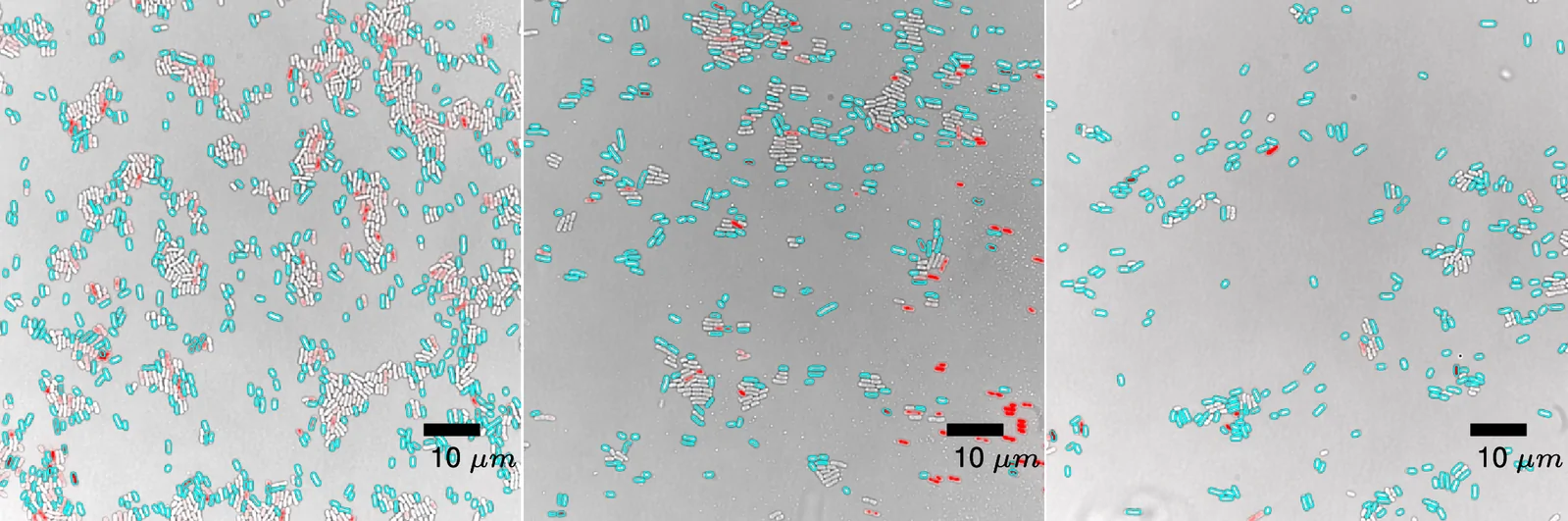
Microscopy images of cells treated with pexiganan. Each image represents a different replicate. We plotted the green channel of the recorded images in grayscale. The red channel, capturing propidium iodide activity, is overlaid in red. Bacterial shapes detected by the algorithm are outlined in cyan.

**Figure 2—figure supplement 12.**
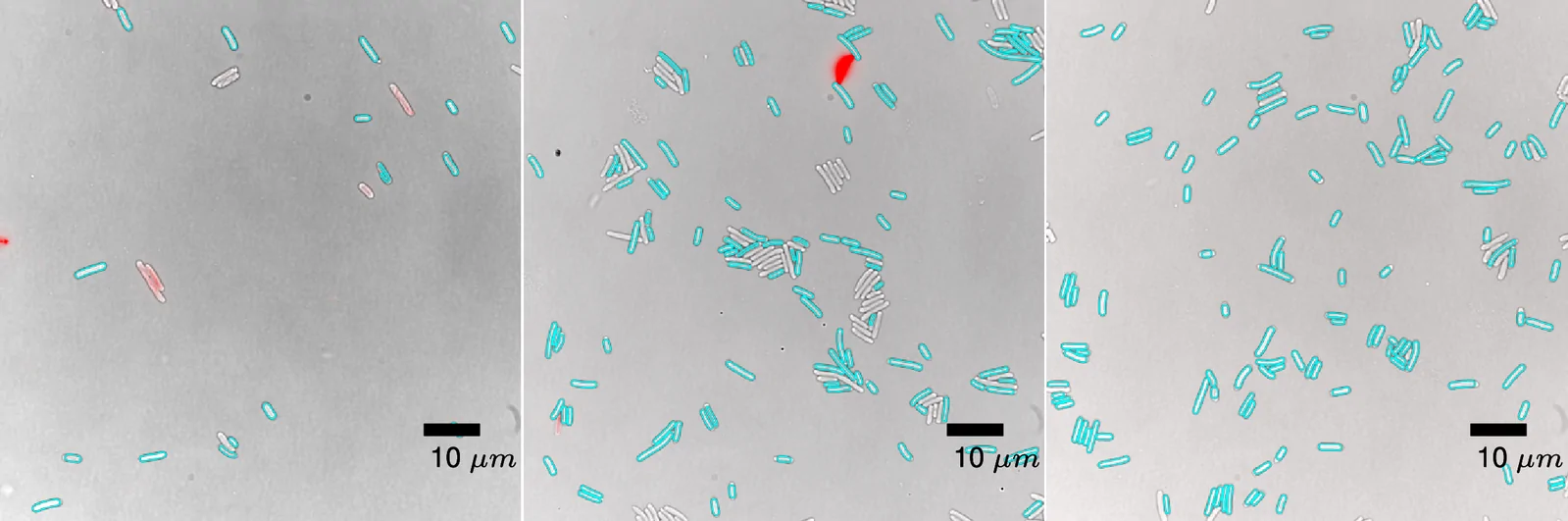
Microscopy images of cells treated with rifampicin. Each image represents a different replicate. We plotted the green channel of the recorded images in grayscale. The red channel, capturing propidium iodide activity, is overlaid in red. Bacterial shapes detected by the algorithm are outlined in cyan.

**Figure 2—figure supplement 13.**
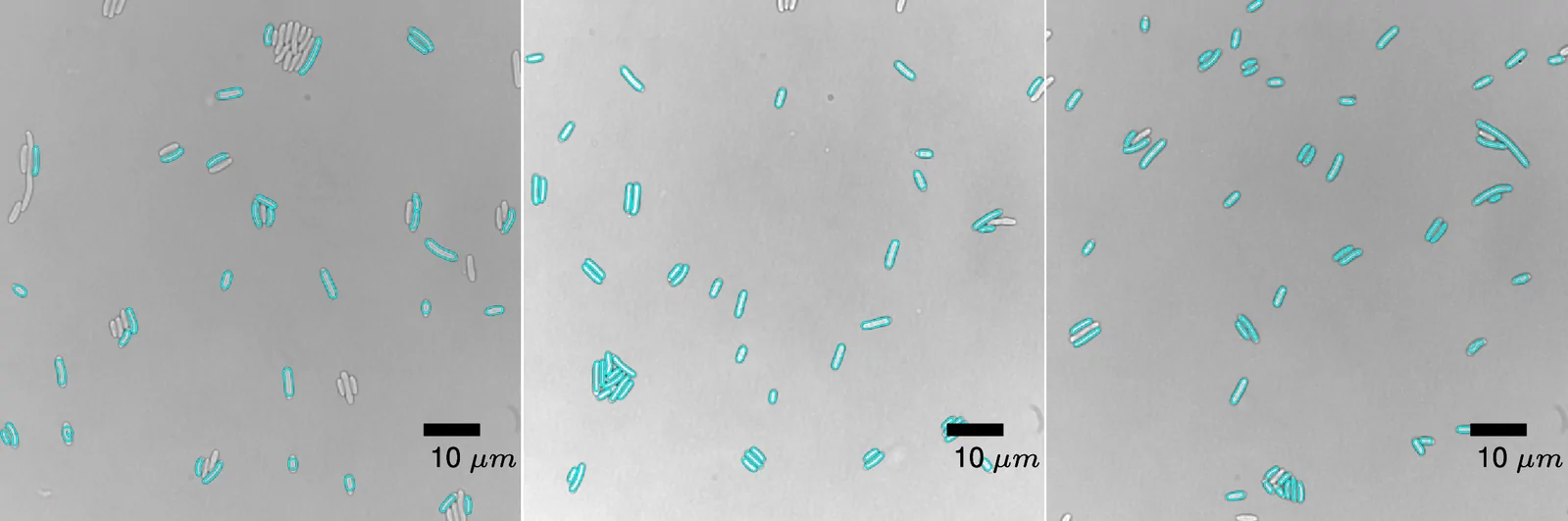
Microscopy images of cells treated with tetracycline. Each image represents a different replicate. We plotted the green channel of the recorded images in grayscale. The red channel, capturing propidium iodide activity, is overlaid in red. Bacterial shapes detected by the algorithm are outlined in cyan.

**Figure 2—figure supplement 14.**
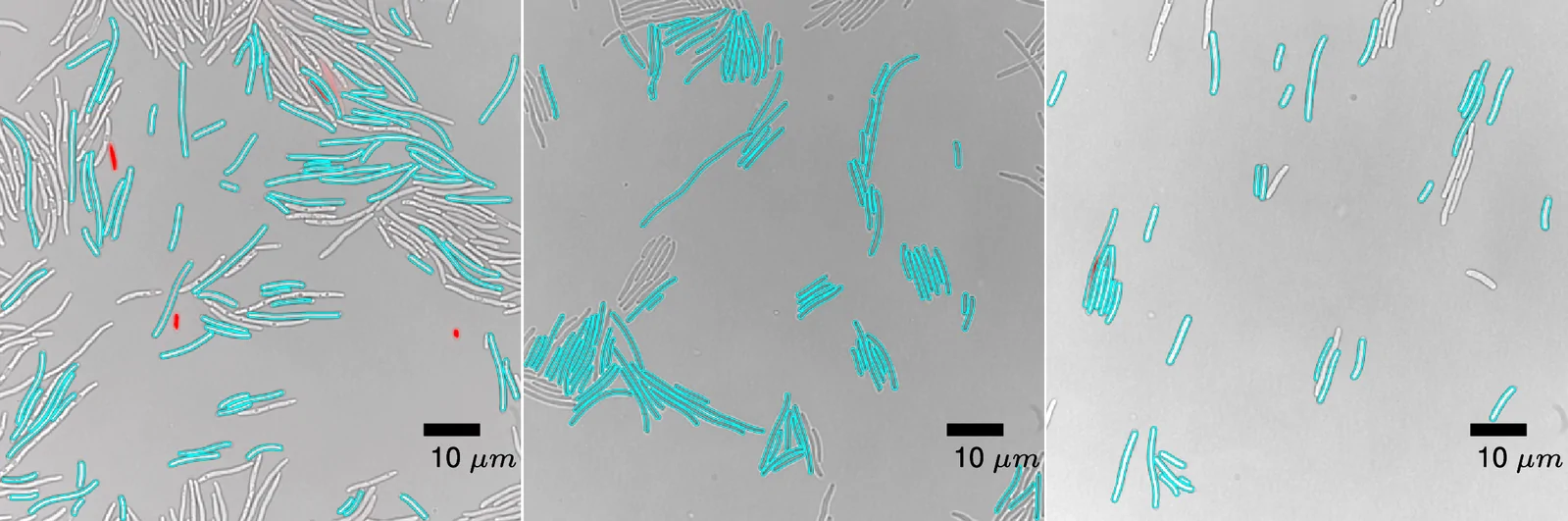
Microscopy images of cells treated with trimethoprim. Each image represents a different replicate. We plotted the green channel of the recorded images in grayscale. The red channel, capturing propidium iodide activity, is overlaid in red. Bacterial shapes detected by the algorithm are outlined in cyan.

For antibiotics where microscopy showed no significant filamentation (amoxicillin, colistin, fosfomycin, rifampicin, and tetracycline, Appendix 1—table 3), luminescence-based and CFU-based rates did not differ significantly (Figure 1, Appendix 1—table 2). In contrast, for those drugs where microscopy data indicated significant filamentation (ampicillin, ceftazidime, ciprofloxacin, meropenem, and trimethoprim), luminescence-based rates were significantly higher than CFU-based rates.

### Filamentation model predicts divergence between luminescence- and CFU-based rates

To further investigate the link between filamentation and the recorded light signal, we developed a simplified population dynamical model which incorporates bacterial filamentation (Appendix 1). In this model, we assume that cell-specific luminosity scales with cell volume – i.e., the volume-specific luminosity remains constant. We simulate single-cell growth by assuming that biomass acquisition is independent of cell size, which leads to linear elongation (Wang et al., 2010). A sudden reduction in the division rate by $\Delta\lambda < \lambda_{0}$ represents the onset of filament-inducing treatment and causes the mean cell volume to converge to a new, higher equilibrium (Appendix 1—figure 7).

As the mean cell volume stabilizes and cell-specific luminosity reaches equilibrium, the luminescence-based rate converges to the rate of change of population size $\psi_{B}$ (Appendix 1—figure 7). Depending on the division and death rates, this dynamic can result in an initial peak in light intensity followed by a decline (Appendix 1—figure 7b). In the model, linear elongation is a mathematically convenient simplification. In reality, how cells elongate may depend on the specific strain, morphology, and treatment. However, as the initial peaks in light intensity arise from cell volume converging to a new equilibrium, nonlinear elongation models can produce similar peaks.

We experimentally observed such peaks in light intensity for several drugs associated with filamentation, as predicted by the model for filamenting populations (Figure 1—figure supplement 2, Figure 1—figure supplement 4, Figure 1—figure supplement 8, Figure 1—figure supplement 10, Figure 1—figure supplement 11, and Figure 1—figure supplement 12).

**Figure 3.**
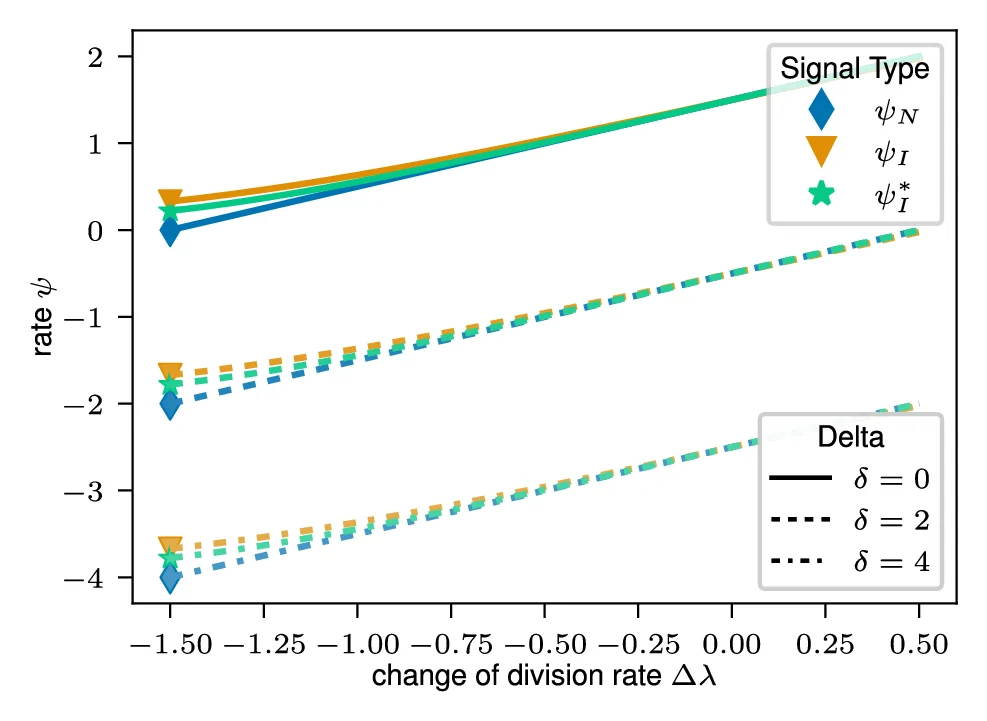
Simulations based on the filamentation model quantifying how changes in division rate due to treatment ($\Delta\lambda$) and death rate (δ) influence the rate of change of population size ($\psi_{B}$, blue), the rate of change of light intensity ($\psi_{I}$, orange), and the rate of change of light intensity when the first 2 hr of data are excluded ($\psi_{I}^{*}$, green). These illustrative simulations were conducted using an initial division rate $\lambda_{0}=1.5\,\mathrm{hr}^{-1}$. More details and all parameter values can be found in Appendix 1.

To investigate the dependence of $\psi_{B}$ and $\psi_{I}$ on treatment-induced changes in division rate and death rate (δ), we simulated 4 hr of treatment (details and parameters in Appendix 1). Our model shows that a reduction in λ leads to higher luminescence-based rates $\psi_{I}$ relative to the rate of change of population size $\psi_{B}$, with particularly large discrepancies when the death rate is low (Figure 3). The results from this model suggest that excluding early data points, where the mean cell volume changes rapidly, improves agreement between the estimated rates $\psi_{I}$ and $\psi_{B}$. This is evident in Appendix 1—figure 7b, where the slopes inferred from luminescence and from population size differ initially but are nearly identical at later times.

We tested this approach by refitting all experimentally acquired luminescence-based rates that exhibited an initial peak, excluding data points recorded before the light signal reached its maximum. The resulting distributions of $\psi_{I}^{*}$ (green) are shown in Figure 1. An exception was made for meropenem, for which we know the change of cell volume and thus applied an alternative correction as described below. This adjustment substantially reduced the difference between CFU- and luminescence-based estimates for all tested drugs and fully eliminated the discrepancy for mecillinam.

### Adjusting luminescence intensities by changes in volume narrows the gap between CFU- and luminescence-based rates

Given the model-predicted differences between $\psi_{I}$ and $\psi_{B}$ in filamenting populations, we next tested whether combining morphological data with measured light intensities can help infer $\psi_{B}$. This approach only works if the volume-specific luminosity is constant (Appendix 1, Equation 12). We used the mean cell volume data acquired by microscopy imaging before ($v_{\mathrm{obs},0}$, Figure 2a) and after treatment ($v_{\mathrm{obs},2\mathrm{h}}$, Figure 2b), for all drugs that caused significant filamentation (ampicillin, ceftazidime, ciprofloxacin, meropenem, and trimethoprim). The light intensities were then volume-corrected as $J(t)=I(t)\,v_{\mathrm{obs},0}\big/v(t)$, where v(t) is derived from the filamentation model (Equation 44, Appendix 1). The free parameters were determined by minimizing Equation 51. All adjusted light signals J are shown in Figure 1—figure supplement 1a, Figure 1—figure supplement 3, Figure 1—figure supplement 6, and Figure 1—figure supplement 12.

For ampicillin, the CFU-based and volume-corrected luminescence-based rates ($\psi_{J}$) did not differ significantly (Appendix 1—table 2). For ceftazidime, ciprofloxacin, and meropenem, volume correction reduced the discrepancy, but $\psi_{J}$ remained significantly above $\psi_{\mathrm{CFU}}$. We observed in all experiments that volume correction narrowed but never reversed the discrepancy ($\psi_{\mathrm{CFU}}\leq\psi_{J}\leq\psi_{I}$, Equation 23). From this observation and the derivation in the SI (Appendix 1), we conclude that $\psi_{I}$ is closer to $\psi_{V}$ (rate of total cell volume change) than to $\psi_{B}$ (rate of bacterial number change).

Three factors may explain these residual differences: (i) the assumption of constant volume-specific luminosity may not hold; (ii) the approximation of v(t) may be inaccurate, and excluding entangled or overlapping cells from the analysis introduces a bias that underestimates the volume of heavily filamented cells; (iii) the CFU-based method may underestimate $\psi_{B}$.

For ciprofloxacin, the large disparity between $\psi_{\mathrm{CFU}}$ and $\psi_{I}$ is unlikely to be explained by factors (i) and (ii) alone. Bringing the two rates into agreement solely by adjusting cell-specific luminosity would require an almost four-order-of-magnitude increase, which appears implausible. We therefore conclude that, in this case, CFU-based measurements likely overestimate the rate of population decline, corresponding to an underestimation of $\psi_{B}$ (iii).

### CFU-based estimates can overestimate the rate of population decline

After exploring why luminescence assays can underestimate the rate of population decline, we now explore why CFU assays may overestimate it. The rate of change of the CFU signal, $\psi_{\mathrm{CFU}}$, only matches $\psi_{B}$ if the number of colonies emerging per plated bacterium, $\eta\in[0,1]$ (Equation 4), is constant over time (Appendix 1, Equation 6). This assumption can be problematic for three main reasons:

- Loss of culturability: The number of colonies emerging per plated bacterium, η, depends on the division rate λ, which can be affected temporarily or permanently by treatment (Eagle and Musselman, 1949), e.g., due to DNA damage. In extreme cases, viable and culturable cells can be converted into viable but non-culturable cells (Besnard et al., 2002; Oliver, 2005; Li et al., 2014), meaning they continue to be metabolically active but cease to divide (λ=0) and therefore no longer form colonies on agar. Typically, cells can reproduce at the start of a time-kill assay but may, depending on the drug, partially or completely lose this ability as the assay progresses, causing an underestimation of $\psi_{B}$.
- Antimicrobial carryover: Antibiotics transferred onto agar by plating a diluted culture can have a residual treatment effect on either the division rate λ or the death rate δ, depending on the mode of action of the drug, thereby reducing the probability of colony formation. This phenomenon, known as antimicrobial carryover, has been described in previous studies (Pearson et al., 1980; Eng et al., 1991; Coates et al., 2018). Its effect is usually minimal at the start of a time-kill assay, when bacterial density is high and plated samples are highly diluted. However, as the assay progresses and bacterial density declines, less dilution is needed, increasing the concentration of the transferred antibiotic. As a consequence, CFU-based rates would underestimate $\psi_{B}$ between two time points $t_{0}$ and $t_{1}$ by $\frac{\ln(\eta_{1})-\ln(\eta_{0})}{\Delta t}$, where $\eta_{i}$ denotes the mean number of colonies formed per plated bacterium at time $t_{i}$.
- Aggregation: Filamentation or altered cell adhesiveness can change the distribution of colony-initiating cluster sizes on agar after plating (Equation 3). Changes in cluster size in turn affect the average number of clusters per plated bacterium, thereby biasing estimates of $\psi_{B}$.

### Partial loss of culturability causes CFU to underestimate $\psi_{B}$ for ciprofloxacin and trimethoprim treatment

During ciprofloxacin treatment, CFU counts fell steeply while light intensity continued to rise (Figure 1—figure supplement 6). This discrepancy is consistent with previous reports comparing CFU and luminescence during fluoroquinolone killing (Salisbury et al., 1999; Marques et al., 2005). To investigate the cause of this discrepancy, we plated treated cultures on phosphate-buffered saline (PBS) agar containing propidium iodide (PI), a red fluorescent dye that binds to nucleic acids but cannot penetrate intact cell membranes. Microscopic imaging (Figure 2—figure supplement 7) revealed almost no red fluorescence, indicating that the cells remained impermeable. Although impermeability alone does not confirm viability, additional observations support the conclusion that most cells were still alive: the absence of bacterial debris (as has been observed for drugs with similar decline in CFU such as amoxicillin), visible growth indicated by increased cell size compared to 2 hr earlier, and continued (and even increased) light emission. These findings suggest that most cells remain alive but are unable to form colonies under the provided conditions, possibly due to DNA damage induced by ciprofloxacin (Levine et al., 1998). This observation aligns well with previous studies on ciprofloxacin, which found that CFU can underestimate viability relative to non-culture-based methods (Besnard et al., 2002; Wu et al., 2024; Fanous et al., 2025).

Trimethoprim treatment showed similar, though less pronounced, results (Figure 1—figure supplement 18). Trimethoprim, which impairs DNA replication (Gleckman et al., 1981), likewise caused an increase in light intensity and a decline in CFU counts, while microscopy revealed intact, mostly impermeable, filamented cells (Figure 2—figure supplement 14).

### Antimicrobial carryover causes underestimation of $\psi_{B}$ for pexiganan using CFU

Building on our understanding of when luminescence assays accurately estimate $\psi_{B}$, we hypothesized that AMPs would be an ideal application for this method. We expected that, during the AMP’s short killing phase, changes in the cell-specific luminosity would remain negligible compared to the high kill rates AMPs can achieve.

Initially, however, we failed to recover almost any colonies on agar, despite the light intensity, indicating a high enough bacterial density. Moreover, colony counts were inconsistent across dilutions: 100-fold and 1000-fold dilutions from the same cultures yielded similar colony numbers, instead of reflecting the tenfold difference. We suspected that AMPs from the liquid culture, including those attached to the bacterial surface, were carried over into the PBS dilution medium, causing continued cell death during dilution and after plating.

To test this, cultures treated with pexiganan for 1 min were diluted 1:100 in PBS supplemented with various concentrations of CaCl_2_ and MgCl_2_. These compounds were selected based on prior evidence that they inhibit the activity of other AMPs (Deslouches et al., 2005). We sampled and plated at four time points from these diluted cultures, approximately 45 min apart.

Our results show that supplementing the dilution medium increased the measured CFU substantially (Appendix 1—figure 4, Appendix 1). Conversely, diluting in unsupplemented PBS did not stop bacteria from dying. Supplementing 100 mM MgCl_2_ yielded the highest CFU count for the first time point (Appendix 1—table 4). Since CFU cannot systematically overestimate bacterial density, this count represents the best estimate of the bacterial density. Consequently, we supplemented the PBS with 100 mM MgCl_2_ in subsequent pexiganan experiments.

Given this insight into the residual killing effect of pexiganan and how to mitigate it, we repeated the CFU time-kill experiment using two different dilution media: pure PBS and PBS supplemented with 100 mM MgCl_2_ (Appendix 1). We recorded three replicates for each of the two time-kill curves and counted colonies on all agar plates from three dilution steps for both dilution media. To lower the detection limit by one order of magnitude, we increased the plated volume from 10 μL to 100 μL. Since this volume exceeds the capacity of the automated high-throughput setup, we used the standard manual CFU plating method instead.

We observed a much steeper initial decline in CFU for the cultures diluted in pure PBS compared to the supplemented ones (Appendix 1—figure 5). In pure PBS, more highly diluted samples consistently yielded higher CFU estimates (Appendix 1—figure 5a), supporting the antimicrobial carryover hypothesis. This pattern diminished over time, suggesting a reduction in the residual killing effect of pexiganan, which we discuss below.

### Luminescence and CFU show identical decline rates for pexiganan time-kill curves if residual killing is prevented

To confirm that eliminating residual pexiganan killing aligns CFU and luminescence, we supplemented PBS with 100 mM MgCl_2_ and measured both signals at pexiganan concentrations of 8 μg/mL and 16 μg/mL using the ‘rapid luminescence-CFU assay setup’ (Materials and methods). We observed no significant difference between the CFU- and luminescence-based rates for either of the tested pexiganan concentrations (Figure 1b, Appendix 1—table 2). However, examining the time series (Figure 1—figure supplement 19) revealed that while CFU and luminescence signals declined in parallel for the 8 μg/mL treatment, they diverged for the 16 μg/mL kill curve. In this case, the CFU signal initially declined much faster (rates below $-200\,\mathrm{hr}^{-1}$), and subsequently declined more slowly than the corresponding luminescence signal, ultimately resulting in a similar average rate (approximately $-60\,\mathrm{hr}^{-1}$).

### Pexiganan rapidly loses killing capacity

During the pexiganan experiments, we observed an initial steep decline in bacterial density, predicted by both CFU and luminescence, followed by almost constant signals (Figure 1—figure supplement 19, Appendix 1—figure 5). Two possible, non-exclusive explanations for this observation are: first, pexiganan is deactivated or sequestered from the medium by attaching to targets on the bacteria over time; and second, the remaining bacteria are unaffected by the AMP because they are resistant or persisters. To investigate the first explanation, we exposed bacteria to pexiganan for 5 min at 16 μg/mL, after which the supernatant was collected and tested for its ability to kill bacteria (Appendix 1). While the initial treatment showed rapid bacterial killing ($\psi_{\mathrm{CFU}}\approx-46\,\mathrm{hr}^{-1}$ between $t_{0}=0$ and $t_{1}=5\,{\mathrm{min}}$), bacteria exposed to the supernatant alone showed no significant reduction in viability (Appendix 1—figure 6, Appendix 1—table 5). These results show that the supernatant has no residual killing effect. This makes deactivation or sequestration through the attachment of pexiganan the likely explanation for the flattening CFU signal, even though we did not assess whether the surviving cells are resistant or persisters.

## Discussion

We evaluated whether luminescence can serve as a high-throughput proxy for population dynamics by comparing it with CFU assays. We found no significant difference between CFU- and luminescence-based rates for treatments that neither induce substantial changes in culturability nor provoke strong morphological changes, such as filamentation. However, for drugs that induce filamentation and/or loss of culturability, the two methods can yield significantly different results. The divergence between the two rates does not imply that either method is incorrect; rather, CFU and luminescence capture different population properties.

The CFU method counts bacteria capable of forming colonies on permissive media (i.e. culturable cells). When inferring growth rates from CFU, the observed rate of change $\psi_{\mathrm{CFU}}$ reflects both the rate of change of population size $\psi_{B}$ and changes in the probability that a plated bacterium forms a colony, η (Equation 4). The CFU-based rate equals $\psi_{B}$ only if η remains constant over time.

However, η can change for three main reasons. First, altered clustering behavior can change how many bacteria seed a single colony. This directly shifts the observed colony count. Second, physiological changes to the bacteria may lead to a temporary or permanent change in culturability (Eagle and Musselman, 1949; Baquero et al., 1986; Besnard et al., 2002; Oliver, 2005; Li et al., 2014; Wu et al., 2024), which may increase the fraction of viable cells that fail to form colonies, e.g., due to a reduced division rate. Third, residual drug activity carried over to the agar can alter on-plate conditions, thereby reducing division or increasing the death rate (Pearson et al., 1980; Eng et al., 1991; Coates et al., 2018).

Preventing antibiotic carryover when handling low-density cultures treated with highly concentrated antimicrobials is challenging and, in some cases, infeasible. Centrifugation-based washing (pelleting bacteria and replacing the supernatant) can remove residual drug, but only if the processing delay is negligible relative to the antibiotic’s killing kinetics – a condition unlikely to hold for fast-acting agents such as AMPs. Moreover, although bacteria generally tolerate high centrifugal forces (Deguchi et al., 2011), the impact on compromised cells, such as those with destabilized walls, remains unknown. As an alternative, we found that supplementing the dilution medium with MgCl_2_ effectively neutralizes residual AMP activity, preventing residual killing effects in CFU assays for pexiganan. However, this strategy is not generalizable, as for many antimicrobials, the corresponding deactivating agents are unknown – or may not even exist. For these cases, it may be impossible to accurately derive $\psi_{B}$ from CFU counts at high drug concentrations.

In contrast to CFU assays, luminescence assays become more reliable at high, fast-killing concentrations. This is because $\psi_{I}$ reflects both the rate of population size change, $\psi_{B}$, and changes in cell-specific luminosity (Equation 9); when population declines rapidly, $\psi_{I}$ converges to $\psi_{B}$.

One potential exception is the delay between the decline of CFU and the decline of the luminescence signal, observed during the pexiganan experiments. This discrepancy may arise from two effects: either the CFU signal declines more steeply or the luminescence signal declines more slowly than the number of living cells. CFU may overestimate the decline, as damaged but living cells have a reduced probability of forming colonies. Conversely, luminescence may underestimate the decline if there is a delay between cell death and cessation of luminescence. For antimicrobials that lyse cells (such as pexiganan), we would expect a rapid, though not instant, cessation of luminescence due to the dilution of all reactants. The delay may be more pronounced for drugs that kill without lysing cells. However, metabolically active and impermeable cells are difficult to characterize as dead in the first place. Based on our experimental data, we cannot distinguish between these possibilities and therefore cannot exclude that luminescence assays underestimate extremely rapid kill rates.

For lower kill rates, we observed that the absence of filamentation was a good indicator of stable cell-specific luminosity. In cases where drugs induced filamentation, CFU- and luminescence-based rates diverged. We further demonstrated that correcting for the increased cell size partly compensates for the difference between CFU- and luminescence-based estimates, and we found that the rate of change in light intensity is closer to the change in total cell volume than to the change in total cell number (Appendix 1). This makes a constant volume-specific (or mass-specific) luminosity a better assumption than a constant cell-specific luminosity.

Changes in both CFU and luminescence are used as proxy signals for population growth rates (Regoes et al., 2004; Kishony and Leibler, 2003; Yeh et al., 2006; Chait et al., 2007; Foerster et al., 2016; Kavčič et al., 2020; Angermayr et al., 2022). Whether discrepancies between the changes in these proxy signals and the changes in living bacteria pose a problem depends on the underlying biological question. The rate of change of living bacteria, $\psi_{B}$, is most commonly applied in theoretical modeling to create predictions, making its estimation important. If, instead, the aim is to assess a population’s reproductive potential, for example, in studies focusing on evolutionary dynamics, examining changes in the number of culturable cells (as approximated by CFU) may be more relevant than $\psi_{B}$, as only culturable cells contribute to subsequent generations and thus to evolution. Tracking changes in total biomass (closer to $\psi_{I}$) can be more relevant than the number of living bacteria, as biomass accounts for a potential ‘catch-up’ effect, whereby filamented cells fragment into multiple viable units once antibiotic pressure is removed (Baquero et al., 1986; Cayron et al., 2023).

A notable challenge of using luminescence assays is the absence of a fundamental biological principle linking light intensity uniquely to a single population property. Beyond cell number and biomass, the light intensity can depend on treatment-induced metabolic changes, which potentially explain some of the discrepancies between luminescence- and CFU-based rates observed. Furthermore, the availability of nutrients can influence luminosity, limiting assays to timeframes during which the nutritional availability remains stable.

A further subtlety arises from within-population heterogeneity: cell-specific luminosity can vary between individual bacteria. Such variation may bias population-level estimates if it co-varies with the susceptibility to antimicrobials.

Measuring population decline is challenging. In this work, we addressed some of the complexities involving the luminescence method, but several questions remain open for follow-up work. Our study was motivated by the question of whether changes in light intensity follow those in cell number, and we therefore used CFU as the reference metric. Supplementing these experiments with microscopy, we found that light intensity tracks cumulative cell volume more closely than cell number. A natural next question is therefore how closely luminescence tracks biomass – addressing it, however, requires a reference method designed to capture volume or mass directly, rather than cell count.

Generalizability beyond *E. coli* is a second open question: while the core principle that larger cells emit more light should hold broadly, the morphological and physiological responses to antimicrobial treatment may be strain-specific, so the drug-specific observations do not necessarily transfer across species. A related caveat is that most drugs in this study were tested at a single concentration, leaving the concentration dependence of these drug-specific findings still to be established.

Our results show that neither CFU nor luminescence is optimal for every experimental scenario. Instead, CFU and luminescence work best under different conditions, measure different population properties, and complement each other. At low and intermediate drug concentrations, changes in CFU accurately reflect changes in bacterial density, but CFU becomes unreliable at high drug concentrations.

Luminescence, by contrast, becomes more reliable at high concentrations, where CFU becomes unreliable. In practice, the luminescence method significantly reduces labor, consumables, and costs: eight PD curves with twelve concentrations and four replicates each can be fit on a single 384-well plate, whereas measuring CFU would require more than 8000 agar plates, hundreds of dilution plates, and substantial manual labor. Given their scalability and cost-effectiveness, luminescence assays offer a valuable alternative for high-throughput analysis, particularly at high antimicrobial concentrations, where traditional methods become unreliable or even unusable.

## Materials and methods

**Key resources table:** 

Reagent type (species) or resource	Designation	Source or reference	Identifiers	Additional information
Strain	K-12 substr. MG1655	Lab collection	GenBank: U00096	Parent strain
Strain	MG1655 *galK*::*luxCDABE-kan*	This paper	Available on request	Bioluminescent reporter; *λ*-Red integration replacing *galK*
Recombinant DNA reagent	pCS-*λ*	Kishony and Leibler, 2003		Source of *luxCDABE*, *λ*-Pr promoter, and kanamycin cassette
Recombinant DNA reagent	pSIM5	Datta et al., 2006		*λ*-Red integration helper plasmid
Sequence-based reagent	Integration primers	This paper	Appendix1—table 6	For *λ*-Red integration
Drug	Antimicrobials		Appendix1—table 1	Suppliers, catalog numbers, and MICs
Software	Analysis scripts, work list generation, colony recognition, and model code	Zenodo	10.5281/zenodo.21321595	
Other	Experimental datasets	Zenodo	10.5281/zenodo.21321592	
Other	Evo 200 liquid-handling platform	Tecan		Automated CFU plating
Other	STX100 incubator	Liconic		Automated incubator
Other	Infinite F200 plate reader	Tecan		Luminescence reads
Other	Pickolo camera	SciRobotics		Colony imaging
Other	Eclipse Ti2 microscope + DS-Qi2 camera	Nikon		Single-cell imaging

### Strains

Key strains, reagents, and software used in this study are listed in the Key resources table. We generated a bioluminescent strain by integrating a modified *P. luminescens luxCDABE* operon, driven by the constitutive λ-Pr promoter, together with a kanamycin resistance cassette (as a marker) into the chromosome of *E. coli* MG1655. This integration replaced the galK gene and was achieved using λ-Red recombination (Datsenko and Wanner, 2000), following a protocol by Hughes et al., 2013 (19–26) using the helper plasmid pSIM5 (Datta et al., 2006). The integrated elements were derived from the pCS-λ plasmid (Kishony and Leibler, 2003; Bjarnason et al., 2003). Primers are listed in Appendix 1—table 6. For all time-course experiments, we prepared three replicate exponential cultures by diluting overnight cultures (grown for approximately 18 hr) 1:100 and growing them to exponential phase for 1–1.5 hr.

### Media

We used LB (Sigma L3022) as a liquid medium and, as a solid medium for CFU plating, LB with 1.5% agar. Cultures were treated by diluting the tenfold working concentration of 1 of 20 antimicrobials 1:10. All MICs, determined by broth microdilution (European Committee on Antimicrobial Susceptibility Testing (EUCAST), 2025), and the concentrations used are listed in Appendix 1—table 1. Working concentrations were centered around the MIC, with some variation due to rounding convenience and variability in repeated MIC tests. For colistin and polymyxin B, we used lower concentrations, as higher concentrations in our setup consistently yielded too few colonies for meaningful analysis.

PBS (Sigma 79383) was used as the diluent for CFU assays. If cultures were treated with pexiganan, 100 mM MgCl_2_ was added. For microscopy, we added 1 μg/mL PI to the liquid medium and used PBS/PI agar plates (containing PBS with 1.5% agar and 1 μg/mL PI) as solid medium.

### Automated CFU plating

We automated the high-throughput colony-count method described by Jett et al., 1997, using an Evo 200 liquid-handling platform (Tecan) integrated with a Liconic STX100 incubator. The platform handles liquids and automatically moves, images, and incubates plates. We produced six colony streaks by spotting six 10 μL drops of diluted bacterial culture onto a one-well agar plate. Plates were automatically tilted for 7 s on a custom-built tilter integrated into the platform, to spread the drops and distribute the bacteria. After incubation, plate images were captured using the Pickolo camera (SciRobotics).

The platform is controlled by custom-generated worklists executed in the native software ‘Evoware’. These worklists were generated using the Python package pypetting (version 1.0.1). We analyzed the captured images of the agar plates using a custom colony-recognition script that automatically identifies colonies and allows the manual addition of unidentified colonies and the removal of mismatched ones. All Python classes for generating the worklists and analyzing colonies are available at Zenodo (DOI: 10.5281/zenodo.21321595).

### Luminescence measurements

To record the luminescent light intensity, we used an Infinite F200 spectrophotometer plate reader (Tecan), which is also integrated into the liquid-handling platform, with an exposure time of 1 s. We set $20\;\mathrm{rlu}$ as the lower detection limit and excluded all data points below.

### Luminescence-CFU assay setup

To measure the CFU and light intensity at seven time points, we treated the exponential cultures and then distributed them onto seven (one for each time point) white 384-well plates (Greiner, 781073), with each culture well containing 54 μL medium and 6 μL 10× stock solution. We adjusted the duration of the experiments between 2 and 5 hr, depending on the anticipated kill rate. For each time point, an assay plate was transferred from the incubator to the plate reader for luminescence measurement. Subsequently, a dilution series was conducted directly in the white plate and plated using the automated plating method, after which the plate was discarded.

### Rapid luminescence-CFU assay setup

This experiment is a variation of the *Luminescence-CFU assay setup*, adjusted to measure rapid kill curves for the AMP pexiganan. In this setup, we captured four time points within 5 min. Cultures were treated in a 96-deep-well plate (Greiner, 780285) by adding 100 μL of the 10× stock solution to 900 μL exponential phase culture. 60 μL of the treated culture was then transferred to a 384-well white plate (Greiner, 781073) and placed in the plate reader for continuous luminescence recording. For the four CFU time points, samples were taken directly from the deep-well plate, automatically diluted in PBS supplemented with 100 mM MgCl_2_ in a 96-well plate (Greiner, 655101) to halt the antimicrobial activity and then plated.

### Morphology experiments

To assess treatment-induced morphological changes, we imaged treated (for 2 hr) and untreated bacteria by spotting 2 μL droplets onto PBS/PI-agar plates. The spots were cut out and flipped onto Ibidi μ-dishes (Ibidi, 80136) for imaging. We used an Eclipse Ti2 microscope (Nikon) with a 100× objective connected to a DS-Qi2 Nikon Scientific CMOS (sCMOS) camera to image the bacterial cells. The microscope setup included an additional 1.5× zoom, which was used only for some images due to unintentional variation. We estimated the width and length of the bacterial cells using a custom Python script, as described in Appendix 1.

### Fitting rates of change ψ

To compare the rates of change of two signals, we first excluded all data below the detection limits (empty plates or light intensity below $20\,\mathrm{r}\mathrm{l}\mathrm{u}$). We then truncated both signals at the latest time point where both remained above the detection limit, ensuring the same time frame was used for comparison. Next, we bootstrapped 2000 datasets with replacement per signal, while ensuring that each dataset contained more than one time point. For each dataset, we applied a simple regression to fit an exponential function to all time points of each time-kill curve, resulting in distributions with 2000 rate estimates each.

### Significance

We classify two distributions of rates as not significantly different (n.s.) if the mean of each distribution falls within the 95% confidence interval of the other. Otherwise, we classify them as significantly different (*).

## Figures and Tables

**Appendix 1—figure 1.**
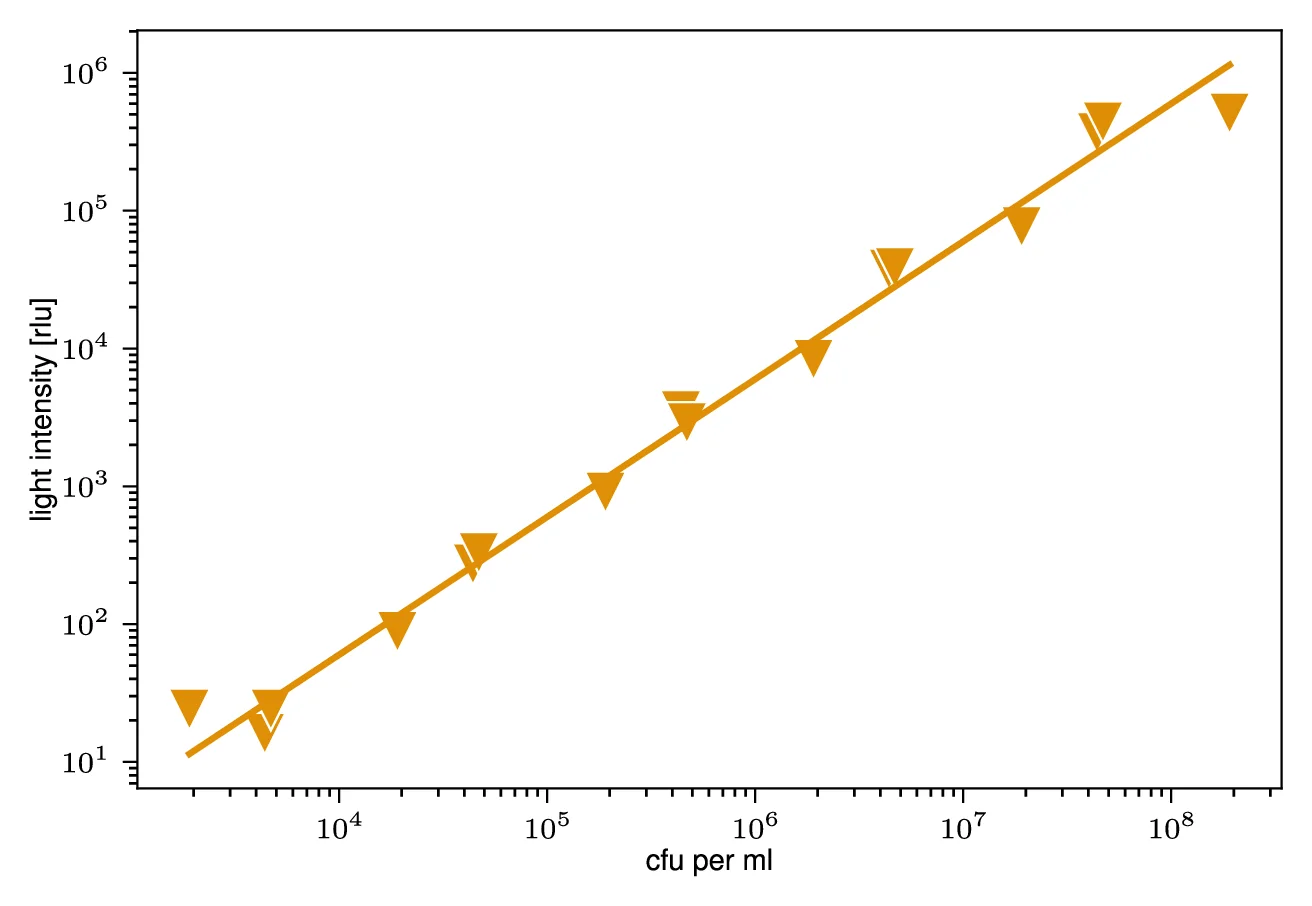
Light intensity scales linearly with bacterial density. Serial tenfold dilutions of bacterial cultures were prepared in a 384-well white microplate, and luminescence was measured immediately. Linear regression of the luminescence signal against bacterial density (CFU) yielded a conversion factor of $m_{\mathrm{fit}}=0.006\,\mathrm{rlu\cdot mL\cdot CFU}^{-1}$. The high correlation ($R^{2}=0.987$ in log-log space) confirms a linear relationship between luminescence and bacterial density.

**Appendix 1—figure 2.**
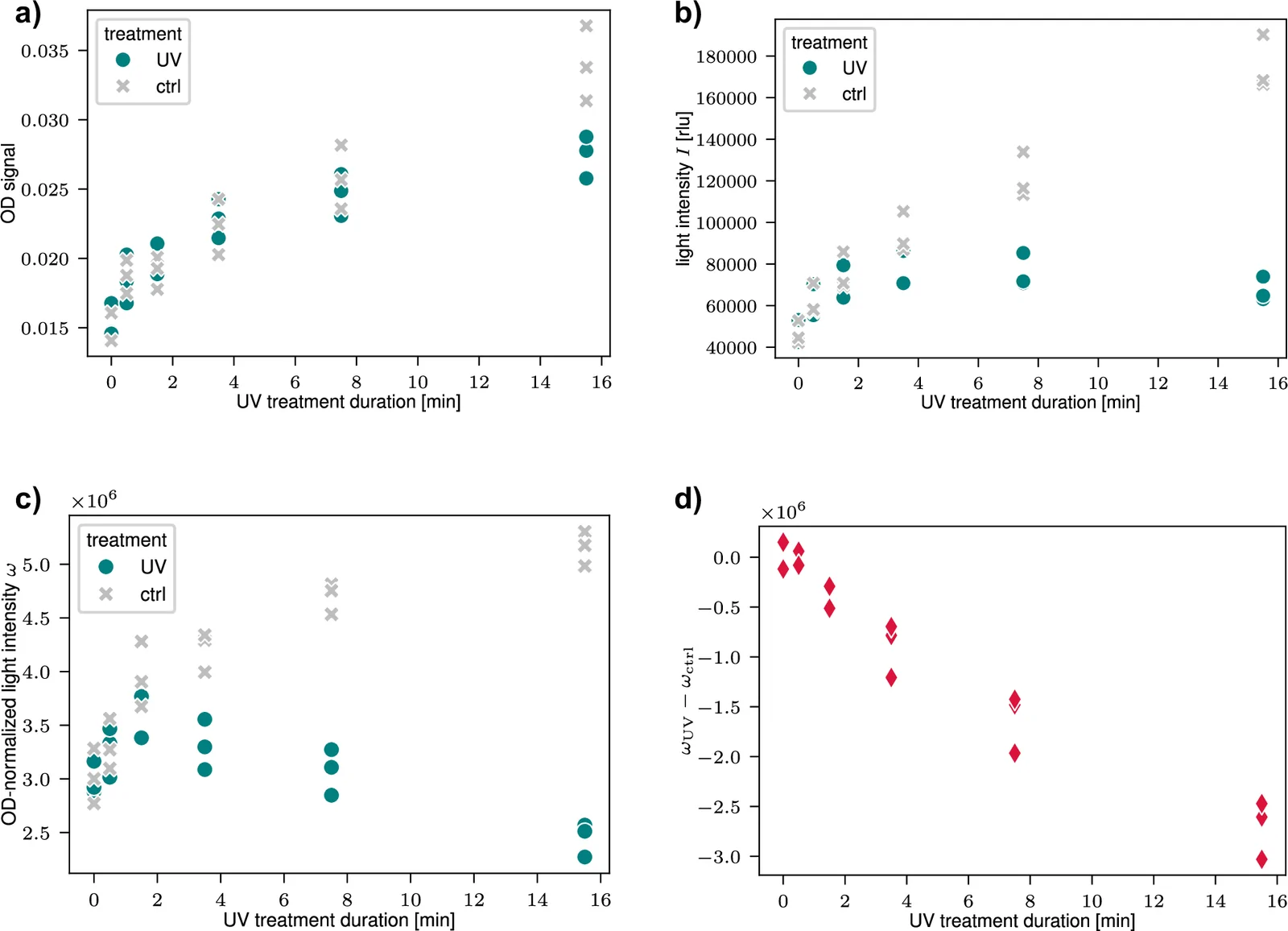
Panel plot showing the effects of UV treatment on bacterial density (approximated by optical density [OD]) and light intensity (I) over time by comparing treated (UV) and untreated (ctrl) cultures. (**a**) OD, (**b**) light intensity, (**c**) OD-specific light intensity $\omega=I(t)\big/\mathrm{OD}(t)$, and (**d**) the difference in OD-specific light intensity between UV-treated and control.

**Appendix 1—figure 3.**
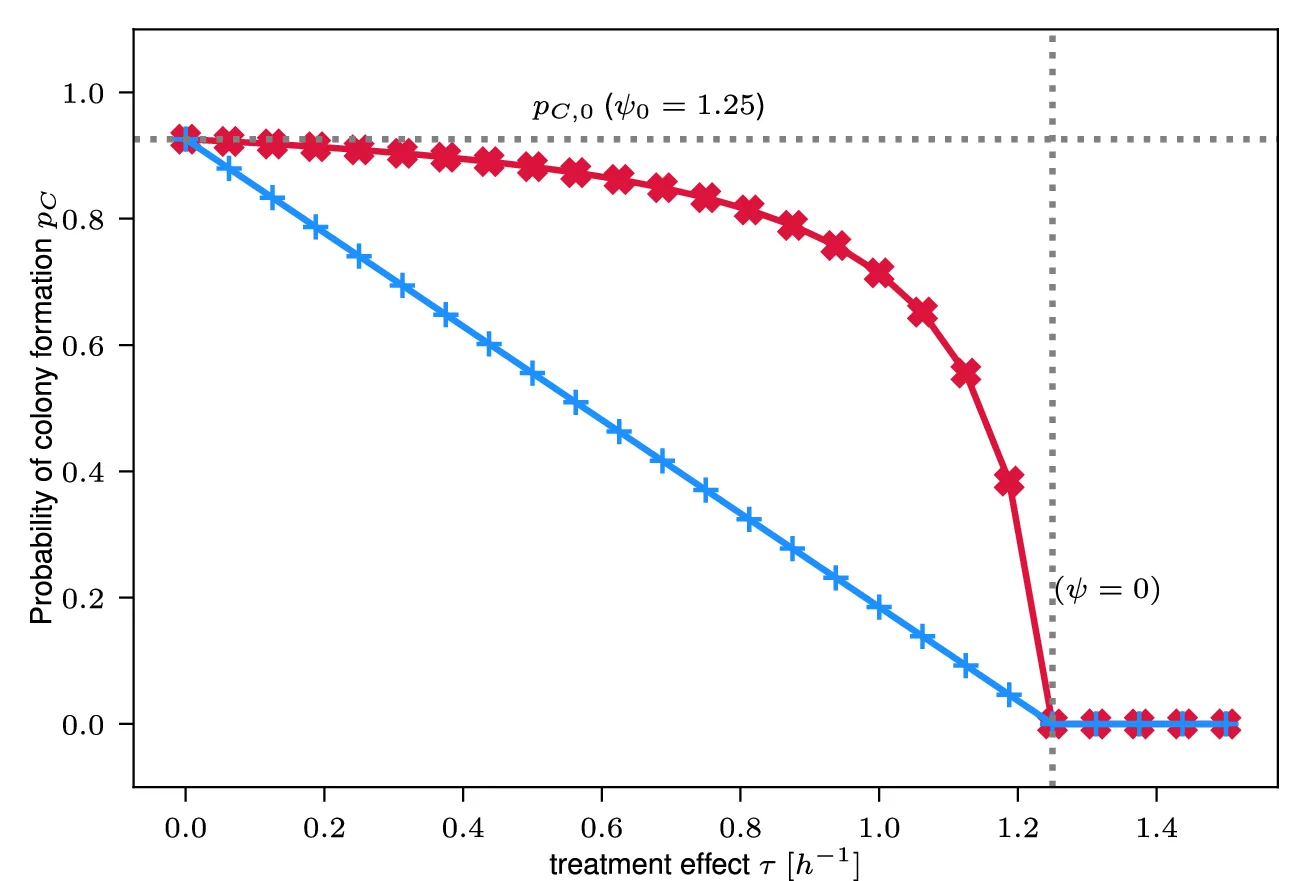
Example of the probability of colony formation $p_{C}=1-p_{E}$ (see Appendix 1) for a single plated bacterium. Blue shows $p_{C}$ for a purely bactericidal drug ($\lambda_{T}=0$) and red for a purely bacteriostatic drug ($\delta_{T}=0$), plotted over the treatment effect $\tau=\delta_{T}+\lambda_{T}$. In this illustrative example, we use $\lambda_{0}=1.35\,h^{-1}$ and $\delta_{0}=0.1\,h^{-1}$.

**Appendix 1—figure 4.**
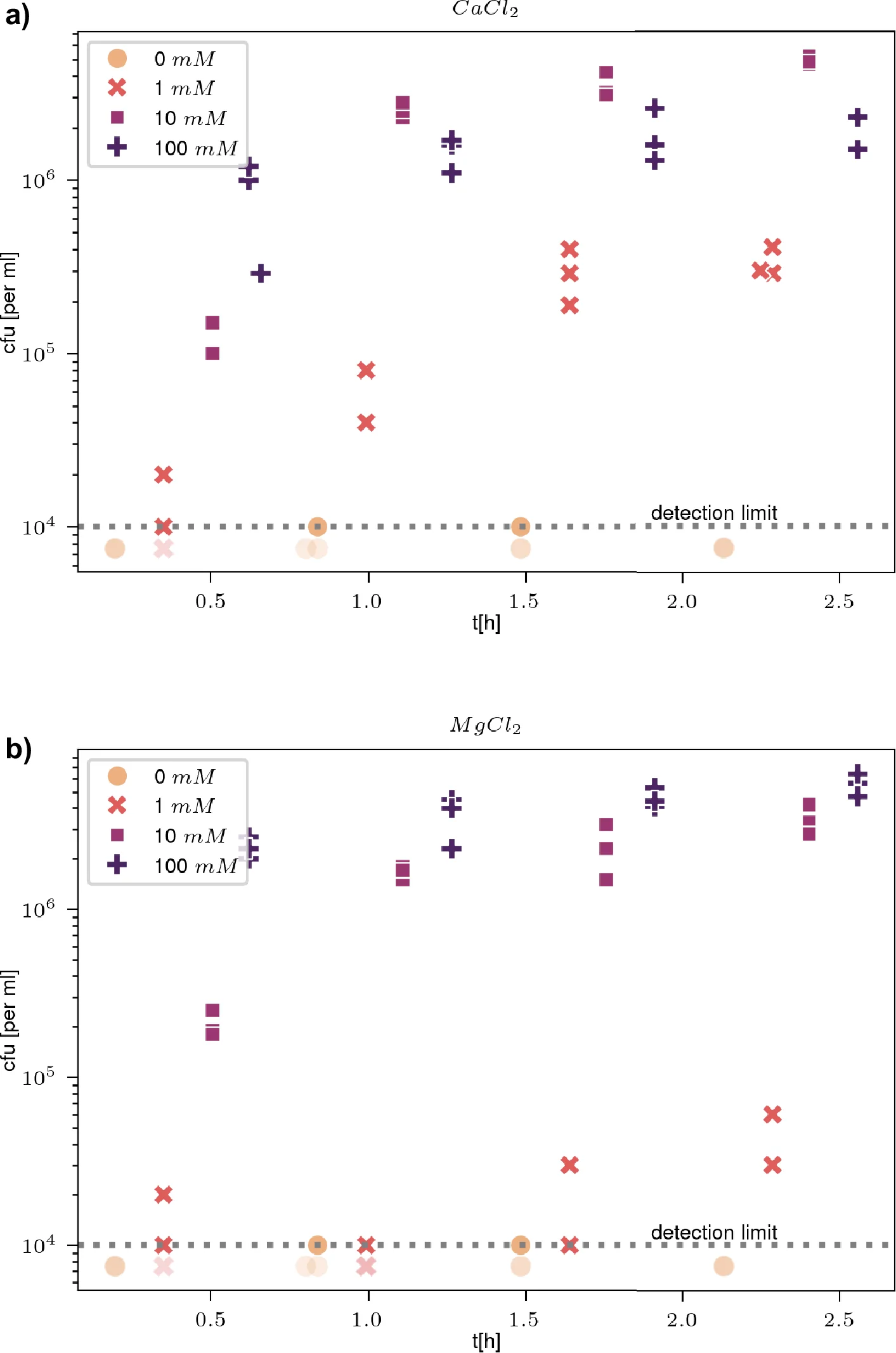
Colony-forming unit (CFU) measured over time in supplemented dilution media. Bacterial cultures treated for 1 min with 16 μg/mL pexiganan were diluted (1:100) in phosphate-buffered saline (PBS) supplemented with varying concentrations of (**a**) CaCl_2_ and (**b**) MgCl_2_. Diluted samples were repeatedly plated over time to test whether supplementation prevents further bacterial killing.

**Appendix 1—figure 5.**
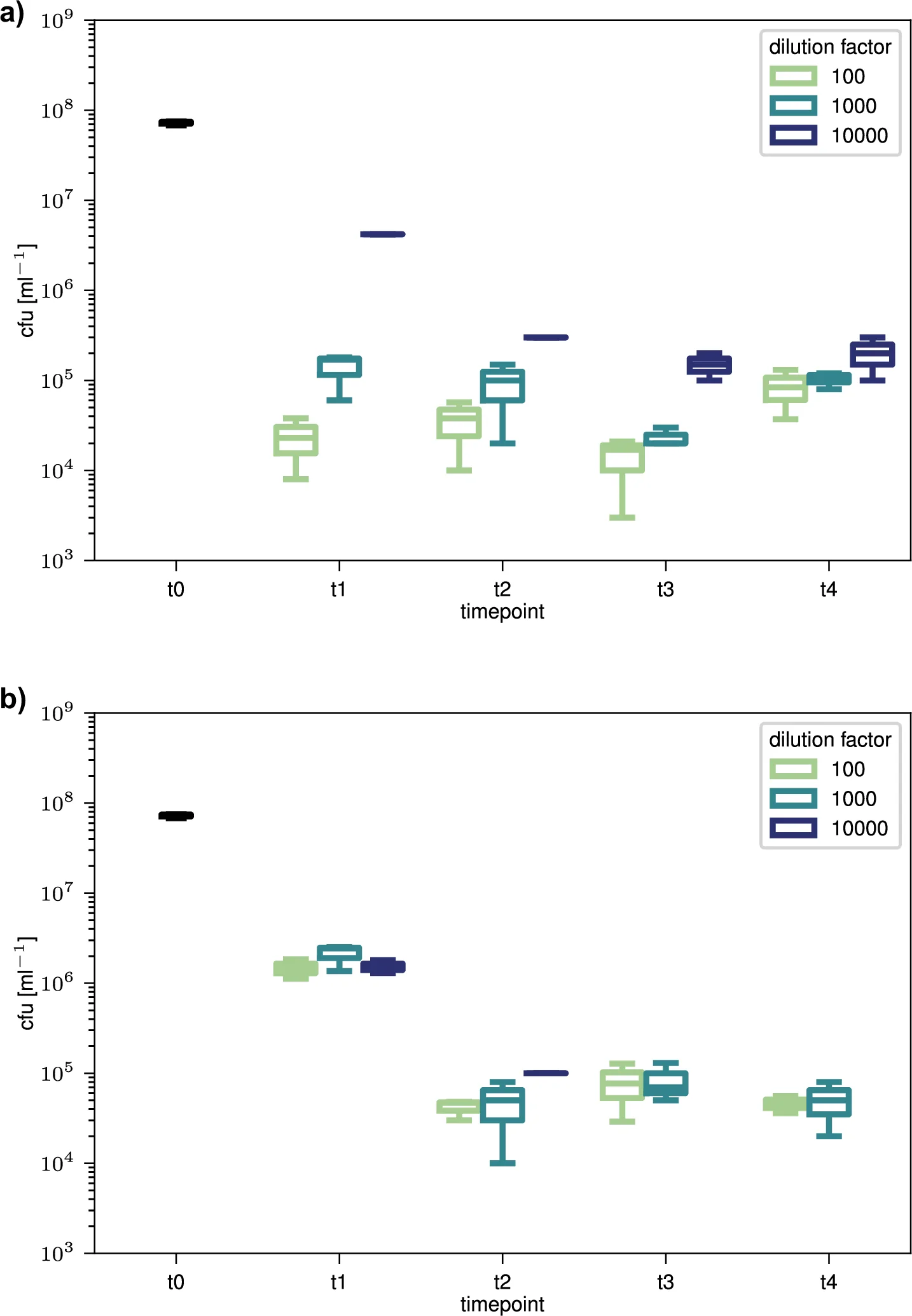
Colony-forming unit (CFU) time-kill curves for pexiganan, performed manually, comparing dilution in (**a**) unsupplemented phosphate-buffered saline (PBS) and (**b**) PBS supplemented with 100 mM MgCl_2_. Time points correspond to: $t_{0}\approx 0\,\mathrm{s}$, $t_{1}\approx 20\,\mathrm{s}$, $t_{2}\approx 2\,\mathrm{min}$, $t_{3}\approx 3\,\mathrm{min}\,20\,\mathrm{s}$, and $t_{4}\approx 5\,\mathrm{min}$. The black boxplot ($t_{0}$) indicates the pretreatment bacterial density.

**Appendix 1—figure 6.**
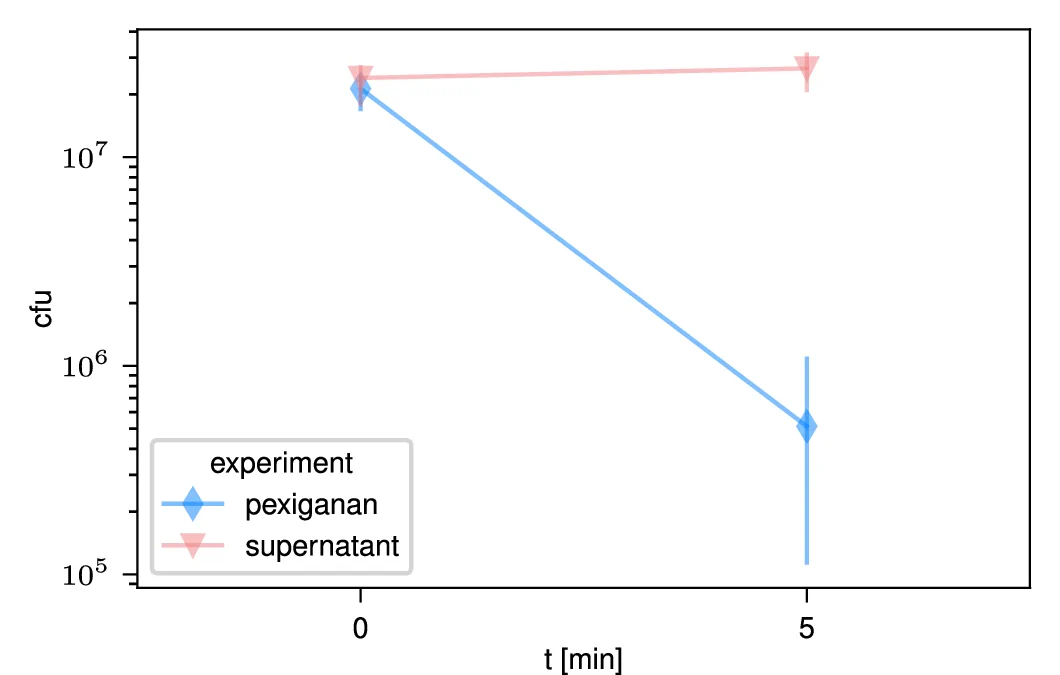
Pexiganan kill curve versus supernatant kill curve. We show the original pexiganan kill curve in blue and the change of colony-forming unit (CFU) over time in the supernatant. The error bars show the min/max interval of the three replicates. Appendix 1—table 5 lists the confidence interval and mean for the bootstrapped rates. According to the significance criterion (defined in Materials and methods), these two rates are significantly different. The confidence interval of the rate of change of CFU in the supernatant includes zero.

**Appendix 1—figure 7.**
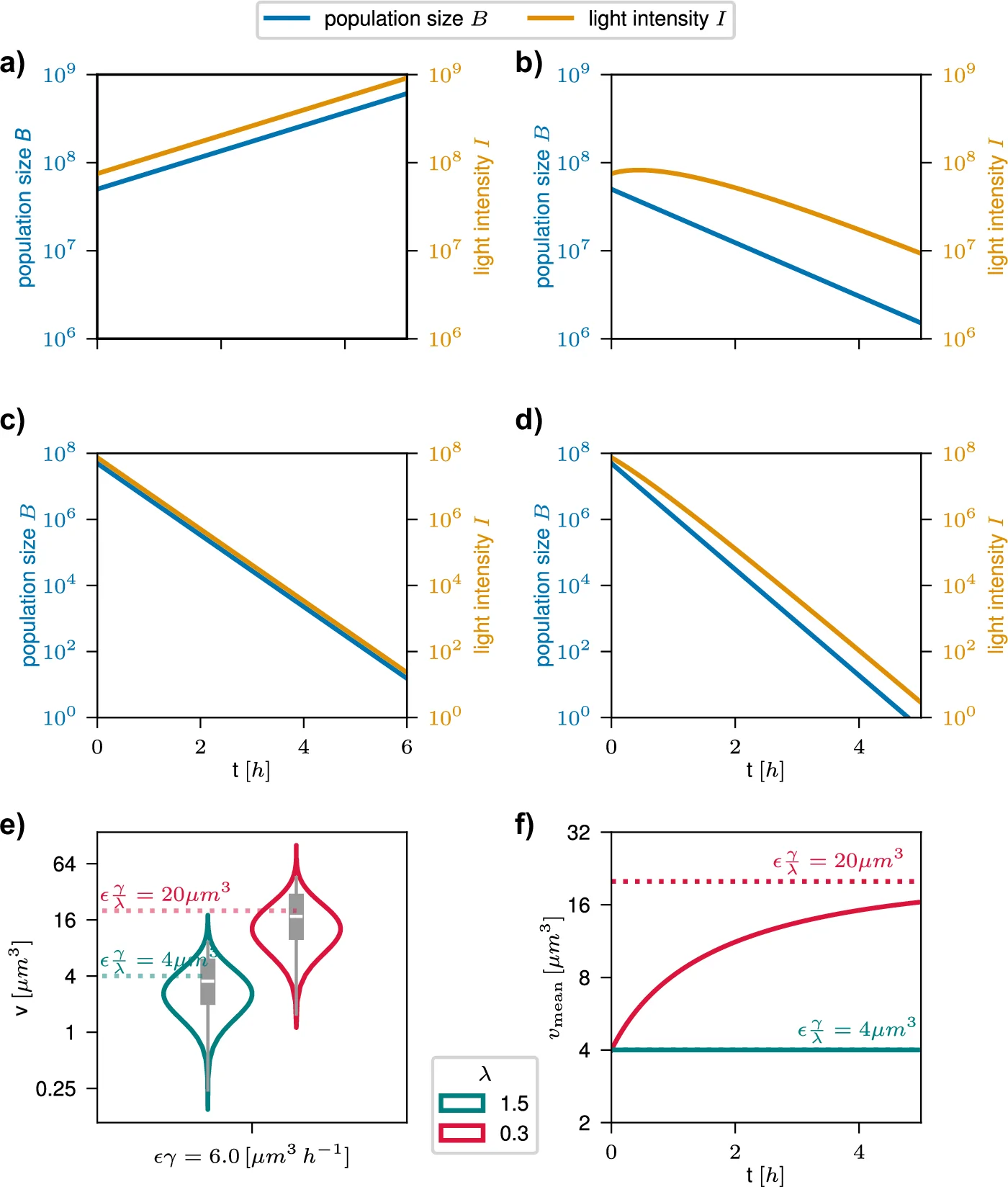
Illustrative simulations using the filamentation model relating (**a**–**d**) bacterial population size (blue) and light intensity (orange) under different combinations of treatment-induced changes in division rate ($\Delta\lambda$) and death rate (δ). Panel (**e**) shows the distributions of converged cell volumes for λ=0.3 and λ=1.5. Panel (**f**) shows the shift of mean cell volumes over time for $\Delta\lambda=-1.2$ and $\Delta\lambda=0$. For all simulations, we used $\lambda_{0}=1.5$, γ=150, ϕ=0.015, and ϵ=0.04. As shown in panel (**b**), treatment-induced filamentation can lead to a temporary discrepancy between luminescence- and CFU-based rates.

## Data Availability

Experimental datasets are available at Zenodo (https://doi.org/10.5281/zenodo.21321592). Analysis scripts, plate-handling worklists, colony-recognition code, and model code are available at Zenodo (https://doi.org/10.5281/zenodo.21321595, Muetter, 2025). The following dataset was generated: MuetterM 2026Experimental Data For the Luminescence ExperimentsZenodo10.5281/zenodo.21321592

## Additional files

MDAR checklist

## Appendix 1

### Mathematical descriptions

#### Light-related terminology

In this manuscript, *total luminosity* (L) refers to the total light output produced by B bacteria of a *bioluminescent* bacterial culture in a well with volume $V_{w}$. During the *luminescence assays*, we capture a fraction κ of the total luminosity (ϕB) as light intensity $I(t)=\kappa\phi B$. We call the exponential decline rates based on these intensities *luminescence-based rates*, $\psi_{I}$. We use *cell-specific luminosity* (ϕ) for light output per cell and *volume-specific luminosity* (θ) for light output per unit cell volume. When I(t) is normalized by the measured optical density, we obtain ω, the *OD-normalized light intensity*. J(t) is the light intensity I(t) adjusted by the relative change in cell volume $\frac{v_{0}}{v(t)}$.

#### Testing for linearity between bacterial density and luminescent light intensity

To test whether bacterial density and bacterial luminescent light intensity are linearly related, we grew three replicate overnight cultures. To replenish nutrients, we diluted each culture 1:10 in fresh LB medium and incubated for 60 min. We subsequently performed a 10-fold dilution series in a 384-well white plate (Greiner, 781073) and immediately measured the light intensity (see Appendix 1—figure 1). Each plate included wells containing only medium to determine a blank (median $\approx37.33\,\mathrm{r}\mathrm{l}\mathrm{u}$), which we subtracted from all measurements.

We assessed linearity between light intensity (I) and bacterial density (b) by fitting a linear model without intercept on the original scale,

$$\displaystyle I_{i}=m\cdot b_{i},$$

which corresponds to a log-log regression with intercept:

$$\displaystyle {\rm ln}\left(I_i\right) ={\rm ln}\left(m\right) + {\rm ln}\left(b_i\right).$$

Summing over all n observations gives:

$$\displaystyle  n\,\ln(m)=\sum_{i=1}^{n}\ln(I_{i})-\sum_{i=1}^{n}\ln(b_{i}),$$

The optimal conversion factor m is then:

$$\displaystyle m=\frac{\left(\prod_{i=1}^{n}I_{i}\right)^{\frac{1}{n}}}{\left(\prod_{i=1}^{n} b_{i}\right)^{\frac{1}{n}}},$$

yielding $m=0.006\;\frac{\mathrm{rlu}\,\mathrm{mL}}{\mathrm{CFU}}$.

We observed a linear relationship ($R^{2}=0.987$, F=1164.14, $\rm p < 10^{-14}$) between bacterial density and light intensity for intensities above $20\,\mathrm{r}\mathrm{l}\mathrm{u}$ ($\sim 3\times 10^{2}\,\mathrm{CFU/mL}$). Consequently, we use $20\,\mathrm{r}\mathrm{l}\mathrm{u}$ as the lower detection limit for luminescence. Additionally, we conclude that κ is independent of the bacterial density (no overshadowing effects), within the relevant range of densities. Since the plate reader setup remains constant within an experiment, we assume κ to be constant for following analyses.

#### Rate of change of CFU count

To infer the rate of change of CFU, we assume bacteria spend only a short time in the low-nutrient dilution medium, so replication and death are negligible during that phase.

Most colonies originate from clusters only comprising a single cell, but some from founding clusters of multiple cells. Let f(n) be the probability that a randomly chosen cluster contains n bacteria. A cluster of size n forms a colony with probability

(1) $$\displaystyle   1-(p_{E})^{n},$$

where $p_{E}$ is the probability that a lineage originating from a single bacterium goes extinct (extinction probability). The mean probability that a plated cluster forms a colony is:

(2) $$\displaystyle   \bar g=\sum_{n=1}^{N}f(n)\,(1-(p_{E})^{n}).$$

The mean cluster size is:

(3) $$\displaystyle   \bar n=\sum_{n=1}^{N}n\,f(n). $$

The mean number of colonies emerging per plated bacterium can be approximated by:

(4) $$\displaystyle   \eta=\frac{\bar g}{\bar n}, \hspace{2cm}\eta \in [0, 1]. $$

The predicted CFU per mL given a bacterial density $b=\frac{B}{V_{w}}$, with B the number of bacteria per well and $V_{w}$ the constant well volume, is:

(5) $$\displaystyle   \mathrm{CFU}= \eta \frac{B}{V_{w}}.$$

Taking the logarithmic derivative yields:

(6) $$\displaystyle   \psi_{\mathrm{CFU}}= \frac{d}{dt}\ln(\mathrm{CFU}) = \frac{d}{dt}\ln(\eta) + \frac{d}{dt}\ln(B) . $$

In practice η is often unknown. We can only estimate $\psi_{B}$ from CFU data if we assume that η is constant over time.

(7) $$\displaystyle   \psi_{\mathrm{CFU}}= \frac{d}{dt}\ln(B) = \psi_{B}.$$

To justify a constant η, we must assume that the cluster-size distribution f(n) and the extinction probability $p_{E}$ do not change over time. Both assumptions may fail, e.g., if cells filament or if the division or death rates change. We discuss the behavior of $p_{E}$ below.

#### Rate of change of luminescence

The observed light intensity I is a fraction κ of the total luminosity (ϕB) of a bioluminescent culture, where ϕ is the cell-specific luminosity (the amount of light emitted by one bacterium). The light intensity can thus be written as:

(8) $$\displaystyle   I = \kappa \phi B.$$

The rate of change of light intensity is therefore:

(9) $$\displaystyle   \psi_{I} = \frac{d}{dt}\ln(I) = \frac{d(\ln(\kappa) + \ln(\phi) + \ln(B))}{dt}.$$

We assume that κ remains constant over time, as we explained above (‘Testing for linearity between bacterial density and luminescent light intensity’). If we assume that the cell-specific luminosity ϕ is also constant, $\psi_{I}$ equals the rate of bacterial count change $\psi_{B}$, as Equation 9 simplifies to:

(10) $$\displaystyle   \psi_{I} = \frac{d}{dt}\ln(I) = \frac{d}{dt}\ln(B) = \psi_{B}.$$

#### Rate of change of volume-corrected luminescence

Alternatively, we can link the measured light intensity to the number of bacteria using the mean cell-specific volume (v) and the volume-specific luminosity (θ):

(11) $$\displaystyle   I = \kappa \theta v B. $$

Defining the volume-corrected luminescence as $J(t)=I(t)v_{0}\big/v(t)$, we can compute its rate of change as:

(12) $$\displaystyle   \psi_{J} = \frac{d(\ln(I \cdot v_{0}/v))}{dt}= \frac{d(\ln(\kappa) + \ln(v_{0}) + \ln(\theta) + \ln(B))}{dt}. $$

If we assume that the volume-specific luminosity θ is constant, this estimate equals the rate of change of the number of living bacteria (B):

(13) $$\displaystyle   \psi_{J} = \frac{d}{dt}\ln(I \cdot v_{0}\big/v) = \frac{d}{dt}\ln(B) = \psi_{B}.$$

#### Change of light intensity is closer to the rate of change of total cell volume than to the rate of change of number of bacteria

We made two empirical observations under all tested drug conditions:

(i) *For the subset of drugs imaged using microscopy, the mean specific cell volume never significantly decreased between the first and second time point*:

(14) $$\displaystyle   \frac{d\ln v}{dt}\geq 0$$

The rate of change of total cell volume is given by:

(15) $$\displaystyle   \psi_{V} =\frac{d\ln(vB)}{dt}=\frac{d\ln v}{dt}+\psi_{B}. $$

From this relation and observation (i), we directly obtain:

(16) $$\displaystyle   \boxed{\psi_V \ge\psi_B}$$

(ii) *The rate of change of volume-corrected light intensity was never significantly lower than the corresponding rate of change of CFU*:

(17) $$\displaystyle   \psi_{J}=\frac{d\ln J}{dt}\ge\psi_{\mathrm{CFU}}$$

Since we can express the light intensity as $I=\kappa\theta vB$, the volume-corrected luminescence rate becomes:

(18) $$\displaystyle   \psi_{J} = \frac{d}{dt}\ln(\kappa\theta v_{0} B)=\psi_{B}+\frac{d\ln\theta}{dt}.$$

Observation (ii) thus implies:

(19) $$\displaystyle   \psi_{B}+\frac{d\ln\theta}{dt}\geq \psi_{\mathrm{CFU}}$$

If we make the assumption that the rate of change of CFU equals that of bacterial count ($\psi_{\mathrm{CFU}}=\psi_{B}$), we obtain:

(20) $$\displaystyle   \frac{d\ln\theta}{dt}\geq 0 $$

Using the rate of change of light intensity, we get:

(21) $$\displaystyle   \psi_{I} = \frac{d\ln(\kappa\theta vB)}{dt}= \psi_{V} + \frac{d\ln\theta}{dt}.$$

From this relation and Equation 20, we follow:

(22) $$\displaystyle   \boxed{\psi_I \ge \psi_V}$$

Combining Equation 16 and Equation 22 yields:

(23) $$\displaystyle   \boxed{\psi_I \ge \psi_V \ge \psi_B}$$

allowing us to conclude that, during our experiments, the luminescence-based rate is closer to the rate of change of total cell volume (likely identical to the rate of change of biomass) than to the rate of change of bacterial count.

#### Colony formation – birth-death Markov model

We use a basic birth-death Markov model, as described by Coates et al., 2018, Novozhilov et al., 2006, Kendall, 1948, and Feller, 1939, to model the probability that a single plated bacterium creates a colony.

In this model, as in Coates et al., 2018, $P_{0}(t)$ describes the probability that the population originating from this single bacterium goes extinct by the time t:

(24) $$\displaystyle   P_{0}(t) = \frac{\delta}{\lambda}\cdot \frac{E(t) - 1}{E(t) - \frac{\delta}{\lambda}},$$

where δ is the death rate, λ the division rate, and $E(t)=e^{(\lambda-\delta)t}$. For $t\to\infty$, $P_{0}(t)$ converges to the extinction probability $p_{E}$ for a lineage originating from a single cell.

(25) $$\displaystyle   p_{E} = \begin{cases}1, & \text{if } \lambda \leq \delta, \\ \frac{\delta}{\lambda}, & \text{if } \lambda > \delta.\end{cases}$$

#### Colony formation for bacteriostatic and bactericidal drugs

In the following, we call the death and division rate in the absence of treatment $\delta_{0}$ and $\lambda_{0}$, respectively, and the treatment-induced increase in death and reduction in division rate $\delta_{T}$ and $\lambda_{T}$, respectively. We then rewrite the net growth rate as:

(26) $$\displaystyle   \psi = \lambda_{0} - \lambda_{T} - \delta_{0} - \delta_{T}$$

Furthermore, we define the combined treatment effect:

(27) $$\displaystyle   \tau = \psi_{0} - \psi = \delta_{T} + \lambda_{T}$$

We write the probability of colony formation (from a single cell) as:

(28) $$\displaystyle   p_{E} = \begin{cases}1,&\text{if }\lambda_{0} - \lambda_{T} \leq \delta_{0} + \delta_{T}, \\ \frac{\delta_0 + \delta_T}{\lambda_0 - \lambda_T},&\text{if }\lambda_{0} - \lambda_{T} > \delta_{0} + \delta_{T}.\end{cases}$$

We then define the extinction probability for purely bacteriostatic drugs ($\delta_{T}=0$ and $\lambda_{T}=\tau$) as:

(29) $$\displaystyle   p_{E, \mathrm{stat}}= \begin{cases}1,&\text{if }\lambda_{0} - \tau \leq \delta_{0}, \\ \frac{\delta_0}{\lambda_0 - \tau},&\text{if }\lambda_{0} - \tau > \delta_{0}.\end{cases}$$

We define the extinction probability for purely bactericidal drugs ($\delta_{T}=\tau$ and $\lambda_{T}=0$) as:

(30) $$\displaystyle   p_{E, \mathrm{cidal}}= \begin{cases}1,&\text{if }\lambda_{0} \leq \delta_{0} + \tau, \\ \frac{\delta_0 + \tau}{\lambda_0},&\text{if }\lambda_{0} > \delta_{0} + \tau.\end{cases} $$

In Appendix 1—figure 3, we plot the colony formation probability for a single bacterium plated on agar ($p_{C}=1-p_{E}$) as a function of the treatment effect τ, showing purely bacteriostatic (red) and purely bactericidal (blue) drugs.

### Filamentation model

To model bacterial filamentation, we discretize the cell volumes into K classes indexed by $i\in\{1,2,\dots,K\}$. Each class contains the bacterial density $b_{i}(t)$ of cells with volume

(31) $$\displaystyle   v_{i} = i\,\epsilon,$$

where ϵ is a unit volume increment. We define the following rules for growth, division, and death of cells

- cells in class i=1 cannot divide
- cells in class i=K cannot grow
- cells in class i=1,…,K−1 shift from class i to i+1 at rate γ
- cells in class i=2,…,K divide at rate λ, resulting in a redistribution of cells from class i into smaller classes (e.g. if i is even, two cells appear in class i/2; if i is odd, one cell each appears in classes $\tfrac{i-1}{2}$ and $\tfrac{i+1}{2}$).
- cells in all classes die at rate δ.

We collect the populations into a vector

$$\displaystyle   \vec{b}(t) = \bigl(b_{1}(t),\,b_{2}(t),\,\dots,\,b_{K}(t)\bigr)^{\mathsf{T}},$$

and write the dynamics as:

(32) $$\displaystyle   \frac{d\vec{b}}{dt}= \lambda\,\Lambda\,\vec{b}+ \gamma\,\Gamma\,\vec{b}- \delta\,\vec{b},$$

where Λ and Γ are transition matrices for division and growth, respectively. An example form for Γ (volume acquisition) is:

$$\displaystyle   \Gamma = \begin{pmatrix}-1 & 0 & 0 & 0 & \cdots & 0 & 0\\ 1 & -1 & 0 & 0 & \cdots & 0 & 0\\ 0 & 1 & -1 & 0 & \cdots & 0 & 0\\ \vdots & \vdots & \vdots & \vdots & \ddots & \vdots & \vdots\\ 0 & 0 & 0 & 0 & \cdots & 1 & 0\end{pmatrix},$$

which shifts cells from class i to i+1. An example Λ (division) might be:

$$\displaystyle   \Lambda = \begin{pmatrix}0 & 2 & 1 & 0 & \cdots & 0 \\ 0 & -1 & 1 & 2 & \cdots & 0 \\ 0 & 0 & -1 & 1 & \cdots & 0 \\ \vdots & \vdots & \vdots & \vdots & \ddots & \vdots \\ 0 & 0 & 0 & 0 & \cdots & -1\end{pmatrix}.$$

We assume that the volume of the two new cells after division is identical to the original volume of the parent cell before division:

(33) $$\displaystyle   \sum_{i=1}^{K}i\,(\Lambda\, \vec{b})_{i} = 0.$$

Furthermore, the volume acquisition does not impact the number of cells:

(34) $$\displaystyle   \sum_{i=1}^{K}(\Gamma\, \vec{b})_{i} = 0.$$

#### Population-level quantities

We define the total bacterial density across all volume classes as:

(35) $$\displaystyle   b(t)=\sum_{i=1}^{K}b_{i}(t),$$

and *total biovolume density*:

(36) $$\displaystyle   V(t)=\epsilon\sum_{i=1}^{K}i\,b_{i}(t)=\sum_{i=1}^{K}v_{i}\,b_{i}(t).$$

Then, the *mean cell volume* is:

(37) $$\displaystyle   v(t)=\frac{V(t)}{b(t)}.$$

In the finite model, boundary effects arise because the smallest cells cannot divide and the largest cannot grow. In the continuum limit, $\epsilon\to 0,\;K\to\infty$, these effects vanish and all cells experience uniform rates, so the following equalities hold:

(38) $$\displaystyle   \frac{db}{dt}=(\lambda-\delta)\,b,$$

(39) $$\displaystyle   \frac{dV}{dt}=\epsilon\,\gamma\,b-\delta\,V,$$

(40) $$\displaystyle   \frac{dv}{dt}=\epsilon\,\gamma-\lambda\,v.$$

Setting $\tfrac{dv}{dt}=0$ yields the equilibrium mean volume

(41) $$\displaystyle   v_{\mathrm{eq}}=\frac{\epsilon\,\gamma}{\lambda}.$$

Integrating Equation 38 gives

(42) $$\displaystyle   b(t)=b_{0}\exp\bigl((\lambda-\delta)t\bigr).$$

Substituting this result into Equation 39 and integrating with the integrating factor $\exp(\delta t)$ yields the analytic solution for the total biovolume density across all size classes:

(43) $$\displaystyle   V(t)=v_{\mathrm{eq}}\,b(t)+\bigl(V_{0}-v_{\mathrm{eq}}\,b_{0}\bigr)\exp(-\delta t).$$

Finally, writing $v_{0}=V_{0}\big/b_{0}$, we obtain the analytical solution for the mean cell volume:

(44) $$\displaystyle   v(t)=v_{\mathrm{eq}}+\bigl(v_{0}-v_{\mathrm{eq}}\bigr)\exp(-\lambda t).$$

#### Parameter sensitivity

We evaluated the impact of changes in the division rate, $\Delta\lambda\in[-1.5,0.5]$, and the death rate, $\delta\in\{0,2,4\}$, on the difference between the luminescence-based rate and the true net growth rate. To this end, we set the treatment-free division rate to $\lambda_{0}=1.5$, the treatment-free death rate to $\delta_{0}=0$, the biovolume acquisition rate in the presence and absence of treatment to $\gamma=150\rm hr^{-1}$, the size of a volume increment to ε = 0.04μm^3^, and volume-specific luminosity to θ=0.015. We simulated each parameter set for 4 hr using K=1000. Furthermore, we added the estimate based on luminescence if the first 2 hr of data are excluded.

### Volume correction and parameter estimation

To correct the light signal for dynamic changes in biovolume, we combine two sources of information: (i) two morphology snapshots before ($v_{\mathrm{obs},0}$) and after 2 hr of treatment ($v_{\mathrm{obs},2\mathrm{h}}$), and (ii) the luminescence time series $I_{\mathrm{obs}}(t_{i})$.

We first rewrite Equation 44 using the equilibrium-to-initial volume ratio $\alpha=v_{\mathrm{eq}}/v_{0}$ as:

(45) $$\displaystyle   v(t) = \alpha v_{0}+ (1 - \alpha)\, v_{0}\, \mathrm{e}^{-\lambda t}.$$

To avoid fitting α as a free parameter, we insert $v_{\mathrm{obs},0}$ and $v_{\mathrm{obs},2\mathrm{h}}$ into Equation 45 to express α as a function of λ:

(46) $$\displaystyle   \alpha(\lambda) = \frac{v_{\mathrm{obs},2\mathrm{h}}- v_{\mathrm{obs},0}\mathrm{e}^{-2\lambda}}{v_{\mathrm{obs},0}(1 - \mathrm{e}^{-2\lambda})}, \qquad \alpha > 0.$$

Assuming constant volume-specific luminosity, we insert this constrained α into Equation 11 to obtain:

(47) $$\displaystyle   I(t) = I_{0}\, \mathrm{e}^{(\lambda - \delta)t}\left[ \alpha + (1 - \alpha)\, \mathrm{e}^{-\lambda t}\right],$$

with scale parameter $I_{0}=\kappa\theta v_{\mathrm{obs},0}b_{0}V_{w}$.

To estimate the division rate λ and death rate δ, we eliminate the nuisance parameter $I_{0}$ by defining

(48) $$\displaystyle   F_{i} := \mathrm{e}^{(\lambda - \delta)t_i}\left[ \alpha + (1 - \alpha)\, \mathrm{e}^{-\lambda t_i}\right],$$

so that

(49) $$\displaystyle   \ln I(t_{i}) = \ln I_{0} + \ln F_{i}.$$

Minimizing the residual sum of squares over $\ln I_{0}$ yields the optimal

(50) $$\displaystyle   \ln I_{0}^{\ast} = \overline{\ln I_{\mathrm{obs}}}- \overline{\ln F},$$

where the overline denotes the sample mean across all i.

Minimizing the residual $\ln I(t_{i})-\ln I_{\mathrm{obs}}(t_{i})$ by substituting Equation 49 and Equation 50 yields the final loss function:

(51) $$\displaystyle   \mathcal{L}(\lambda, \delta) = \sum_{i=1}^{n}\left[ \left(\ln F_{i} - \overline{\ln F}\right) - \left(\ln I_{\mathrm{obs}}(t_{i}) - \overline{\ln I_{\mathrm{obs}}}\right) \right]^{2}.$$

To balance the dataset of observed specific volumes v, we sample 200 values per replicate and pool them. We then use the same bootstrapped light intensity datasets as in the main method for estimating $\psi_{I}$. For each bootstrap sample, we randomly pair one $v_{\mathrm{obs},0}$ and one $v_{\mathrm{obs},2\mathrm{h}}$ with one luminescence trajectory and minimize Equation 51 over the biologically plausible region:

$$\displaystyle 0.01\leq\lambda\leq 1.75,\qquad\delta\geq 0.$$

The remaining quantities $\psi=\lambda-\delta$, $\alpha(\lambda)$, and $I_{0}^{\ast}$ are computed algebraically.

### Experiments

#### SOS experiment

We conducted this experiment to test whether activating the SOS response by UV light would increase the specific luminosity and thereby explain the shallower decline of light intensity compared to the decline in CFU counts. This hypothesis rests on the possibility that the phage promoter driving the lux cassette upregulates when the cell experiences stress.

To test this, we diluted three replicate overnight cultures 1:100 and grew them for approximately 1.5 hr to mid-exponential phase. Each of the three exponential-phase cultures was split into two aliquots: one was assigned to UV treatment and placed in the upper half of a white 96-well plate (rows B–D), while the other served as an untreated control in the lower half (rows E–G) (Greiner, 655098).

We started the experiment by measuring luminescence and OD in the plate reader. Then, we alternated between exposing the strains for repeated intervals to UV light in a cross-linker (Hoefer, UVC 500 crosslinker, at 10 μJ/cm^2^) in a temperature-regulated environment (36.5°C); followed by luminescence and OD measurements. During UV exposure, we shielded the control samples by covering the lower half of the plate with a metal lid. The durations of UV treatment were 30 s, 1 min, 2 min, 4 min, and 8 min.

OD increased in both UV-treated and control cultures; however, UV exposure visibly impaired OD growth compared to the controls (Appendix 1—figure 2a). We normalized the luminescence signals (Appendix 1—figure 2b) by dividing through the OD signal, resulting in the OD-normalized light intensity ω (Appendix 1—figure 2c). We observed that the OD-normalized light intensity of UV-treated cultures falls with the duration of treatment compared to the OD-normalized light intensity of the controls ($\omega_{\mathrm{UV}}-\omega_{\mathrm{ctrl}}$; see Appendix 1—figure 2d; t-test, $\rm p=3\cdot 10^{-5}$ for the last time point).

Based on these results, we find it unlikely that upregulation of the promoter explains the shallower decline of light intensity compared to that of CFU count. However, we cannot exclude the possibility that this result does not hold if the SOS response is triggered by another mechanism.

#### Morphology evaluation

We analyzed the microscopy images in several steps. First, we manually applied lower and upper thresholds to the red and green channel to enhance the visual contrast (Figure 2—figure supplements 2–14). Next, we used ‘Ilastik-1.4.0’ to infer the probability that each pixel in the thresholded green channel image belonged to a bacterium. Ilastik employs a neural network trained directly on the microscopy images.

Subsequently, we used a Python script (Muetter, 2025) to convert these probabilities into markers representing individual bacteria. Misidentified markers were manually excluded, e.g., if they only partially covered a bacterium or covered multiple overlapping bacteria. For each marker, we fitted a spline through its center, providing the spline length $l_{s}$. We then optimized the radius $r_{s}$ by maximizing the marker area within a distance $r_{s}$ from the spline while minimizing the area within $r_{s}$ that did not belong to the marker.

Using these parameters, we calculated for each bacterium the length $l_{b}=l_{s}+2\cdot r_{s}$, width $w_{b}=2\cdot r_{s}$, and volume $v=2\cdot\pi\cdot r_{s}^{2}\cdot l_{s}+\frac{4}{3}\cdot\pi\cdot r_{s}^{3}$. To create a volume distribution for each treatment, we resampled the fitted volume estimates from each image (replicate) 200 times with replacement, preserving the original sample size, and aggregated the resulting datasets. Based on these distributions, we determined whether cells were significantly filamented using the significance criterion described in the Materials and methods section of the main paper.

#### AMP deactivation experiment

To assess whether pexiganan-treated bacteria continued to die in a 1:100 diluted PBS environment, we exposed exponential-phase cultures to 16 μg/mL pexiganan for 1 min. Following this treatment, 10 μL of each culture was diluted in 990 μL of PBS supplemented with 0, 1, 10, or 100 mM CaCl_2_ or MgCl_2_. Every 45 min, we sampled from each diluted culture and plated 10 μL aliquots using the automated plating method described in the Materials and methods section of the main paper.

We observed a substantial effect of both supplements on the measured bacterial density (Appendix 1—figure 4a and b). Increasing the supplement concentration consistently resulted in higher bacterial densities, indicating that the supplemented ions reduce bacterial killing. Most data points for the unsupplemented medium resulted in empty agar plates. The highest CFU count was observed for strains diluted in 100 mM MgCl_2_ (Appendix 1—table 4).

#### Manual pexiganan time-kill curve experiment

In this setup, we captured four time points within 5 min using CFU plus the pretreatment bacterial density. The experiment was performed manually using the traditional CFU plating method. Round agar plates (Sarstedt, 82.1473.001) containing 25 mL of agar were used, and 100 μL of each dilution was plated. This approach increases sensitivity by using 100 μL, rather than 10 μL, for plating.

Cultures were treated in a 96-deepwell plate (Greiner, 780285) by adding 100 μL of a 10× stock to 900 μL of exponential-phase culture. Samples were taken directly from the deepwell plate, and two simultaneous dilution series were prepared in a 96-well plate (Greiner, 655101) at each time point to halt the killing. One dilution series was prepared in pure PBS, and the other in PBS supplemented with 100 mM MgCl_2_.

For this experiment, we plated three dilutions (factors of 100, 1000, and 10,000) and counted the colonies on all plates. We observed that when PBS without MgCl_2_ was used, higher dilution factors led to higher CFU estimates (Appendix 1—figure 5). This discrepancy diminished over time, in parallel with a weakening of the observed kill rate. In contrast, when the dilution medium was supplemented with MgCl_2_, we did not observe this effect.

#### Supernatant experiment

During the measured pexiganan kill curve described above, we observed a steep decline in CFU counts, followed by a nearly constant plateau. Two non-exclusive explanations may account for this observed decrease in killing: (i) the surviving bacteria are persisters or resistant to the AMP or (ii) the AMP molecules become deactivated, leaving the supernatant without killing activity.

To investigate the supernatant’s remaining bactericidal effects, we conducted a multi-step experiment:

Step 1: Preparation. Three overnight (O/N) cultures were diluted 1:100 in LB and incubated at 37°C with shaking for 2 hr. From each culture, we took three samples: one to measure the bacterial density before treatment, the second (1 mL) to accumulate pure bacteria for supernatant exposure, and the third 1.35 mL was reserved for supernatant production and measuring the initial kill rate.

Step 2: Purification of bacteria. To purify bacteria, we pelleted the previously collected 1 mL bacterial aliquots at $3000\,\times{g}$ for 5 min, discarded the supernatant, and stored them in the fridge.

Step 3: Initial killing and supernatant production. To generate the supernatant, each 1.35 mL sample was treated with a 160 μg/mL pexiganan stock at a 1:10 ratio, yielding a final concentration of 16 μg/mL. After 5 min, we plated samples for CFU counts and centrifuged the remaining culture at maximum speed for 2 min to remove cellular debris. Plate counts of these treated samples revealed rapid bacterial killing (rate of CFU count change $\psi_{\mathrm{CFU}}\approx-46$ per hr) (Appendix 1—figure 6, Appendix 1—table 5). We collected 1 mL of the clarified supernatant for the subsequent exposure experiment.

Step 4: Supernatant killing. In that final step, we dissolved the pelleted bacteria (from step 2) in the supernatant collected during step 3. After another 5 min incubation, we plated samples (dilutions 1:100, 1:1000, and 1:10,000) to estimate bacterial density. No significant killing was observed, indicating that the supernatant alone no longer exhibited bactericidal activity – supporting explanation (ii), without rejecting (i).

Our current hypothesis is that AMP molecules bind to the surface of intact bacteria and to newly exposed targets from lysed bacteria, thereby becoming deactivated. Thus, cells that survive the initial kill phase may have an increased chance of continued survival. Possible explanations for why specific bacteria survive this phase include reduced surface area due to clumping or adhesion to well walls, smaller cell size, and other factors that might confer protection, potentially related to the cell cycle.

**Appendix 1—table 1.** Drugs used in this study, their minimum inhibitory concentrations (MICs), working concentrations, and stock solvents. In the MIC column, we report the highest concentration of the dilution series (numerator) and the maximum inhibiting dilution (denominator). Kanamycin (50 μg/mL) was used as the selection marker for the *lux* operon.

Drug	MIC[μg/mL]	cwork 10 μg/mL	cwork [MIC]	Solvent	Supplier
Amoxicillin	$\frac{10}{4}=2.50$	25	10	DMSO	Sigma, A8523
Ampicillin	$\frac{100}{128}=0.78$	10, 2	12.8, 2.56	Water	Sigma, A9518
Cefepime	$\frac{4}{256}=0.02$	0.15	9.6	DMSO	Thermo Fisher, J66237
Ceftazidime	$\frac{4}{64}=0.06$	0.62	10	DMSO	Sigma, PHR1847
Cefuroxime	$\frac{4}{2}=2.00$	16	8	Water	Sigma, C4417
Chloramphenicol	$\frac{128}{64}=2.00$	20	10	DMSO	Sigma, C0378
Ciprofloxacin	$\frac{1}{128}=0.01$	0.08	10	Water	Sigma, 17850
Colistin	$\frac{50}{64}=0.78$	1.6	2.05	Water	Sigma, C4461
Doripenem	$\frac{4}{256}=0.02$	0.16	10.24	Water	VWR, ACRO463870010
Fosfomycin	$\frac{4}{4}=1.00$	8	8	Water	VWR, APOSBIM0107
Imipenem	$\frac{5}{64}=0.08$	1	12.8	Water	Sigma, PHR1796
Mecillinam	$\frac{10}{128}=0.08$	0.78	10	DMSO	Sigma, 33447
Meropenem	$\frac{5}{512}=0.01$	0.1	10.24	Water	Sigma, PHR1772
Penicillin	$\frac{500}{32}=15.62$	100	6.4	Water	Roth, HP48.2
Pexiganan	$\frac{64}{32}=2.00$	8, 16	4, 8	Water	Sigma, SML3787
Piperacillin	$\frac{10}{16}=0.62$	6.25	10	DMSO	Sigma, J66419
Polymyxin B	$\frac{50}{32}=1.56$	2.5	1.6	Water	Roth, 0235.1
Rifampicin	$\frac{40}{16}=2.50$	25	10	DMSO	Sigma, R3501
Tetracycline	$\frac{10}{32}=0.31$	3.12	10	DMSO	Sigma, T3383
Trimethoprim	$\frac{10}{128}=0.08$	0.78	10	DMSO	Sigma, T7883

**Appendix 1—table 2.** Point estimates and 95% percentile intervals of $\psi_{\mathrm{CFU}}$, $\psi_{I}$, $\psi_{I}^{*}$, and $\psi_{J}$ for different treatments. Sig_x_ indicates whether the rate of change of signal $X\in\{I,I^{*},J\}$ differs significantly (*) from the distribution of $\psi_{\mathrm{CFU}}$, or not (n.s.), based on the significance criterion defined in the Materials and methods section. Estimates are based on data from the colony-forming unit (CFU)−luminescence assays (see Materials and methods).

	$\psi_{\mathrm{CFU}}\left[\frac {1}{\rm h}\right]$	$\psi_{I}\left[\frac {1}{\rm h}\right]$	sig*_I_*	$\psi_{I}^{*}\left[\frac {1}{\rm h}\right]$	sig*_I_**	$\psi_{J}\left[\frac {1}{\rm h}\right]$	sig_J_
**Ampicillin** 10 μg/mL	−3.23 (−4.11, −2.54)	−2.41 (−2.91, −2.11)	*			−2.63 (−3.31, −2.29)	n.s.
**Ampicillin** 2 μg/mL	−0.81 (−1.19, −0.37)	−0.30 (−0.41, −0.22)	*				
**Amoxicillin** 25 μg/mL	−2.15 (−2.42, −1.88)	−2.04 (−2.21, −1.86)	n.s.				
**Cefepime** 0.15 μg/mL	−1.17 (−1.50, −0.85)	−0.47 (−0.74, −0.24)	*	−0.88 (−1.13, −0.65)	*		
**Ceftazidime** 0.62 μg/mL	−0.88 (−1.06, −0.71)	−0.09 (−0.24, 0.04)	*			−0.38 (−0.52, −0.15)	*
**Cefuroxime** 16 μg/mL	−1.59 (−2.41, −0.99)	−1.41 (−1.64, −1.25)	n.s.	−1.62 (−1.92, −1.34)	n.s.		
**Chloramphenicol** 20 μg/mL	0.05 (−0.11, 0.20)	0.04 (−0.03, 0.10)	n.s.				
**Ciprofloxacin** 0.078 μg/mL	−1.96 (−2.29, −1.64)	0.83 (0.44, 1.17)	*			0.48 (0.09, 0.90)	*
**Colistin** 1.6 μg/mL	−1.00 (−1.45, −0.52)	−0.93 (−1.26, −0.66)	n.s.				
**Doripenem** 0.16 μg/mL	−0.54 (−0.83, −0.25)	−0.24 (−0.37, −0.15)	*	−0.37 (−0.53, −0.26)	*		
**Fosfomycin** 8 μg/mL	−1.22 (−1.65, −0.83)	−1.12 (−1.37, −0.93)	n.s.				
**Imipenem** 1 μg/mL	−1.42 (−2.06, −0.72)	0.00 (−0.16, 0.14)	*	−0.32 (−0.45, −0.22)	*		
**Mecillinam** 0.78 μg/mL	−0.32 (−0.57, −0.10)	0.02 (−0.17, 0.17)	*	−0.34 (−0.47, −0.20)	n.s.		
**Meropenem** 0.1 μg/mL	−1.85 (−2.36, −1.32)	−0.16 (−0.47, 0.10)	*			−0.55 (−0.80, −0.21)	*
**Penicillin** 100 μg/mL	−2.17 (−2.78, −1.66)	−2.20 (−2.53, −1.94)	n.s.				
**Piperacillin** 6.25 μg/mL	−0.26 (−0.51, 0.02)	0.09 (−0.01, 0.19)	*				
**Polymyxin B** 2.5 μg/mL	−1.45 (−1.73, −1.22)	−1.41 (−1.87, −1.07)	n.s.				
**Rifampicin** 25 μg/mL	−0.23 (−0.40,0.04)	−0.15 (−0.23,−0.07)	n.s.				
**Tetracycline** 3.125 μg/mL	−0.06 (−0.12, 0.00)	−0.01 (−0.07, 0.05)	n.s.				
**Trimethoprim** 0.78 μg/mL	−0.61 (−0.76, −0.47)	0.48 (0.38, 0.59)	*			0.34 (0.18, 0.51)	*
**Pexiganan** 8 μg/mL	−44.59 (−66.19, −20.08)	−45.95 (−48.21, −43.34)	n.s.				
**Pexiganan** 16 μg/mL	−61.63 (−93.40,1.26)	−60.27 (−63.06, −56.42)	n.s.				

**Appendix 1—table 3.** Bootstrapped 95% confidence intervals and point estimates for the length, width, and volume of cells after 2 hr of treatment, estimated from microscopy images. Significance was assessed by comparing the confidence intervals of cell volumes for each antibiotic treatment to the untreated control (control_2 hr), as described in the Materials and methods section of the main paper.

Treatment	Length (μm) mean (95% CI)	Width (μm) mean (95% CI)	Volume (μm^3^) mean (95% CI)	V-sig.
Control_2h	**3.41** (2.28, 4.92)	**1.09** (0.77, 1.40)	**3.89** (2.00, 6.51)	Ref.
Amoxicillin	**5.09** (2.64, 10.77)	**1.17** (0.47, 1.37)	**6.21** (1.93, 9.67)	n.s.
Ampicillin	**12.36** (3.87, 27.14)	**1.27** (0.93, 1.67)	**16.67** (5.70, 30.39)	Significant
Ceftazidime	**54.54** (32.05, 68.69)	**0.95** (0.67, 1.22)	**40.42** (11.52, 80.86)	Significant
Ciprofloxacin	**21.77** (10.88, 36.82)	**1.03** (0.70, 1.27)	**19.55** (6.35, 45.70)	Significant
Colistin	**3.14** (2.19, 4.53)	**1.07** (0.60, 1.59)	**3.71** (1.00, 8.32)	n.s.
Fosfomycin	**3.18** (1.77, 5.32)	**0.93** (0.69, 1.15)	**2.65** (1.09, 4.75)	n.s.
Meropenem	**5.90** (3.44, 10.61)	**2.26** (1.13, 3.71)	**31.11** (5.33, 71.01)	Significant
Rifampicin	**4.77** (2.41, 8.38)	**0.98** (0.59, 1.40)	**4.32** (1.15, 9.44)	n.s.
Tetracycline	**4.71** (2.43, 7.70)	**1.08** (0.65, 1.71)	**5.25** (1.29, 13.40)	n.s.
Trimethoprim	**12.19** (4.67, 30.74)	**0.93** (0.65, 1.29)	**9.05** (2.64, 19.79)	Significant

**Appendix 1—table 4.** Estimated group means and 95% confidence intervals from an ordinary least squares (OLS) model fitted to log-transformed colony-forming unit (CFU) data, collected from the first sampled time point after diluting pexiganan-treated strains in supplemented phosphate-buffered saline (PBS). Grouping is based on the supplement (CaCl_2_ or MgCl_2_) and concentration (0 mM to 100 mM). Confidence intervals were computed using heteroscedasticity-consistent standard errors (HC3). The compact letter display (cld) indicates groups that are not significantly different by sharing a common letter, based on mutual inclusion of their 95% confidence intervals.

Group	Mean	Low	Up	cld
CaCl_2_(0 mM)	−0.00	−0.00	0.00	a
CaCl_2_ (1 mM)	2.77	−0.56	6.09	b
CaCl_2_ (10 mM)	5.06	4.92	5.20	c
CaCl_2_ (100 mM)	5.85	5.38	6.31	d
MgCl_2_ (0 mM)	−0.00	−0.00	0.00	a
MgCl_2_ (1 mM)	2.77	−0.56	6.09	b
MgCl_2_ (10 mM)	5.31	5.20	5.42	e
MgCl_2_ (100 mM)	6.36	6.27	6.46	f

**Appendix 1—table 5.** Comparison of kill rates [$h^{-1}$] between cultures treated with pexiganan (16 μg/mL) for 5 min and cultures exposed to the supernatant collected after the kill assay. The two rates differ significantly; the confidence interval of the rate of change of colony-forming unit (CFU) in the supernatant includes zero.

Experiment	Mean	Low	Up
Pexiganan	−46.98	−63.15	−35.80
Supernatant	1.28	−2.19	5.11

**Appendix 1—table 6.** Primer sequences used for λ-Red-mediated integration of the *luxCDABE* operon into *E. coli*. Lowercase letters indicate homology regions binding to the *lux* operon on the plasmid; uppercase letters indicate chromosomal homology regions at the integration site.

Primer	Sequence (5’ → 3’)
Forward	CGGTACGGCTGACCATCGGGTGCCAGTGCGGGAGTTTCGTacccagtaaggcagcggtatc
Reverse	AGTCAGCGATATCCATTTTCGCGAATCCGGAGTGTAAGAAtaggtctagggcggcgga
