# Supplementary material for: High-throughput quantification of population dynamics using luminescence
Source: eLife. 2026 Aug 5;15:RP109213. doi: 10.7554/eLife.109213 (PMC13441296; doi:10.7554/eLife.109213)
Supplement: Peer review history

# Editor report: eLife Assessment

**DOI:** 10.7554/eLife.109213.3.sa0

**Authors:** Wenying Shou

**Affiliations:** University College London United Kingdom

**Keywords:** Convincing, Important

Muetter et al. provide an **important** argument that luminescence is a reliable, high-throughput alternative to colony-forming units (CFU) for super-MIC investigations, particularly when the quantity of interest is biomass. By examining 20 antimicrobials spanning 11 classes, the work shows that discrepancies between CFU and luminescence are often biological (filamentation, Viable But Not Culturable). The work provides a **convincing** view of how these three common measurements (luminescence, optical density, and CFU) relate to one another across a range of drug treatments, although testing on clinical isolates could be of further benefit.

# Referee report: Reviewer #2 (Public review)

**DOI:** 10.7554/eLife.109213.3.sa1

Summary:

In antibiotic research, accurately measuring decreases in bacterial populations is essential. The authors conducted a comprehensive evaluation of the luminescence assay, a commonly used but previously under-quantified method, benchmarking it against the gold-standard CFU counting approach. They found that luminescence measurements generally aligned with CFU results but sometimes reported slower decline rates for certain antimicrobials. These discrepancies were linked to differences in how the two methods capture biomass and colony formation, which vary with the antimicrobial's mechanism of action. The study demonstrates that luminescence assays can serve as a high-throughput alternative to labor-intensive CFU counting, provided their limitations are understood and corrected.

Strengths:

The authors developed a mathematical model to partially correct luminescence-based measurements, making the approach broadly applicable to several commonly used antibiotics. They also analyzed antibiotic-treated single-cell morphologies and linked filamentation to bulk luminescence signals. This analysis helped define the range of drug conditions under which luminescence assays provide reliable estimates of bacterial dynamics.

They extensively evaluated the method using 20 antibiotics and one antimicrobial peptide, encompassing many of the most commonly used agents and experimental factors (e.g. treatment time) typically considered in antibiotic research.

Comments on revised version:

No further comments. The authors have adequately addressed my concerns.

# Referee report: Reviewer #3 (Public review)

**DOI:** 10.7554/eLife.109213.3.sa2

Summary:

This preprint proposes luxCDABE-based luminescence as a high-throughput alternative (or complement) to CFU time-kill assays for estimating antimicrobial rates of population change at super-MIC concentrations, by comparing luminescence- and CFU-derived rates across 20 antimicrobials (22 assays) and attributing divergences primarily to filamentation (luminescence closer to biomass/volume than cell number) and changes in culturability / carryover (CFU undercounting viable cells).

Strengths:

The authors do not merely report discrepancies; they experimentally validate the biological causes. Specifically, they successfully attribute the slower decline of luminescence in certain drugs to bacterial filamentation (maintaining biomass despite halted division) and the rapid decline of CFU in others to loss of culturability or carryover effects.

The inclusion of 20 antimicrobials spanning 11 classes provides a robust dataset that allows for broad categorization of drug-specific assay behaviors.

The study critically exposes flaws in the "gold standard" CFU method, specifically regarding antimicrobial carryover (demonstrated with pexiganan) and the potential for CFU to overestimate cell death in the presence of VBNC (viable but non-culturable) states induced by drugs like ciprofloxacin.

The use of chromosomal integration for the lux operon to minimize plasmid copy-number effects and the validation of linearity between light intensity and cell density establish a solid technical foundation.

In summary:

Muetter et al. provide a compelling argument that luminescence is a reliable, high-throughput alternative to CFU for super-MIC investigations, particularly when the quantity of interest is biomass. The paper effectively warns researchers that discrepancies between CFU and luminescence are often biological (filamentation, VBNC) rather than methodological failures.

Comments on revised version:

The revised version addressed my comments well.

# Author comment: Author response

**DOI:** 10.7554/eLife.109213.3.sa3

**Authors:** Malte Muetter, Daniel C Angst, Roland Regoes, Sebastian Bonhoeffer

**Affiliations:** ETH Zurich Zürich Switzerland; ETH Zurich Zurich Switzerland; ETH Zurich Zurich Switzerland; Institute of Integrative Biology, Department of Environmental Systems Science, ETH Zürich Zurich Switzerland

The following is the authors’ response to the original reviews.

**Public Reviews:**

**Reviewer #1 (Public review):**

Summary:

This study examines how luminescence can be used to measure bacterial population dynamics during antimicrobial treatment by comparing it directly with optical density and colony counts. The authors aim to determine when luminescence reflects changes in population size and when it instead captures metabolic or physiological states induced by drug exposure. By generating parallel datasets under controlled conditions, the work provides a detailed view of how these three common measurements relate to one another across a range of drug treatments.

Strengths

The study is technically strong and thoughtfully designed. Measuring luminescence, optical density, and colony counts from the same cultures allows the authors to make clear and informative comparisons between methods. The data are compelling, and the analyses highlight both agreements and divergences in a way that is easy to interpret. The manuscript also succeeds in showing why these divergences arise. For example, the observation that filamentation and metabolic shifts can sustain luminescence even when colony counts drop provides valuable information on how different readouts capture distinct aspects of bacterial physiology. The writing is clear, the figures are effective, and the work will be useful for researchers who need high-throughput approaches to quantify microbial population dynamics experimentally.

Weaknesses:

The study also exposes some inherent limitations of luminescence-based measurements. Because luminescence depends on metabolic activity, it can remain high when cells are damaged or unable to resume growth, and it can fall quickly when drugs disrupt energy production, even if cells remain physically intact. These properties complicate interpretation in conditions that induce strong stress re-sponses or heterogeneous survival states.

In addition, the use of drug-free plates for colony counts may overestimate survival when filamented or stressed cells recover once the antibiotic is removed, making differences between luminescence and colony counts harder to attribute to killing alone. Finally, while the authors discuss luminescence in the context of clinically relevant concentration ranges, the current implementation relies on engineered laboratory strains and does not directly demonstrate applicability to clinical isolates. These limitations do not detract from the technical value of the work but should be kept in mind by readers who wish to apply the method more broadly.

We thank the reviewer for reading our paper thoroughly and for the helpful feedback.

Luminescence limitations. We agree that the lack of a direct link between light intensity and a population property such as biomass or cell number is the main limitation of the luminescence method. To further emphasise this, we have expanded the Discussion in the revised manuscript.

Drug-free plates. The use of drug-free plates is intentional. As we measure a time series, the question at each point is how many cells are alive at each time point. Cells that are stressed but viable at time *t* contribute correctly to the count at *t*. How long they survive under the respective treatment is captured by the subsequent timepoints.

Filaments. Recovery of plated filamented cells should not inflate this estimate. A single plated filamentous cell is expected to yield either zero (death before division) or one single colony, regardless of in how many parts it separates, as all descendants are part of the same cluster. However, if the cells divide before plating, CFU can overestimate survival. Having that said, we have no indication that this occurred in our experiments, since in all observed discrepancies, CFU-based estimates were equal to or lower than those obtained from luminescence and the time cells spent in dilution was kept short.

Clinical applicability. We agree with the reviewer that the method is not practical for ad-hoc pharmacodynamic studies of clinical isolates. What we instead provide is an *E. coli*-based model system to explore clinically relevant treatment conditions, which we address in the revised manuscript. We believe that constructing analogous bioluminescent model strains in other clinically relevant species would be a valuable direction for future work.

**Reviewer #2 (Public review):**

Summary:

This preprint proposes luxCDABE-based luminescence as a high-throughput alternative (or complement) to CFU time-kill assays for estimating antimicrobial rates of population change at super-MIC concentrations, by comparing luminescence- and CFU-derived rates across 20 antimicrobials (22 assays) and attributing divergences primarily to filamentation (luminescence closer to biomass/volume than cell number) and changes in culturability/carryover (CFU undercounting viable cells).

Strengths:

The authors do not merely report discrepancies; they experimentally validate the biological causes. Specifically, they successfully attribute the slower decline of luminescence in certain drugs to bacterial filamentation (maintaining biomass despite halted division) and the rapid decline of CFU in others to loss of culturability or carryover effects.

The inclusion of 20 antimicrobials spanning 11 classes provides a robust dataset that allows for broad categorisation of drug-specific assay behaviours.

The study critically exposes flaws in the “gold standard” CFU method, specifically regarding antimicrobial carryover (demonstrated with pexiganan) and the potential for CFU to overestimate cell death in the presence of VBNC (viable but non-culturable) states induced by drugs like ciprofloxacin.

The use of chromosomal integration for the lux operon to minimise plasmid copy-number effects and the validation of linearity between light intensity and cell density establish a solid technical foundation.

Weaknesses:

The study is conducted exclusively using *Escherichia coli*. While *E. coli* is a standard model organism, the paper claims to evaluate luminescence as a generalisable high-throughput tool. Many of the discrepancies observed are driven by filamentation. However, distinct morphological responses occur in other critical pathogens (e.g., *Staphylococcus aureus* does not filament in the same way).

The authors propose that luminescence data can be corrected using microscopyderived volume data to better align with CFU counts. The primary appeal of luminescence is high-throughput efficiency. If a researcher must perform timelapse microscopy to calculate cell volume changes to “correct” their luminescence data, the high-throughput advantage is lost.

The paper argues that for ciprofloxacin, CFU underestimates viability because cells remain intact and impermeable to propidium iodide. While the cells are metabolically active and membrane-intact, if they cannot divide to form a colony (even after drug removal/dilution), their clinical relevance as “living” pathogens is debatable.

Some other comments:

The use of a population dynamical model to simulate filamentation effects is excellent. The finding that light intensity tracks volume ($\psi_V$) better than cell number ($\psi_B$) is a key theoretical contribution.

The model assumes linear elongation. The authors should briefly comment on whether this holds true for the specific drug mechanisms tested (e.g., PBP inhibition vs. DNA gyrase inhibition).

The use of bootstrapping to estimate rate distributions is appropriate and robust.

Conclusion:

Muetter et al. provide a compelling argument that luminescence is a reliable, highthroughput alternative to CFU for super-MIC investigations, particularly when the quantity of interest is biomass. The paper effectively warns researchers that discrepancies between CFU and luminescence are often biological (filamentation, VBNC) rather than methodological failures.

We thank the reviewer for reading our paper thoroughly and for the helpful feedback.

Generalisability. We agree that the alignments and divergences reported for specific drugs may not transfer directly to other species, which may elongate differently (e.g. cocci) or show different physiological responses to treatment. Constructing analogous model strains — for example based on *S. aureus* to cover a broader range of morphologies and clinically relevant species would therefore be an interesting follow-up project, and we have adjusted the Discussion to make this clearer. We nevertheless believe that the broader conclusions (larger cells emit more light) of the paper likely hold across species.

Volume correction. We agree that requiring microscopy would undermine the high-throughput advantage of the luminescence assay. It was not our intention to propose this as a practical approach, nor to imply that the luminescence signal needs a correction. Taken on its own, the signal can be interpreted as the cumulative metabolic output of the population, which is closely linked to biomass, and that measure is valuable in itself for many applications. We used the volume correction only to demonstrate that luminescence tracks biomass more closely than cell number: by adjusting the luminescence distribution with the measured volume change, it moves towards the CFU distribution. We have revised the Discussion to prevent this from being misunderstood as a required step.

Culturability vs. clinical relevance. We agree that the dynamics of culturable cells are highly relevant, especially in a clinical context. Our aim was to explain the observed differences between CFU and luminescence by highlighting that culturability and viability are not always identical, without implying that one measure is inherently superior to the other — we leave it to the reader to decide which metric best suits their needs.

Linear elongation. The model assumes linear elongation for mathematical convenience, which, depending on the specific strain and drug mechanism, could be incorrect. Its purpose is to demonstrate that a shift of the mean cell volume to a new, higher equilibrium under treatment can cause an initial peak in the luminescence signal despite a declining population. This remains true for non-linear elongation models, though the shape, height and position of the peak may change. We have adjusted the Results to make this clearer.

**Recommendations for the authors:**

**Reviewer #1 (Recommendations for the authors):**

(1) The authors present luminescence as a practical measurement of population decline under antibiotic exposure. One aspect that could be clarified is how the method behaves when tolerance arises from phenotypic heterogeneity, such as the presence of small, metabolically quiet survivors. Because luminescence reflects metabolic activity and biomass, the signal will be dominated by metabolically active cells, making rare tolerant subpopulations difficult to detect. A short discussion of how luminescence performs in these heterogeneous scenarios, and whether complementary assays are needed to capture long-lived tolerant cells, would strengthen the manuscript.

Yes, that is a valid concern and we thank the reviewer for raising this point.

Heterogeneity in cell-specific luminosity alone does not bias population-level rate estimates. A bias can arise, however, when specific luminosity correlates with a second factor — most importantly, the decline rate under treatment.

We agree with the reviewer’s suggestion that brighter cells plausibly die faster than tolerant, metabolically quiet ones. When one subpopulation dominates the light signal, we expect minimal bias, as the rate estimate primarily reflects that subpopulation. However, in a transition phase when both subpopulations contribute roughly equally to the light signal, luminescence likely overestimates the decline.

We added a corresponding caveat to the Discussion (lines 581–583).

(2) The manuscript shows that filamentation can influence ψ_I by altering biomass and metabolic activity independently of cell number. However, antibiotic exposure can also trigger other stress responses and metabolic shifts that change energy fluxes, redox balance, and biosynthetic activity. Since luminescence depends on metabolic state and substrate availability, these additional physiological transitions may also affect ψ_I in ways not directly tied to birth or death processes. It would be useful to comment on whether such responses, beyond filamentation, are likely to influence luminescence dynamics across different drug classes or treatment conditions.

We thank the reviewer for raising this point and agree that there is no biological law strictly linking luminosity to a single population property such as biomass or cell number, and changes in the metabolism most likely affect *Ψ_I_* as well.

Transitioning to a new metabolic steady state biases *Ψ_I_*; once the new steady state is reached, however, the rate estimate should no longer be affected.

Looking across drug classes, drugs that primarily lyse cells (polymyxins and, to a lesser degree, beta-lactams targeting PBP1) did not show noticeable deviations between *Ψ_I_* and *Ψ_CFU_*, and — perhaps counterintuitively — neither did ribosome-inhibiting drugs.

For the remaining cases, we were able to attribute part of the discrepancy between *Ψ_I_* and *Ψ_CFU_* to changes in biomass or loss of culturability, though drug-induced metabolic changes may also contribute to the residual differences.

We clarify this in the Discussion (lines 569–579).

(3) The authors quantify survival using colony counts on drug-free medium. Because filamentation can be a reversible state that persists during antibiotic exposure, plating on drug-free medium may capture recovery potential rather than in-treatment viability. Filamented or stressed cells that cannot divide in the presence of a drug may nevertheless form colonies once the drug is removed. Clarifying how this recovery step affects ψ_CFU would help readers interpret differences between luminescence-based and colony-based measurements, especially in cases where transient tolerant states are present.

We thank the reviewer for raising this point.

Our CFU assay estimates the number of culturable cells at each time point; the rate *Ψ_CFU_* is then inferred from how this number changes across time points. Plating on drug-free medium is intentional, as it maximises the probability that a culturable cell is detected at each snapshot. Whether those cells would have continued dividing or died under continued treatment is captured by the subsequent time points.

Filamentation interacts with the probability of colony formation in several, partly opposing ways:

(1) It can increase the death rate, as for ceftazidime and cefepime, which is part of the kill effect captured by *Ψ_CFU_*;

(2) Entanglement between filaments may reduce the number of colonies per plated bacterium;

(3) Conversely, if a filament divides upon drug removal, its fragments form a cluster that — stochastically — is very likely to produce one (but not multiple) colony.

The only scenario in which CFU could overestimate bacterial density is if a filament separates into individual cells in the liquid phase before plating; we have no indication that this occurred in our experiments.

We addressed this concern in our response to the public comment.

(4) A brief discussion comparing luminescence to fluorescent reporter systems could be helpful. Fluorescent proteins typically require a chromophore maturation step before becoming detectable, which introduces a delay between the underlying cellular event and the appearance of the signal. In contrast, as far as I understand, lux reporters emit light immediately once the enzymatic components and substrates are present, without a maturation stage. Highlighting this distinction may help readers understand why luminescence is well-suited for tracking rapid changes in population physiology under antibiotic exposure. However, the manuscript also notes that luminescence can lag slightly behind very rapid killing (particularly for AMPs), but the temporal dynamics of signal shutdown are not explored in detail. Because lux reflects metabolic activity rather than viability, a short delay between irreversible damage and the loss of light is biologically expected. It may help readers if the authors could expand on the mechanism underlying this delay in order to clarify when ψ_I is likely to track true biomass decline and when residual metabolic activity might mask early killing events.

On fluorescent reporters:

We thank the reviewer for this suggestion.

Under some conditions, change rates can also be measured using fluorescence, provided the number of fluorescent molecules per bacterium remains constant. This requires a balance between production, maturation, degradation and dilution, which is only established if the growth rate and conditions remain constant over a sufficiently long period (typically hours).

For measuring population decline, however, the key issue is that cell death does not inactivate fluorescent proteins: once matured, they emit independently of the cell’s metabolic state and decay only with the protein’s half-life, which is typically slower than the kill rates of interest.

We added a clarification to the Introduction (lines 58–60).

On the lux signal lag:

We thank the reviewer for raising this point. The short lag between luminescence and CFU decline could in principle arise from two mechanisms: (i) luminescence declining more slowly than the actual cell number (residual light from dead cells), or (ii) CFU declining more steeply than the actual cell number (damaged but still viable cells failing to form colonies).

Mechanism (i) splits into two sub-cases:

(i.a) Dead but impermeable — the lux reaction could in principle continue for a short while if enough components are retained in the cell. However, a metabolically active, impermeable cell is difficult to classify as dead in the first place, making this scenario conceptually awkward.

(i.b) Dead and permeable (lysed) — the lux components dilute into the medium, and by mass-action the reaction rate should drop rapidly (though not instantly). Any residual signal after lysis should therefore be short-lived.

Mechanism (ii) — damaged (e.g. permeable) cells may be particularly sensitive to plating on agar (e.g. due to oxidative stress), resulting in a declining probability of colony formation.

In our case, the discrepancy was observed specifically for pexiganan, where cells can be assumed to lyse, making (i.b) and/or (ii) the likely explanations. Based on our experimental data, we cannot distinguish between these possibilities and therefore limit ourselves to reporting the observed discrepancy.

We have moved the interpretation from the Results to the Discussion (lines 523–542) and expanded the discussion there.

(5) In lines 85–89, the authors state that “high-throughput OD and luminescence measurements at sub-MIC concentrations provide valuable insights into drug effects on growth rates, [but] the super-MIC range is clinically more relevant,” and they present luminescence as a way to investigate super-MIC population dynamics. While super-MIC behaviour is indeed important for pharmacodynamics and resistance evolution, it is not clear that the specific luminescence implementation used here has direct clinical relevance. The study relies on a chromosomally integrated reporter in a laboratory strain, and the manuscript does not demonstrate that this approach can be applied to clinical isolates or diagnostic workflows. It may be helpful to moderate the claim of “clinical relevance” and frame the method more clearly as a high-throughput experimental tool that can inform clinically relevant questions, rather than as an assay ready for clinical application.

We agree and have moderated the framing accordingly (lines 100–104).

**Reviewer #2 (Recommendations for the authors):**

(1) The conclusions regarding “biomass vs. cell number” may not apply equally to non-rod-shaped bacteria or species with different stress responses. The authors must explicitly discuss this limitation in the Discussion.

The broad conclusion that bigger cells emit more light likely holds across morphologies, since it rests on the principle that more cellular material means more metabolic activity and therefore more light. The quantitative relationship between cell size and luminosity, however, may differ across species, shapes and conditions, for two reasons. First, chromosome copy number: whether drug-induced morphological changes are accompanied by chromosome replication and therefore an increase in *lux* operon copy number — varies across species and drug mechanisms. Second, the surface-to-volume ratio likely modulates mass-specific metabolism; some morphological changes preserve it (e.g. purely lateral elongation) while others do not.

The more specific conclusions about which drug classes produce alignment or divergence between CFU and luminescence may also not transfer directly, as drug mechanisms can act differently across species.

We already note this limitation in the Discussion (lines 594– 597) and have expanded the wording there.

(2) The manuscript should clarify that luminescence is a superior metric for biomass without correction, rather than framing the volume correction as a necessary step to mimic CFU. The divergence should be embraced as a feature (biomass tracking), not a bug that needs fixing via labor-intensive microscopy.

We agree with the framing and will make it clearer; it was actually our intention to clarify which method does what, rather than judge one as better or worse.

We removed the “correction” sentence from the Discussion to make this clearer.

(3) The authors should refrain from definitively stating CFU “underestimates” viability and instead use more precise terminology, such as “reproductive capability” vs. “metabolic integrity.”

We agree with the reviewer that measuring culturability is a property, not a flaw, of CFU. Our intention was to emphasise that when CFU is used as a proxy for viability (which it often is), it can yield lower values than the actual number of survivors. We tried to make that distinction explicit in the manuscript (e.g. in lines 317–322).

We would also like to note that in the case of antimicrobial carryover, CFU can genuinely underestimate culturability itself, not only viability.

Regarding the suggested reproductive capability vs. metabolic integrity framing: we agree that metabolism and luminescence are closely linked. What held us back from drawing that link directly is that metabolism is hard to quantify, being the cumulative output of a diverse set of processes.

(4) The model assumes linear elongation. The authors should briefly comment on whether this holds true for the specific drug mechanisms tested (e.g., PBP inhibition vs. DNA gyrase inhibition).

Linear elongation is a mathematically convenient simplification whose only purpose in the model is to allow the population to converge to a new equilibrium volume under treatment. Assuming constant volume-specific luminosity, we showed that this produces an initial peak in light intensity before the signal declines in parallel with *Ψ_B_*. The exact shape, height and position of this peak depend on the volume growth model used, but the qualitative pattern — peak followed by parallel decline — holds for other growth models as well. We now clarify this in lines 230–235.

(5) The authors suggest the carryover effect is due to a delay between cell death and cessation of luminescence. This “lag time” is a critical physical constraint of the lux system (likely related to ATP depletion or enzyme decay) and should be quantified or discussed in more detail as a fundamental “speed limit” for the assay.

The origin of the lag between luminescence and CFU is an interesting question, but one we cannot definitively answer. We can, however, discuss the potential mechanisms:

A dead but impermeable cell could in principle continue to emit residual light for some time. We note, though, that calling a metabolically active, impermeable cell “dead” is a question of definition we would rather not discuss here.

In our case, the discrepancy was observed specifically for pexiganan, where cells can be assumed to lyse. Under lysis, the lux components dilute quickly into the medium, and by mass-action the reaction rate should drop rapidly — though not necessarily instantaneously.

A plausible alternative to a delayed cessation of the light signal is that the probability of colony formation drops rapidly after permeabilisation, for example because permeable cells are sensitive to oxidative stress when plated on agar.

Based on our experimental data we cannot distinguish between these mechanisms, so we limit ourselves to reporting the observed discrepancy. We have moved the interpretation from the Results to the Discussion (lines 523–542) and expanded on the candidate mechanisms there.

Additional revisions

Beyond the changes prompted by the reviewers’ comments, we made the following revisions to the supplementary information:

We corrected the Λ matrix (converted row 2, col 4 from 0 → 2)

We removed the line numbering
